# Supplementary material for: A cluster randomized trial assessing the effect of a digital health algorithm on quality of care in Tanzania (DYNAMIC study)
Source: PLOS Digit Health. 2024 Dec 23;3(12):e0000694. doi: 10.1371/journal.pdig.0000694 (PMC11666054; doi:10.1371/journal.pdig.0000694)
Supplement: S1 File — (PDF) [file pdig.0000694.s002.pdf]

[redacted]

[redacted]

*In accordance with PLOS's copyright policy, the following logos were taken out: DYNAMIC study, Ifakara Health Institute, Unisanté, Swiss Tropical and Public Health Institute, Mbeya Medical Research Center - National Institute of Medical Research

STUDY PROTOCOL

Dynamic clinical decision support algorithms to manage sick children in primary health care settings in Tanzania (DYNAMIC TZ)

**Protocol Number**	NIMR/HQ/R.8a/Vol.IX/3486		
**Version Number**	5.0	**Document Date**	07.09.2022
**Sponsor Contact**	Centre for Primary Care and Public Health (Unisanté)<br>University of Lausanne<br>Bugnon 44 - 1011 Lausanne<br>Switzerland<br>www.unisante.ch		
**Principal Investigator**	Prof. Valérie D'Acremont<br>Centre for Primary Care and Public Health (Unisanté)<br>University of Lausanne<br>Bugnon 44 - 1011 Lausanne – Switzerland<br>+41 79 556 25 51<br>valerie.dacremont@unisante.ch; valerie.dacremont@swisstph.ch		
**National Principal Investigator**	Dr. Honorati Masanja<br>Ifakara Health Institute (IHI) http://ihi.or.tz<br>P.O Box 78373 - Dar es Salaam – Tanzania<br>hmasanja@ihi.or.tz		
**National co-Principal Investigator**	Dr. Nyanda Ntinginya<br>National Institute of Medical Research - Mbeya Medical Research Centre (NIMR-MMRC) http://www.nimr-mmrc.org<br>P.O Box 2410 - Mbeya – Tanzania<br>nelias@nimr-mmrc.org		
**Funding Agency**	- Fondation Botnar, St. Alban-Vorstadt 56 - 4052 Basel – Switzerland<br>- Swiss Development Cooperation, Bern – Switzerland		

The information contained in this document is confidential. It is intended solely for the Investigators, potential Investigators, consultants, or applicable Independent Ethics Committees and Regulatory Authorities. It is understood that this information will not be disclosed to others without prior written authorization from the Sponsor, except where required by applicable local laws.

I. List of Investigators and other persons involved

Names	Institution	Position	Function in study
Prof. Valérie D'Acremont	Unisanté	Head of Digital and Global Health, Department of Training, Research and Innovation	PI, overall project coordinator
	Swiss TPH	Scientific group leader, Medical Department	
Dr. Honorati Masanja	IHI	Chief Executive Director	National PI
Dr. Nyanda Ntinginya	NIMR-MMRC	Centre Director	National co-PI
Dr. Irene Masanja	IHI	Scientific project leader, Health Systems Research Group	Co-investigator, country coordinator
Dr. Kristina Keitel	Swiss TPH	Scientific project leader, Clinical Research Unit, Medical Department	Co-investigator, pediatric consultant
Dr. Mary-Anne Hartley	Unisanté and EPFL	Scientific project leader, Digital and Global Health, Department of Training, Research and Innovation	Co-investigator, ML coordinator
Dr. Chacha Mangu	NIMR-MMRC	Project manager, TB and Emerging Disease department	Mbeya site investigator
Dr. Alexandra V. Kulinkina	Swiss TPH	Project leader, Clinical Research Unit, Medical Department and SCIH	Co-investigator, Project coordinator
Dr. Lameck Luwanda	IHI	Study physician, Health Systems Research Group	Morogoro site investigator
Dr. Godfrey Kavishe	NIMR	Study physician, TB and Emerging Disease dep.	Study physician
Dr. Rainer Tan	Unisanté	Research physician, Digital and Global Health, Department of Training, Research and Innovation	Clinical algorithms coordinator
Charles Festo	IHI	Head of the IHI Data Systems Unit	Country IT and data coordinator
Vincent Faivre	Unisanté	Deputy head of IT department	IT coordinator and data manager
Peter Agrea	NIMR	Software developer, IT department	Software developer
Ibrahim Evans Mtebene	IHI	Software developer, IT department	Software developer
Geofrey Isdory Ashery	IHI	Research officer, Health Systems Research Group	Social scientist, Morogoro site
Happy Raphael Mkali	IHI	Research scientist, Bagamoyo research laboratory	Laboratory coordinator
Prof. Isabella Eckerle	University of Geneva	Head of the Geneva Centre for Emerging Viral Diseases	Surveillance coordinator
Alan Vonlanthen	Unisanté	Project Manager, IT department	Project manager
Dr. Tracy Glass	Swiss TPH	Scientific project leader, Clinical Statistics and Data Management, Medical Department	Statistician
Prof. Martin Jaggi	EPFL	Head of Machine learning and optimization lab	ML consultant
Dr. Sabine Renggli	IHI	Senior Research Scientist	Site investigator, Morogoro site
Martin Norris	Swiss TPH	Project Officer/Manager	Algorithm testing consultant

Dr. Lisa Crump	Swiss TPH	Veterinary Epidemiologist	Surveillance Consultant
Dr. Gillian Levine	Swiss TPH	Neonatal Epidemiologist	Neonatal algorithm consultant
Humphrey Mhagama	NIMR	Study nurse	Study nurse
Dr. Alix Miauton	Unisanté	Research physician	Clinical coordinator

Project Location: Morogoro and Mbeya Regions, Tanzania

Projection duration: Project starts on 15.09.2021; Project ends on 31.03.2024

II. Signatures

The signatures below confirm agreement by the individuals authorized by the sponsor and principal participating institutions at the sites that the study will be conducted in compliance with protocol version 4.9 dated 10.09.2021.

Principal Investigator

Signature		07.09.2022
Name	Prof. Valérie D'Acremont	Date of Signature
Job title	Head, Digital and Global Health, Department Training, Research and Innovation	
Institution	Centre for Primary Care and Public Health, University of Lausanne	
Address	Bugnon 44 - 1011 Lausanne, Switzerland	
Phone	+41 79 556 25 51	

Principal Investigator Tanzania

Signature	DocuSigned by: 728BE860CB8A417...	07.09.2022
Name	Dr. Honorati Masanja	Date of Signature
Job title	Chief Executive Director	
Institution	Ifakara Health Institute	
Address	P.O Box 78373 - Dar es Salaam, Tanzania	
Phone	+255 626 605 048	

Co-principal investigator Tanzania

Signature	DocuSigned by: Dr. Nyanda Elias Ntinginya 6F908436CC73443...	07.09.2022
Name	Dr. Nyanda Ntinginya	Date of Signature
Title	Centre Director	
Institution	National Institute of Medical Research - Mbeya Medical Research Centre	
Address	P.O Box 2410 - Mbeya, Tanzania	
Phone	+255 766 243 836	

III. Abbreviations / Glossary of terms

AMR	Antimicrobial Resistance
CDSA	Clinical Decision Support Algorithm
CHMT	Council Health Management Team
CRP	C-reactive protein
DHIS2	District Health Information System 2
EPFL	École Polytechnique Fédérale de Lausanne
ePOCT+	Electronic point of care tool plus
HF	Health facility
HCW	Health care worker
HMIS	Health Management Information System
IDSR	Integrated Disease Surveillance and Response
IHI	Ifakara Health Institute
IMCI	Integrated management of childhood illness
M&E	Monitoring and evaluation
ML	Machine learning
NIMR	National Institute of Medical Research
PHC	Primary health care
POC	Point-of-care
RDT	Rapid Diagnostic Test
Swiss TPH	Swiss Tropical and Public Health Institute
TZ	Tanzania
Unisanté	Centre for Primary Care and Public Health (University of Lausanne)
WHO	World Health Organization

IV. Synopsis

**Study Title**	**Dynamic clinical decision support algorithms to manage sick children in primary health care settings in Tanzania**
**Short Title**	**DYNAMIC TZ**
**Study Category**	**Implementation study to support clinical decision making and disease surveillance in sick children**
**Study rationale**	Children are a well-recognized vulnerable population that still suffers from a high rate of acute infectious diseases and preventable deaths. This is especially true in fragile health systems of Sub-Saharan Africa, where under-five mortality is 10 times higher than in high-income countries. The management of sick children at the primary care level in these environments remains of insufficient quality as front-line clinicians lack appropriate diagnostics, supervision to improve their skills, and decision support tools. Clinically validated POC diagnostic tests are often not available, and practice guidelines are quickly outdated by new evidence and changing epidemiology. When an epidemic arises, these static, generic guidelines can even become deleterious if the event is not detected on time and integrated into the recommendations.<br>In the absence of reliable guidance, HCWs tend to over-prescribe antibiotics. Approximately 9 out of 10 children at the primary care level in Tanzania receive an antibiotic, while only 1 in 10 needs one. Inappropriate antibiotic use disrupts the gut flora, favoring the proliferation of pathogens and weakening a child's immune response. It is also a major driver of antibiotic resistance, which is estimated to be responsible for up to 10 million deaths per year by 2050. Equally important to antibiotic overuse, is its underuse. Missing a child in need of antibiotic treatment or providing a child with an inappropriate type or dosage of antibiotic puts them at risk of preventable morbidity and death. The same occurs with antimalarials that are not always prescribed to the children in need: those with a positive malaria test result.<br>Misdiagnoses have consequences that reach beyond the patient. They increase re-attendance rates, further congesting PHC clinics and accruing economic losses not only for families but for the entire health system. Systematic errors in patient-level data accumulate, and as they are aggregated to measure population-level indicators, they have the potential to bias the statistics used to prioritize health interventions and, importantly, identify epidemics.<br>The WHO has identified digital health interventions and predictive tools in primary care as key accelerators in achieving the 2030 Sustainable Development Goal 3 of ensuring good health and well-being for all. New simple and cheap technologies, such as mobile devices, coupled with the advances in computing and data science, could help mitigate several of the aforementioned challenges. The proposed digital intervention is a third-generation CDSA intended to help HCWs at the primary care level manage children with acute illnesses. The first two versions of the algorithm have undergone rigorous evaluations in controlled research conditions as summarized below:<br>• The first-generation algorithm called ALMANACH was tested in Tanzania in 2010-2011, achieving improved clinical cure (from 92% to 97%) and a decrease in antibiotic prescription (from 84% to 15%) as compared to routine care. ALMANACH also led to more consistent clinical assessments without taking more time than a conventional consultation and was perceived by clinicians as "a powerful and useful" tool.<br>• The second-generation algorithm called ePOCT was trialed in Tanzania in 2014-2016. In addition to symptoms and signs, it made use of several POC tests to help detect children with severe infections requiring hospital-based treatment (oximetry and hemoglobin level) and/or children with serious bacterial infection (CRP). The use of ePOCT resulted in higher clinical cure (98%) as compared to ALMANACH (96%) and routine care (95%). The algorithm also further reduced antibiotic prescription to 11%, as compared to 30% with the use of ALMANACH and 95% in routine care.

	Electronic algorithms can thus be successfully implemented to improve clinical guidance and provide feedback to clinicians, as well as allow near-real-time analyses of data for M&E of health interventions, disease surveillance and outbreak detection. This project is in line with the Tanzanian Digital Health Investment Roadmap 2017-23, proposing provision of innovative software, IT infrastructure and skills to support quality health care. It also supports the National Action Plan on Antimicrobial Resistance 2017-22 that advocates for strengthening stewardship for antimicrobial use in HFs, as well as the National Malaria Strategic Plan 2014-20, which calls for introducing evidence-based, innovative diagnostic tools for malaria detection and differential diagnosis of other pathogens causing febrile illnesses. The disease surveillance component also supports the National Action Plan for Health Security 2017-2021 that aims at strengthening and sustaining the capacity of Tanzania to promptly detect and confirm outbreaks, the frequency and intensity of which may increase in the coming years due to climate change and population growth.
**Study objectives**	The goal of this study is to improve clinical diagnosis, decrease morbidity and mortality of children, and mitigate AMR using novel dynamic POC technologies that help front-line HCWs manage sick patients, enhanced by smart disease surveillance and outbreak detection mechanisms.<br><br>More specifically, this study seeks to:<br><br>**Objective 1:** Improve the **integrated management of children with an acute illness** through the provision of an electronic CDSA (ePOCT+) to clinicians working at primary care level;<br><br>**Objective 2:** Improve the accuracy of the clinical algorithm and **adapt it to spatiotemporal variations in epidemiology and resources,** based on the data generated through the ePOCT+ tool, analyzed using machine learning and checked by clinical experts;<br><br>**Objective 3:** **Enhance the district** (and national) **disease surveillance and outbreak detection capability** using the clinical data generated by the ePOCT+ tool complemented by targeted microbiological investigations and machine learning pattern detection;<br><br>**Objective 4:** **Enhance the district** (and national) **health management information system for monitoring and evaluation** and **conducting supportive supervision and mentorship** in health facilities using the clinical data generated by the ePOCT+ tool enhanced by additional data analysis and visualization dashboards;<br><br>**Objective 5:** **Create a framework** for the development and implementation of dynamic CDSA and disease surveillance tools, for large-scale, sustainable, and clinically responsible use of machine learning and data science.
**Study design**	The primary intervention study will be conducted in two phases. Additional cross-sectional mixed-methods studies will take place throughout the intervention period.<br><br>**Phase 1: cluster randomized controlled study in 40 health facilities** The ePOCT+ tool will be provided to HCWs in 20 of 40 primary HFs in two regions (Morogoro and Mbeya), while another 20 will serve as controls. The aim is to compare the effect of using ePOCT+ with routine case management of sick children on the rational prescription of antibiotics, while ensuring good clinical outcomes. Due to the pragmatic nature of the study, the design is adaptive in nature, where changes to the implementation may be incorporated throughout the study to account for issues encountered in monitoring data and feedback from the field. These changes apply to the implementation context and exclude significant adaptations of the algorithm.<br><br>**Phase 2: scale-up of the intervention in up to 100 health facilities and transformation into a dynamic algorithm**

The ePOCT+ tool will be extended to the 20 HFs serving as controls in Phase 1, as well as up to 60 additional neighboring facilities of our target area, to reach a total of around 100 facilities (60 in Morogoro and 40 in Mbeya). In Phase 2, an adaptive study design will be used to measure the same outcome indicators as in Phase 1. The medical content of the algorithm will not be fixed anymore, but rather modifiable. Each potential modification will first be evaluated by the Tanzanian clinical expert group for its clinical coherence, safety and potential benefit and then applied to the retrospective data. If these analyses confirm both a clinically relevant positive impact and estimate that there will be sufficient future cases during the study period to detect this improvement, the change in the algorithm will be tested in a randomized sub-study using the same study design as in Phase 1, except that randomization will take place at patient level rather than HF level. If the positive impact is confirmed in the sub-study, the modification will be implemented in all relevant locations/patient sub-groups. If the retrospective analyses confirm a positive and potentially clinically relevant impact of a modification where resource savings is also expected but there will not be sufficient power to formally test the change during the study period, the modification will be implemented and monitored based on the related outcome indicators. If the impact of the modification achieves the expected results given the accumulated data at the end of the study period, it will be retained.

Examples of algorithm modifications expected to have a positive impact that will be explored for implementation are: 1) adapting the frequency of malaria testing in areas with very low endemicity; 2) improving the accuracy of pneumonia diagnosis; 3) assessing the utility of using dengue rapid tests if an outbreak occurs; 4) or typhoid rapid tests in areas of high prevalence; 5) improving the prediction of severe disease; 6) improving the algorithm for children with chronic conditions (malnutrition, HIV or sickle-cell). Other type of changes might also be tested if considered important by the clinical experts.

Cross-sectional quality of care surveys:

Three cross-sectional surveys in a subset of randomly selected HFs will be performed to assess the quality of the consultation done by HCWs and their level of compliance with the algorithm. Among the 40 HFs included in Phase 1, 18 (10 in Morogoro and 8 in Mbeya, half interventions and half controls) will be randomly selected. 25 consultations per HF will be observed for a total 450 HCW/child pairs. The survey will be performed once before the start of the study, once during Phase 1 and again approximately 6 months later during Phase 2 in the same facilities, with the aims of assessing changes in the use of ePOCT+ over time in the initial intervention facilities and a before-and-after ePOCT+ analysis in the initial control facilities.

Syndromic surveillance enhanced by microbiological testing:

Syndromic surveillance (i.e. based on the clinical data generated by the use of ePOCT+) will be enhanced by microbiological surveillance in 6 to 8 sentinel HFs representative of different epidemiological profiles, as well as in any facility with abnormal data patterns detected by ML (based on predefined clinically supervised thresholds), to identify potential outbreaks. Depending on the spatiotemporal patterns in the syndromic surveillance data, samples could be taken in health facilities outside of the sentinel sites using a mobile surveillance team.

Mixed-methods operational research:

Throughout the pilot and Phases 1 and 2, as well as during the last year of the project, mixed-methods studies will be conducted with HCWs, patients and caregivers in HFs, in communities, as well as at district and national levels, to understand the contextual factors that facilitate or impede the implementation, sustainability and scale-up of digital health interventions such as ePOCT+ in PHC settings. Examples of such factors may include socioeconomic and ecological profiles of communities, working conditions of the clinicians in the HFs, patient satisfaction, among others. Data collection associated with these activities will be undertaken with special consideration of the HCWs’ time and workload and in a way that does not interfere with patient care.

**Study site**	The study will be conducted in Mlimba, Ifakara and Ulanga districts/councils (Morogoro Region) and in Mbeya Rural district and Mbeya city council (Mbeya Region).
**Study population**	All children aged 1 day to 14 years with an acute condition who present to the participating PHC facilities will be included. This differs from the first two generations of the algorithm that only included children 2 months to 5 years.<br>HCWs attending to these children, caregivers of these children, communities where these children live, and key actors of the health system (CHMTs, computer scientists and medical experts at national level) will be included in observational mixed-methods studies.
**Intervention**	The intervention will include **5 activities**, each supported by a specific electronic tool:<br>***A)*** *Acutely ill children will be assessed, diagnosed and treated by routine clinicians guided by the **ePOCT+** application loaded on a tablet or PC.*<br>The intervention will be undertaken in routine PHC facilities in Tanzania and entails the implementation of the ePOCT+ tool for patient assessment and management, with provision of associated POC tests. Healthcare providers will use ePOCT+ throughout their medical consultations for children aged 0-14 years presenting with an acute medical or surgical problem. The tool will not be used for managing emergency cases. These cases will be identified by the tool in the beginning of the consultation (triage stage) and referred immediately to a higher level health facility. The CDSA of ePOCT+ is based on applicable Tanzanian national and international guidelines for pediatric clinical management (TZ version of IMNCI 2014; WHO Pocket Book of Hospital Care for Children 2013; TZ National Guidelines for the Diagnosis and Treatment of Malaria 2014; TZ Standard Treatment Guidelines and Essential Medicines list for Children and Adolescents 2018; Standard Treatment Guidelines & National Essential Medicines List Tanzania Mainland 2017 (Adults);TZ National Guideline for Neonatal care and Establishment of Neonatal Care Unit 2019; TZ National Guidelines for the Management of Tuberculosis in Children 2016; WHO IMAI guidelines 2009). Before implementation, the medical algorithm will be reviewed by Tanzanian key experts in child health (Pediatric Association of Tanzania; Ministry of Health; Muhimbili National Hospital). Most of the algorithm content was previously validated in Tanzania (see background section). Specifically, the tool will guide clinicians on:<br>i) Decision on immediate referral to hospital versus home-based treatment<br>ii) Medical history (questions to ask caregivers and/or children)<br>iii) Physical exam (signs to look for)<br>iv) POC diagnostic tests (which should be performed in a given clinical context)<br>v) Diagnoses<br>vi) Treatments (including medicines and supportive treatments)<br>vii) Counseling (explanation on final diagnoses, treatments, and follow-up)<br>***B)*** *The algorithm will be regularly updated according to trends in the collected data. The modifications will be designed by clinical experts based on the clinical and microbiological surveillance data collected during the study. Approved changes will then be integrated into the algorithm using the **MedAL-Creator** tool that allows physicians without any IT/programming skills to modify the decision tree.*<br>During Phase 2, the modifications designed by the clinical expert group will be tested on retrospective data before being introduced in the electronic application. Each modification will then be loaded on the tablets or PCs of the relevant HCWs (for example of a certain region where an epidemic is occurring) with a message explaining the rationale and evidence behind this change in the algorithm.<br>***C)*** *HCWs will be trained through an initial in-person session and **an e-learning platform** available online (Phases 1 and 2)*<br>Initial centralized training on essential clinical skills, the evidence and guidelines behind the content of the algorithm, the rational use of antibiotics and the practicalities around the use of the electronic tool will be provided to clinicians working in

	intervention facilities. Re-training of incoming personnel and continuous training through an e-learning platform on the tablet or PC will also be implemented.<br>***D)*** *HFs and HCWs will receive supportive supervision and mentorship from the CHMT using the* ***medAL-monitor*** *tool (Phases 1 and 2)*<br>Targeted supervision to HFs by the CHMT will be organized based on the quality of care indicators calculated in real time from the uploaded data and made available through a web-based dashboard called medAL-monitor. Personalized feedback to HCWs and persons in charge of HFs will be provided for self-auditing.<br>***E)*** *The* ***medAL-outbreak*** *disease surveillance and outbreak detection system will be made available to the district (and national) health authorities (Phases 1 and 2) for them to be able to decide if disease control measures are needed*<br>Indicators for disease surveillance and early epidemic warning and response will be decided with and provided to CHMTs through a dedicated web-based dashboard (medAL-outbreak). These indicators can then be transferred to the DHIS2 platform and made available to national health authorities.
**Outcomes**	**Objective 1 (Improve integrated management of children with an acute illness):**<br>**Co-Primary outcome measures** (cluster randomized controlled study in Phase 1)**:**<br>(i) % of children cured at day 7* in the intervention group (ePOCT+) as compared to control group (routine care)<br>(ii) % of children prescribed an antibiotic at initial consultation, as reported by HCWs, in the intervention group (ePOCT+) as compared to control group (routine care)<br>* according to caregivers contacted through phone or home visit (non-referred secondary hospitalizations will however be considered as clinical failures even if the child is already cured at day 7)<br>**Primary outcome indicators** (monitored over time and across geographical areas in all HFs in Phase 2)**:**<br>- % of children prescribed an antibiotic at initial consultation<br>- % of children cured at day 7<br>**Secondary outcome measures** (compared between intervention and control HFs in Phase 1) **or indicators** (monitored over time and across geographical areas in all HFs in Phase 2)**:**<br>*Re-attendance visits:*<br>- % of children with ≥1 unscheduled re-attendance visits at any HF by day 7<br>*Severe clinical outcome by day 7:*<br>- % of children with non-referred secondary hospitalization by day 7<br>- % of children who have died by day 7<br>*Primary referrals:*<br>- % of children referred to hospital or admitted to inpatient ward at a health centre at initial consultation<br>- % of children who were hospitalized among those referred (i.e. referral completed)<br>*Appropriate case management for malaria at initial consultation*<br>- % of febrile children tested for malaria<br>- % of malaria positive children prescribed an antimalarial<br>- % of malaria negative children prescribed an antimalarial<br>- % of untested children prescribed an antimalarial<br>*Appropriate case management for other conditions at initial consultation*<br>- % of key symptoms and signs checked and diagnostic tests performed by HCWs<br>- Distribution of final diagnoses (including their severity) made by HCWs<br>- Concordance between treatment prescribed and final diagnoses made by HCWs

	- Concordance between final diagnoses as proposed by ePOCT+ and final diagnoses by HCW *(intervention HFs only)*<br>**Objective 2 (Improve the algorithm and adapt it to spatiotemporal variations):**<br>- Number of positive impact modifications in the algorithm identified by ML analyses<br>- Number of positive impact modifications tested through randomized sub-studies<br>- % of these positive impact modifications confirmed to be safe and useful by randomized sub-studies<br>- Number of modifications implemented, by type of impact (better clinical cure, less severe clinical outcomes, less medicines or diagnostic tests needed, shorter consultation time)<br>**Objective 3 (Enhance disease surveillance and outbreak detection):**<br>- Number of indicators related to disease surveillance and outbreak detection based on individual data that can be visualized through the medAL-outbreak dashboard<br>- Average delay between the date of recording of clinical data and the date they are available on medAL-outbreak<br>- Number and type of epidemic alerts raised by the medAL-outbreak tool<br>- % of these epidemic alerts registered by the district authorities for epidemic response<br>**Objective 4 (Enhance M&E and supportive supervision and mentorship):**<br>- Number of M&E indicators based on individual data that can be visualized through the medAL-monitor dashboard<br>- Number of supervision and mentorship visits to HFs facilitated by the medAL-monitor tool<br>- Number of CHWs reporting having used the medAL-monitor tool for self-auditing their clinical practices<br>- Number of CHWs and members of CHMT reporting a positive experience with the medAL-monitor tool<br>**Objective 5 (Create a supportive environment for the use of clinical algorithms and disease surveillance tools):**<br>- Number of sick children attending primary care facilities managed by HCWs using the electronic ePOCT+ tool<br>- % of CHWs able to assess key clinical signs<br>- Frequency of use of medAL-outbreak by district team members<br>- Frequency of use of the medAL-monitor tool by district team members<br>- Perception by HCWs of the ePOCT+ tool and intervention and the number of improvements they propose for the tool or intervention<br>- Perception by caregivers and community members of the intervention and the number of improvements they propose<br>- Perception by district team members of the medAL-outbreak tool and the number of improvements they propose for the tool<br>- Number of medical experts able to modify a clinical algorithm using MedAL-Creator<br>- Number of computer scientists able to maintain the software and the IT infrastructure for clinical algorithms<br>- Number of scientists able to analyze clinical data using ML<br>- Number of local students trained in applied ML for health care in resource-limited settings<br>- Perception of the current and future utility of ML to improve health care amongst clinical experts (deciding on algorithm changes) and health authorities<br>- Patient and provider expenditures related to care of acutely ill children included in the study

	- Carbon footprint of the intervention or carbon-saving due to the intervention
**Procedures and measurements**	**Objective 1: Improve the integrated management of sick children**<br>In **Phase 1**, in each region, after a pilot period of 6 months to test all the e-tools, eligible HFs will be randomized (by Region and HF) to the intervention or control arm. Sick children will be enrolled over a period of 6 to 9 months.<br>Primary outcome measures: (i) For assessing clinical cure at Day 7, a simple questionnaire (asking if the child is still sick, was hospitalized secondarily, or died) will be administered to all caregivers by phone; (ii) For measuring antibiotics prescribed at initial consultation, the treatment data recorded in the tablets or PCs by HCWs throughout the study will be compared between intervention and control arms.<br>Secondary outcome measures: To determine the quality of the consultation made by CHWs, including the rational use of antimicrobials (antibiotics and antimalarials), two sources of data will be used and compared between intervention and control arms:<br>- the final decision on referral, tests performed, final diagnoses retained and treatment prescribed will be recorded in the tablets or PCs by HCWs at initial consultations and re-attendance visits.<br>- during a cross-sectional survey, observers in the consultation room will record key symptoms and signs assessed by HCWs, diagnostic tests requested, final decision on referral, final diagnoses retained and treatment prescribed.<br>**Control arm:** In control PHC facilities, sick children will be assessed, diagnosed and treated as per routine protocols by clinicians. A similar but simplified tablet or PC application will also be used in control facilities, but only to record outcome measure related data using an electronic case report form. HCWs in control facilities will be offered the usual training and supervision proposed by the CHMT.<br>In **Phase 2**, all selected HFs in the two regions will receive the intervention. Indicators monitored over time and across geographical areas will be measured the same way as in Phase 1, except that clinical cure, secondary hospitalizations and deaths may or may not be assessed through a phone call or an automated SMS or call to caregivers using the same simple questionnaire.<br>**Objective 2: Improve the algorithm and adapt it to spatiotemporal variations**<br>The algorithm will be improved in terms of diagnostic and prognostic accuracy and adapted to local fluctuations in epidemiology, demography, geography and resource availability. These modifications will be designed and supervised by clinical experts, based on accumulated data captured during the study in real-time and analyzed, including by ML when necessary. All modifications will first be tested on retrospectively collected data. If these analyses confirm an expected positive impact, the change in the algorithm will be implemented and its impact will be monitored through the continuous analysis of data looking at the related outcome indicators. For major modifications in the algorithm, a formal randomized sub-study will be conducted to assess precisely the potential benefit (or harm) on clinical outcome or non-inferiority. HCWs attending a child who could potentially benefit from this modification will be informed about the change in the algorithm. Either the modified branch or the standard branch of the algorithm will then appear on the screen to guide the HCW's decisions. Antimicrobial prescriptions, clinical cure and other outcomes related to the expected impact will be assessed the same way as in Phase 1 and compared between children managed with the new and the standard version of ePOCT+. If the positive impact is confirmed by the sub-study, the new algorithm branch will be implemented for all relevant children in the relevant HFs. Minor modifications in the algorithm (e.g. adding a non-critical symptom such as myalgia, or a slight modification of threshold of a laboratory test) will be implemented without sub-study, but some outcome indicators may be monitored.<br>**Objective 3: Enhance disease surveillance and outbreak detection**<br>All data generated by ePOCT+ will be sent to a central sever based at IHI. Data will be visualized through the medAL-outbreak dashboard made available to district health authorities using the key indicators they will have chosen. To enhance disease

	surveillance, targeted microbiological testing will complement the syndromic surveillance data generated automatically by ePOCT+. This microbiological testing will take place during Phases 1 and 2 in 6 to 8 sentinel HFs (3 to 4 per site) – and potentially other HFs with abnormal spatiotemporal patterns in syndromic surveillance data – on children presenting with at least one of three syndromes (significant respiratory disease, gastro-intestinal disease or non-specific fever). Rapid tests will be used on site and appropriate microbiological tests (PCR and serologies) will be performed at the research laboratories of NIMR-Mbeya and IHI-Ifakara/Bagamoyo once every other week. Abnormal patters in the surveillance data identified by ML will also be investigated by performing the relevant laboratory tests to identify a potential outbreak. Results will be discussed with the Epidemiology section under Directorate of Preventive Services (DPS), including the necessity for additional confirmatory tests at the Geneva Centre for Emerging Viral Diseases. The clinical management algorithm will be modified temporarily, if necessary, according to the clinical experts. Additional laboratory analyses are intended to improve surveillance capacity at the district and national levels. We do not anticipate that additional laboratory analyses will be useful in patient management because of the lag time between sample collection and availability of the results. However, if this unlikely scenario presents itself and it is necessary to contact the patient again for additional treatment, it will be possible to retrieve his/her contact information and do so.<br>**Objective 4: Enhance M&E and supportive supervision and mentorship**<br>Patient-level data will also enhance M&E activities and supervision and mentorship of the HCWs through the medAL-outbreak and medAL-monitor platforms. Clinicians will use data generated from their own consultations to self-audit their clinical practices by monitoring quality of care indicators in the Clinivisor dashboards and improve their skills in specific areas using the e-learning platform. Similarly, members of the CHMT will use medAL-monitor to identify and provide support to HFs in need of additional training and mentorship. In time, this enhanced ability to capacitate HCWs and improve quality of care should become evident in the trends in the M&E indicators displayed in medAL-outbreak and subsequently in DHIS2.<br>**Objective 5: Create a supportive environment for the use of clinical decision support algorithms and disease surveillance tools**<br>During Phases 1 and 2, implementation mixed-methods research will be undertaken to assess facilitating factors to ePOCT+ deployment. HCWs, caregivers, community members and district health managers will be interviewed to assess the perception and evaluate the acceptability of ePOCT+ and related tools.<br>Sustainability of the intervention will be evaluated by assessing the level of local human capacities that will have been reached at the end of the study in terms of: 1) clinical competencies of HCWs; 2) clinical algorithm creation by medical experts; 3) software and IT infrastructure maintenance by computer scientists; 4) disease surveillance and outbreak detection by health authorities and; 5) ML analysis by data scientists. These capacities will be enhanced by specific professional training programs and exchanges organized by the different institutions involved in the DYNAMIC project, as well as the University of Dar es Salaam.<br>Finally, cost analyses will be performed using ML on the data collected during Phases 1 and 2, assigning a cost to each procedure performed or medication prescribed during consultations, as well as to the transport to come to the HF or to the hospitalization of the child if any. An ecological assessment of the intervention will also be performed to calculate the carbon footprint or carbon-saving of the intervention.
**Statistical considerations**	Throughout the study, non-inferiority in clinical cure will be defined as a risk difference of <30% in the proportion of clinical failures while superiority in clinical cure will be defined as a decrease of ≥30% in the proportion of clinical failures. Superiority regarding resources (whenever medicines prescribed or diagnostic tests ordered by HCWs or time spent to perform a consultation) will be defined as a decreased need of ≥25%.

	For the cluster-randomized study in Phase 1, the primary analysis will evaluate whether the use of ePOCT+ for case management of sick children in PHC facilities results in 1) decreased antibiotic prescriptions at the initial consultation (superiority analysis) and 2) similar clinical cure at day 7 (non-inferiority analysis), compared to routine care. The sample size was calculated for testing the non-inferiority of the clinical cure outcome, which will require a much larger sample size due to the rareness of the event. In addition, the testing of differences in antibiotic prescription is conditional on satisfying non-inferiority in the clinical outcome. Assuming a cure rate of 97% in the control arm based on previous studies, the acceptable absolute non-inferiority margin will thus be one third of 3% (100%-97%), thus 1%. We assumed a cluster size of 900 patients and an intraclass correlation coefficient of 0.002 in the clinical outcome based on similar studies. To have 80% power to detect this difference of ≥1%, for a one-sided hypothesis test at alpha of 0.025, we would require 19 clusters and 17,100 patients per arm (total patients n=37,620 assuming 10% loss to follow-up), rounded to a total of 40 HFs and 40'000 patients.<br>For Phase 2, sample sizes for the randomized sub-studies, aimed at evaluating the effect of a modification in the algorithm leading to a potential positive impact, will be calculated following the same general principles. In Phase 2, we expect that an average of 200 children per month per HF will be managed using ePOCT+, which corresponds to around 500,000 consultations over 2 years in the 100 HFs selected on predefined criteria.
**Study duration**	Phase 1 (including pilot phase): January 2021 – March 2022<br>Phase 2: April 2022 – October 2023<br>Implementation research: January 2021 – January 2023<br>Sustainability assessment: January 2023 – March 2024<br>Diseases Surveillance: October 2021 – March 2023
**Study schedule**	Cluster-randomized controlled study data collection (Phase 1): Sep 2021 – Mar 2022<br>Data collection for the evaluation of the spatiotemporal/prognostic adaptations of ePOCT+ (Phase 2): Apr 2022 – Oct 2023<br>Data collection for the evaluation of the smart disease surveillance and outbreak detection system: Oct 2021 – Mar 2023<br>Data collection for the cross-sectional mixed-methods studies: Jan – Mar 2021; Jan – Mar 2022; Jan – Mar 2023<br>Final data analysis of phase 2 and of the contextual evidence that drives sustainability and future scale up: Jan 2023 – Mar 2024
**Ethical statement**	The research project will be carried out in accordance with the research plan outlined in this protocol and with principles enunciated in the current version of the Declaration of Helsinki, Essentials of Good Epidemiological Practice issued by Public Health Switzerland (EGEP) as well as all national legal and regulatory requirements as applicable.
**Relevant references**	• Cordey S, Laubscher F, Hartley M-A, Junier T, Pérez-Rodriguez FJ, Keitel K, Vieille G, Samaka J, Mlaganile T, Kagoro F, Boillat-Blanco N, Mbarack Z, Docquier M, Tapparel C, Kaiser K , D'Acremont V. Detection of Dicistroviruses RNA in Blood of Febrile Tanzanian Children. *Emerg Microbes Infect*. 2019, 8(1):613-623.<br>• Cordey S, Hartley MA, Keitel K, Laubscher F, Brito F, Junier T, Kagoro F, Samaka J, Masimba J, Said Z, Temba H, Mlaganile T, Docquier M, Fellay J, Kaiser L, D'Acremont V. Detection of novel astroviruses MLB1 and MLB2 in the sera of febrile Tanzanian children. *Emerg Microbes Infect*. 2018; 7(1):27.<br>• D'Acremont V, Kilowoko M, Kyungu E, Philipina S, Sangu W, Kahama-Maro J, Lengeler C, Cherpillod P, Kaiser L, and Genton B.'Beyond Malaria — Causes of Fever in Outpatient Tanzanian Children', *NEJM* 2014; 370: 809-17.<br>• De Santis O, Kilowoko M, Kyungu E, Sangu W, Cherpillod P, Kaiser L, Genton B, D'Acremont V. Predictive value of clinical and laboratory features for the main febrile diseases in children living in Tanzania: A prospective observational study. *PLoS One* 2017;12(5):e0173314.

	• Erdman LK, D'Acremont V, Hayford K, Rajwans N, Kilowoko M, Kyungu E, Hongoa P, Alamo L, Streiner DL, Genton B, Kain KC. Biomarkers of Host Response Predict Primary End-Point Radiological Pneumonia in Tanzanian Children with Clinical Pneumonia: A Prospective Cohort Study. *PLoS One* 2015; 10(9): e0137592.<br>• Keitel K, Kilowoko M, Kyungu E, Genton B, D'Acremont V. Performance of prediction rules and guidelines in detecting serious bacterial infections among Tanzanian febrile children. *BMC Infect Dis* 2019; 19:769.<br>• Keitel K, Samaka J, Masimba J, Temba H, Said Z, Kagoro F, Mlaganile T, Sangu W, Genton B, D'Acremont V. Safety and efficacy of C-reactive protein-guided antibiotic use to treat acute respiratory infections in Tanzanian children: a planned subgroup analysis of a randomized, controlled non-inferiority trial evaluating a novel electronic clinical decision algorithm (ePOCT). *Clin Infect Dis.* 2019; 69(11):1926-1934.<br>• Keitel K, D'Acremont V. Electronic Clinical Decision Algorithms for the integrated primary care management of febrile children in low resource settings - Review of existing tools. *Clin Microbiol Infect*. 2018, 24(8):845-855.<br>• Keitel K, Kagoro F, Samaka J, Masimba J, Said Z, Temba H, Mlaganile T, Sangu W, Rambaud-Althaus C, Gervaix A, Genton B, D'Acremont V. A novel electronic algorithm using host biomarker point-of-care-tests for the management of febrile illnesses in Tanzanian children (e-POCT): A randomized, controlled non-inferiority trial. *Plos Medicine* 2017;14(10):e1002411.<br>• Rambaud-Althaus C, Shao S, Samaka S, Swai N, Perri S, Kahama-Maro J, Mitchell M, D'Acremont V, and Genton B. 'Performance of Health Workers Using an Electronic Algorithm for the Management of Childhood Illness in Tanzania: A Pilot Implementation Study.' *Am J Trop Med Hyg* 2017; 96: 249-57.<br>• Shao AF, Rambaud-Althaus C, Samaka J, Faustine AF, Perri-Moore S, Swai N, Kahama-Maro J, Mitchell M, Genton B, D'Acremont V. New Algorithm for Managing Childhood Illness Using Mobile Technology (ALMANACH): A Controlled Non-Inferiority Study on Clinical Outcome and Antibiotic Use in Tanzania. *PLoS One* 2015; 10(7): e0132316.<br>• Shao A, Rambaud-Althaus C, Swai N, Kahama-Maro J, Genton B, D'Acremont V, and Pfeiffer C. 'Can smartphones and tablets improve the management of childhood illness in Tanzania? A qualitative study from a primary health care worker's perspective.', *BMC Health Services Research* 2015; 15: 135.

TABLE OF CONTENTS

1 Background and rationale 18
1.1 Managing sick children at the primary care level 18
1.2 Antimicrobial resistance at individual and population levels 18
1.3 Training, supervision and self-auditing at health facility level 19
1.4 Monitoring and evaluation of health interventions 19
1.5 Disease surveillance and epidemic detection 19
1.6 Clinical decision support algorithms and results so far achieved in Tanzania 20
1.7 Health system, socioeconomic and ecological impact 22
2 Study objectives and theory of change 23
2.1 Objective 1 23
2.2 Objective 2 24
2.3 Objective 3 24
2.4 Objective 4 24
2.5 Objective 5 24
2.6 Theory of change 24
3 Study design 25
3.1 Phase 1: cluster randomized controlled study in 40 health facilities 25
3.2 Phase 2: scale-up of the intervention to 100 health facilities and transformation into a dynamic algorithm 26
3.3 Syndromic surveillance enhanced by microbiological testing 27
3.4 Mixed-methods operational research 28
3.4.1 Cross-sectional quality of care surveys 28
3.4.2 In-depth interviews, focus group discussions and surveys with health care workers 28
3.4.3 Caregiver and patient exit interviews 29
3.4.4 Focus group discussions with selected community groups 29
3.4.5 In-depth interviews with members of the CHMTs 29
3.4.6 Economic and ecological evaluations 29
4 Study area 30
4.1 Study sites 30
4.2 Selection of health facilities 32
5 Study population 32
5.1 Sick children attending PHC facilities 32
5.2 Other stakeholders and/or beneficiaries 33
6 Description of the intervention 33

6.1 Management of sick children by health care workers using ePOCT+ 34
6.2 Regular update of ePOCT+ to improve accuracy and adapt to epidemiological changes 36
6.3 Initial in-person training and e-leaning modules available on tablets 37
6.4 Supervision of health facilities by the CHMT using medAL-monitor 37
6.5 Disease surveillance and outbreak detection by district health authorities using medAL-outbreak 37
7 Outcome measures and indicators 40
7.1 Outcome measures and indicators for Objective 1 (Improve the integrated management of sick children) 40
7.2 Indicators for Objective 2: Improve the algorithm and adapt it to spatiotemporal variations 42
7.3 Indicators for Objective 3: Enhance disease surveillance and outbreak detection 42
7.4 Indicators for Objective 4: Enhance M&E and supportive supervision and mentorship 42
7.5 Indicators for Objective 5: Create a supportive environment for the use of clinical algorithms and disease surveillance tools 42
8 Study procedures 43
8.1 Objective 1: Improve the integrated management of sick children 43
8.1.1 Recruitment, screening and informed consent procedure 43
8.1.2 Management of sick children during the initial consultation 44
8.1.3 Re-attendance visits in case of persisting or worsening of symptoms 46
8.1.4 Training of health providers on how to use ePOCT+ 46
8.1.5 Supervision of health providers by the CHMT 46
8.1.6 Type of care, training and supervision provided at intervention and control facilities 47
8.1.7 Measurement of primary and secondary outcomes 47
8.1.8 Unique study identification number for each participant 48
8.2 Objective 2: Adapt the algorithm to epidemiological and demographic conditions 49
8.3 Objective 3: Enhance disease surveillance and outbreak detection 50
8.3.1 Training and feedback of the CHMT on medAL-outbreak 51
8.4 Objective 4: Enhance M&E and supportive supervision and mentorship 51
8.5 Objective 5: Create a supportive environment for the use of clinical decision support algorithms and disease surveillance tools 52
8.5.1 Implementation research 52
8.5.2 Stakeholder involvement and community sensitization 52
8.5.3 Development of frameworks for the future scale-up of the e-POCT+ intervention 52
9 Laboratory procedures 53
9.1 Specimen collection and management 53

9.2 Serology to characterize exposure to viral and/or bacterial pathogens 54
9.3 Viral detection and discovery 54
9.4 Molecular detection of bacterial pathogens 54

10 Statistical methods 54

10.1 Sample size calculations 54
10.1.1 Cluster-randomized controlled study (Phase 1) 55
10.1.2 Randomized sub-studies to test algorithm modifications (Phase 2) 55
10.2 Estimated number of beneficiaries 55
10.3 Statistical analyses for the primary outcome measures in the cluster randomized controlled study of Phase 1 56
10.4 Machine learning analyses 56
10.4.1 Supervised classification and correlation 57
10.4.2 Anomaly detection 57
10.5 Analyses of data from operational research studies 57

11 Information technology, data management and monitoring 57

11.1 Overall IT solution 57
11.2 Data flow 58
11.3 Data security and privacy 59
11.4 Data quality monitoring 60
11.5 Monitoring of study procedures and major health system barriers 60

12 Ethical considerations 61

12.1 Participant information and consent 61
12.2 Risk-benefit assessment 61
12.3 Premature termination of study 63
12.4 Local regulations, declaration of Helsinki and protocol amendments 63
12.5 Alignment of the project with regional and/or governmental strategies 63

13 Organization and timelines 63

13.1 Organizational structure 63
13.2 Roles of investigators and collaborators 64
13.3 Timelines 67

14 Capacity building, knowledge transfer and dissemination plans 68

15 Budget 69

16 References 71

17 Appendices 74

1 BACKGROUND AND RATIONALE

1.1 Managing sick children at the primary care level

Children are a well-recognized vulnerable population that still suffers from a high rate of acute infectious diseases and preventable deaths due to inadequate management of children at primary health care level. This is especially true in the fragile health systems of Sub-Saharan Africa, where under-five mortality is 10 times higher than in high-income countries. In these settings, health professionals lack appropriate diagnostic tools and skills. Outpatient providers are supposed to rely on disease management guidelines, such as the WHO IMCI booklet (WHO 2014A). The IMCI strategy, with its integrative disease management approach, where several health problems are considered in the same consultation, remains very relevant today. Yet its implementation has faced major challenges, largely related to format and content-related shortcomings (Rambaud-Althaus et al. 2017; Lange, Mwisongo, and Mæstad 2014; Baiden et al. 2011). For example, the IMCI algorithm is over-reliant on clinical signs and symptoms; these inherently lack diagnostic accuracy in children and expend much of the short timeframe allocated for consultations (Keitel et al. 2017; Rambaud-Althaus et al. 2015). Diagnostic POC tests that can help detect children with severe illness or those in need of antibiotic treatment have not been integrated into the IMCI algorithm. The IMCI booklet asks for integration of information from about 20 pages during a consultation, which is a great challenge for HCWs. Practice guidelines like IMCI also become rapidly outdated as they are static in nature. With revision cycles of 5-10 years, guidelines cannot keep up with updated evidence and epidemiological changes. Existing guidelines are also too generic in that only a single version exists for an entire country, while each region faces different types of diseases and levels of transmission. When an epidemic arises, such static and generic guidelines can even become dangerous: existing recommendations may become inapplicable with changing disease patterns. As a result, HCWs may put standard guidelines aside to focus on the disease outbreak. For example, the risk of malaria deaths increased during the West African Ebola epidemic as HCWs focused on Ebola (Walker et al. 2015).

Another important component of pediatric acute care is patient triage. This ensures that patients with problems that require urgent attention are prioritized in the queue. Triage at peripheral HFs is practically nonexistent (Hansoti et al. 2017). Patients are seen in the order of arrival; at risk patients, such as newborns, may rapidly deteriorate while waiting to be seen. The installation of simple registration and triage tools would also allow improving the overall workflow at facilities.

1.2 Antimicrobial resistance at individual and population levels

In the absence of diagnostic tools, clear guidance, and supervision, HCWs tend to over-prescribe antimicrobials (Hopkins et al. 2017, Fink et al. 2019). Out of ten febrile children attending primary care facilities, only about one or two children needs antibiotic treatment (D'Acremont et al. 2014), but nine receive antibiotics. This is because, without diagnostic tools, it is challenging for HCWs to safely identify the minority of children with bacterial infections among the predominant cases of self-limiting diseases (Rambaud-Althaus et al. 2017). Thus, HCWs tend to prescribe antibiotics - and sometimes also antimalarials - in the vast majority of cases “to be on the safe side”. Despite this high-volume and indiscriminate antibiotic treatment, mortality from childhood infections remains high. This is mainly because children with severe infections, which can be caused by viral, parasitic, or bacterial pathogens, are missed (Keitel et al. 2017). The over-reliance on antibiotics has resulted in the neglect of other important supportive treatments for such severe infections (e.g. oxygen therapy for viral bronchiolitis). Antibiotic over-treatment also hinders the development of the natural, protective bacterial gut flora, which is an important immunological barrier (Benoun et al. 2016). The tremendous overuse of antibiotics, especially in children at the primary care level where most patient encounters occur, contributes to the global spread of antibiotic resistance, which is a major public health threat, estimated to be responsible for up to 10 million deaths per year by 2050 (Holmes et al. 2016, Fink et al. 2019). According to the WHO, antibiotic resistance is rising to dangerously high levels, especially

in low-resource settings. To meet the urgent need to mitigate AMR, including strategies for rationalizing and monitoring antibiotic prescriptions, Tanzania has developed a National Action Plan for AMR 2017-2022. In this plan, the need for antimicrobial stewardship programs for prescribers is included in the Priority action n°9.

1.3 Training, supervision and self-auditing at health facility level

Current standards of training and supervision for HCWs have much potential for further improvement. Continuous education still largely consists of centrally organized, generic refresher trainings, which often lack relevance to the individual HCW (Kiplagat et al. 2014) and are generally considered less effective than on-the-job training (WHO, 2006). Flexible, mobile solutions for continuous education at the HF level have not been implemented. Supervision personnel are often located at district level, far away from the HCWs. Due to logistical challenges, supervision visits can only happen infrequently and lack efficacy and efficiency. This is largely because the district supervision teams do not possess data that could inform them about problems and needs at the point of care. Personalized, data-driven feedback for front-line providers does not exist to date.

1.4 Monitoring and evaluation of health interventions

Currently the planning, budget allocation, monitoring, and evaluation of health interventions is largely based on reports of data aggregated at the facility level, called MTUHA in Tanzania, and managed in the DHIS2 platform used by the majority of African countries. Data aggregation at the facility is done manually on paper forms or excel tables using paper records that are supposed to be filled by HF personnel on a daily basis. Since the filling of these registries is additional work on top of patient care, registries are often incomplete and may be filled in retrospectively (Wilms et al. 2014). Even if HCWs are motivated to report correct data, they lack the tools to make correct diagnoses. As a result, prevalence of many conditions that require diagnostic tools that are often out of stock or not available altogether, is falsely represented in DHIS2. For example, bacterial pneumonia is constantly over-diagnosed as diagnostic tests to distinguish bacterial from viral respiratory infections are not available. Furthermore, the manual data aggregation at the facility level and entry process into DHIS2 is error prone. In sum, the data available for health management and reporting is of insufficient quality (Mghamba et al. 2004, Farnham et al. 2020).

Misdiagnoses have consequences that reach beyond the patient. They increase re-attendance rates, further congesting PHC clinics and accruing economic losses not only for families but for the entire health system. Systematic errors in patient-level data accumulate, and as they are aggregated in DHIS2 to measure population-level indicators, they have the potential to bias the statistics used to prioritize public health interventions and, importantly, identify epidemics.

1.5 Disease surveillance and epidemic detection

The epidemiology of diseases and the number and intensity of epidemics are expected to change even more than before, due to the rapid urbanization and climate change (WHO, 2019). The later will in particular increase epidemics caused by arboviruses, as they are transmitted by mosquitoes whose optimal reproductive temperature is 30°C, a level that Tanzania (presently experiencing an average temperature of 26°C) will become closer to in the coming years. It will also shift transmission of malaria from West Africa, where it will become too hot for anopheles to reproduce, to East Africa (Ryan et al. 2015) The altered rainfall patterns due will also cause more floods, thus increasing the risk of waterborne and water-related diseases such as cholera, typhoid and leptospirosis.

Since the true burden of disease is measured neither precisely nor timely, disease surveillance is inaccurate and epidemics are detected too late. This results in a distorted prioritization of health interventions. For example, at the beginning of the Ebola crisis, the increase in diarrheal cases in Gueckedou were identified late and attributed incorrectly to a cholera epidemic, which caused the Ebola virus to spread further (WHO, 2014B). Outside of outbreak scenarios, viral respiratory infections

are commonly misclassified as bacterial pneumonia. Health authorities then focus on the adequate supply of antibiotics, rather than on strengthening the medical treatment for severe viral respiratory infections (bronchiolitis and asthma), such as oxygen or bronchodilators (Nantanda et al. 2013). Due to the slow manual health data aggregation process and transfer into DHIS2, epidemic response is delayed, and diseases may be left uncontained.

Disease surveillance should normally include laboratory data (i.e. microbiological tests). In the absence of laboratory tests, syndromic surveillance (as opposed to microbiological surveillance), where information only on signs and symptoms of patients are collected. Syndromic surveillance can be helpful if data quality is high (which is often not the case as described above), but is generally insufficient due to inability to confirm the causal pathogen. For example, unspecific fever could be caused by parasites (e.g. malaria), viruses (e.g. dengue) or bacteria (e.g. leptospirosis), all requiring very different prevention and control measures. For surveillance, a higher number of diagnostic tests (for example, detecting arboviruses) is thus required, as compared to the few tests that are useful for the daily management of patients at primary care level. On the other hand, untargeted microbiological surveillance (testing many samples for many different pathogens) is unspecific and very expensive. Therefore, microbiological tests should be applied in a few sentinel sites and sub-groups of patients with a specific clinical presentation following a predefined strategy. Unfortunately, in the primary HFs, the tools to inform targeting of microbiological testing to cases with the highest potential to contribute to outbreak detection are not available.

1.6 Clinical decision support algorithms and results so far achieved in Tanzania

The WHO has identified digital health interventions and predictive tools in primary care as key accelerators in achieving the 2030 Sustainable Development Goal 3 of ensuring good health and well-being for all. New simple and cheap technologies, such as mobile devices, coupled with the advances in computing and data science, could help mitigate several of the challenges described in sections 1.1-1.5.

Thus, Unisanté, Swiss TPH and IHI have developed and clinically validated smartphone-based electronic CDSAs for the management of sick children since 2010 (building on experience in developing algorithms for returning sick travelers and migrants in Switzerland since 2005). Two versions of the algorithm have been developed and evaluated in controlled research conditions, as summarized below. These electronic, interactive tools have the advantage over paper-based guidelines in that they can integrate sophisticated algorithms (which are necessary to accurately assess a sick child) while maintaining simple, user-friendly interfaces and teaching features.

The first-generation algorithm for the management of childhood illness (ALMANACH) was trialed in Tanzania in 2010-2011, achieving improved clinical cure (from 92% to 97%) and a decrease in antibiotic prescription (from 84% to 15%) as compared to routine care (Shao et al. 2015A). ALMANACH also led to more consistent clinical assessments without taking more time than a conventional consultation and was perceived by clinicians as “a powerful and useful” tool (Shao et al. 2015B).

The second-generation algorithm called ePOCT, ‘electronic Point of Care Tests’, was based on a thorough review of the evidence and the existing national and international guidelines on the diagnosis and management of sick children (Keitel et al. 2017). In addition to symptoms and signs, it made use of several POC tests to help detect children with severe infections requiring hospital-based treatment and/or children with serious bacterial infection. The interactive electronic decision tree supported a systematic assessment of the child and the consideration of several health problems at once (**Figure 1**). It increased the ability to detect children with severe disease, which was further supported by pulse-oximetry and POC hemoglobin testing (to screen for severe anemia, a strong predictor of bacteremia). POC inflammatory host biomarkers, such as CRP, detected children in need for antibiotic treatment. Diagnostic and triage tests were only performed in patient groups with sufficient pre-test disease probability (which is indicated by the algorithm). Integrating

such POCTs is important since clinical signs alone lack accuracy in detecting children with severe infection and/or in need for antibiotic treatment.

Figure 1: ePOCT addresses the major challenges in pediatric case management.

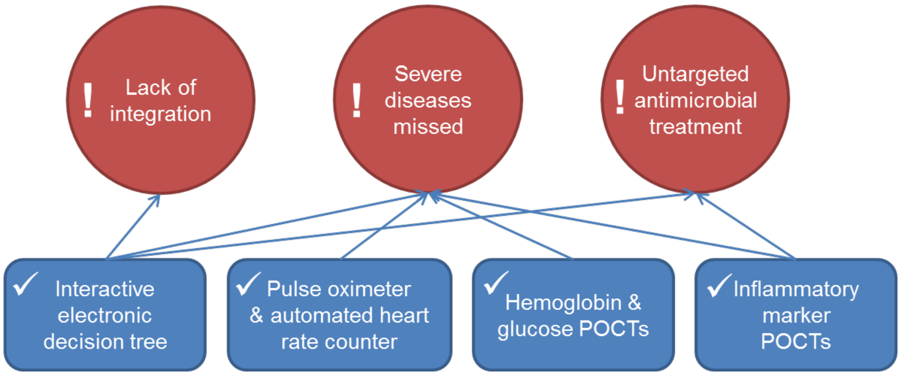

The safety of ePOCT was assessed among over 3,000 febrile children aged 2 months to 5 years through a randomized trial in Dar es Salaam, Tanzania in 2014-2016 (Keitel et al. 2017); this was an essential first step since ePOCT contained important upgrades compared to IMCI. The use of ePOCT resulted in higher clinical cure (98%) as compared to ALMANACH (96%) and routine care (95%). The algorithm also further reduced antibiotic prescription to 11%, as compared to 30% with the use of ALMANACH and 95% in routine care (**Figure 2**).

Figure 2. Percentage of antibiotic prescription for children under-five with fever when managed per e-POCT and ALMANACH algorithms, and per routine care in Dar es salaam, Tanzania.

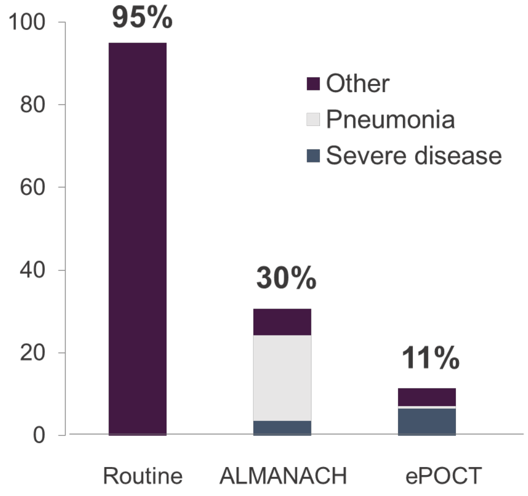

The great potential of ePOCT to improve care now calls for further validation and implementation studies in different countries and epidemiological settings, and in less controlled conditions, as a basis for further scale-up (**Figure 3**). The content of ePOCT should also be extended from febrile children aged 2 months to 5 years to the entire pediatric age range and clinical spectrum so that the clinicians can use the tool for all of their consultations with children. The development of a young infant (0 to 2 months) component will be especially important, as this age-group is at highest risk of mortality.

Figure 3: The validation cycle for clinical decision support algorithms (adapted from Keitel et al. 2018)

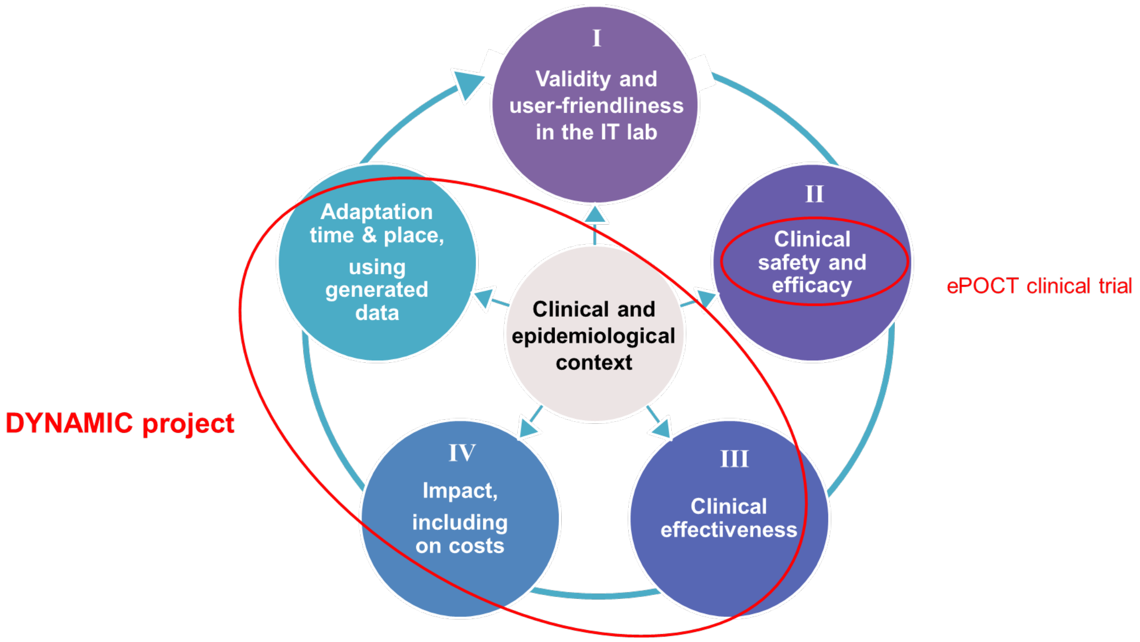

1.7 Health system, socioeconomic and ecological impact

The ongoing digitalization of the health sector bears great potential for improving delivery and quality of care and for using real-time data in public health decision making. However, it also poses certain risks that must be mitigated by putting in place appropriate strategies to ensure patient confidentiality and data security, to avoid fragmentation and duplication of efforts, and to provide transparency within the health system to streamline this process. Furthermore, systems today need to be integrated, sustainable, flexible and scalable, while being mindful of their carbon footprint. These features of technological innovations in the health sector have increased the need for national frameworks for governance and guidelines on e-health. The DYNAMIC project aims at integrating innovative cutting-edge technologies and methodologies to improve quality of care in PHC settings in Tanzania and to provide the health system with innovative tools to improve disease surveillance and enable early detection of epidemics. Furthermore, it aims to contribute to the development of national frameworks and guidelines for the scale-up of the intervention, while bearing in mind potential socioeconomic and ecological impacts.

ePOCT+ should better identify children with severe disease who require immediate referral, treatment and supportive care. Early and appropriate triage and management of sick children not only leads to reduced mortality and morbidity but also decreases their exposure to unnecessary antimicrobials, which protects their natural gut flora that has recently shown to be essential for the development of their immunity. Rational use of antimalarials and antibiotics would result in a reduction of the drug pressure in the community and AMR, and eventually a reduction in morbidity, mortality and health care costs due to antimicrobial resistant infections. A cost analysis will be included in the project in order to estimate costs to the patient, provider, and health care system.

ePOCT+ is designed to promote social cohesion between patients and HCWs since it presents structured consultation steps which encourage dialogue and physical examination (Bessat et al. 2019). It saves time on trying to establish the right diagnosis, allowing clinicians to focus on social communication and fostering confidence through the perceived efficacy and reduced waiting times. Because ePOCT+ is designed to provide personalized treatment algorithms, the clinician is then

made more aware of the individual characteristics of the patient in her/his environment. The project also aims to improve the working relationships between the HCWs and their supervisors by facilitating supportive supervision and mentorship activities.

More accurate reporting of morbidity episodes through a computerized surveillance system would allow to monitor more precisely important child health indicators. This will be especially important in the coming years where the risk of malnutrition in children might increase in the context of a decreased crop yield due to climate change.

As the implementation of digital health interventions is on the rise, it is important to be mindful of their carbon footprint and potential negative ecological effects. Therefore, it is important to evaluate the environmental impact of such projects and think about ways to mitigate it prior to planning for scale-up. The Life Cycle Initiative, a joint project between UNEP and SETAC, has developed a widely accepted framework for environmental assessment methodology. The damage-oriented methods consider "*the cause-effect chain up to the environmental damages, the damages to human health, to the natural environment and to natural resources. These may be expressed for example in additional cases of human health impairment or species endangerment.*" (Jolliet et al. 2004). Damage categories are considered to represent "*the different areas of protection, human health, natural environment, natural resources and man-made environment. These areas of protection, also called safeguard subjects, represent operational groups of subjects (humans, biotic, abiotic and built environment) of direct value to human society. The damage categories group damages to these areas of protection"*. Human health damage category considers in an aggregated and disaggregated manner all kind of impacts that could influence human health in general. The damage indicator, the DALY (Disability Adjusted Life Years), is the common indicator for all these impact categories. Other damage categories are important for the global environment and the current political agenda, like carbon footprint.

More precisely, the impact of the DYNAMIC project will be evaluated using a combination of an environmental impact analysis, cost-effectiveness analysis, and health impact analysis. The LCA methodology will be used, enabling the evaluation of several impacts, as manufacturing, maintenance and end of life of tablets and PCs, and how these impacts can be reduced if these devices are used for other purposes, such as the electronic medical record and HF management systems that health authorities will deploy at the PHC level. Possible improvement of electricity consumption of the tablets and PCs and cooling of the central servers will be assessed.

2 STUDY OBJECTIVES AND THEORY OF CHANGE

The **goal** of this study is to decrease morbidity and mortality of children and mitigate AMR using novel dynamic POC technologies that help front-line HCWs manage sick patients, enhanced by a smart disease surveillance and outbreak detection mechanisms. More specifically, this study seeks to:

2.1 Objective 1

Improve the integrated management of children with an acute illness through the provision of an electronic CDSA (ePOCT+) to clinicians working at primary care level.

Hypothesis 1.1: In routine conditions, sick children attending PHC facilities in Tanzania can be prescribed antimicrobials in a more rational way while having a good clinical outcome, if HCWs are provided with an electronic CDSA on tablets or PCs.

Hypothesis 1.2: The use of ePOCT+ in the outpatient PHC setting in Tanzania will result in improved quality of care.

Hypothesis 1.3: E-learning modules incorporated into the digital tools will improve the HCWs' competence and confidence in managing sick children, as compared to one-off IMCI refresher trainings.

2.2 Objective 2

Improve the accuracy of the **clinical algorithm** and allow it to **adapt to spatiotemporal variations in epidemiology and resources**, based on the data generated through the ePOCT+ tool, analyzed using machine learning and checked by clinical experts.

Hypothesis 2.1: The electronic CDSA can be modified over time using the data gathered through its use by HCWs, and aided by ML-based analyses, in order to improve its accuracy and adapt it to geographical and temporal changes in the disease epidemiology (e.g. outbreaks) or in the availability of resources (e.g. rapid tests).

2.3 Objective 3

Enhance the district (and national) **disease surveillance and outbreak detection capability** using the clinical data generated by the ePOCT+ tool complemented by targeted microbiological investigations and machine learning pattern detection.

Hypothesis 3.1 The generated individual patient level clinical data, complemented by targeted microbiological testing in a few sentinel sites, visualized and analyzed in near real-time can also allow district health authorities to improve disease surveillance and outbreak detection.

2.4 Objective 4

Enhance the district (and national) **health management information system for monitoring and evaluation** and **conducting supportive supervision and mentorship** in HFs using the clinical data generated by the ePOCT+ tool enhanced by additional data analysis and visualization dashboards.

Hypothesis 4.1: The generated individual patient level clinical data that is visualized and analyzed in near real-time will improve the ability of the CHMT to monitor the quality of patient care and undertake targeted supportive supervision and mentorship of HFs and HCWs.

2.5 Objective 5

Create a framework for the development and implementation of **dynamic clinical decision support algorithms** and **disease surveillance tools**, for large-scale, sustainable, and clinically responsible use of machine learning and data science.

Hypothesis 5.1: The interdisciplinary nature of the project, with an emphasis on cross-sectoral collaboration and knowledge/skills transfer, will enable the development of an evidence-based framework for sustainable scale-up of electronic CDSA interventions.

Hypothesis 5.2: The patient level clinical data, when combined with other available spatiotemporal data sources will contribute to a better understanding of the epidemiological and public health profile that could serve as a basis for fostering cross-sectoral collaboration on the issues of health in the intervention districts and beyond.

2.6 Theory of change

The goal and the objectives of the project are based on the following theory of change (**Figure 4**).

Figure 4: DYNAMIC project theory of change

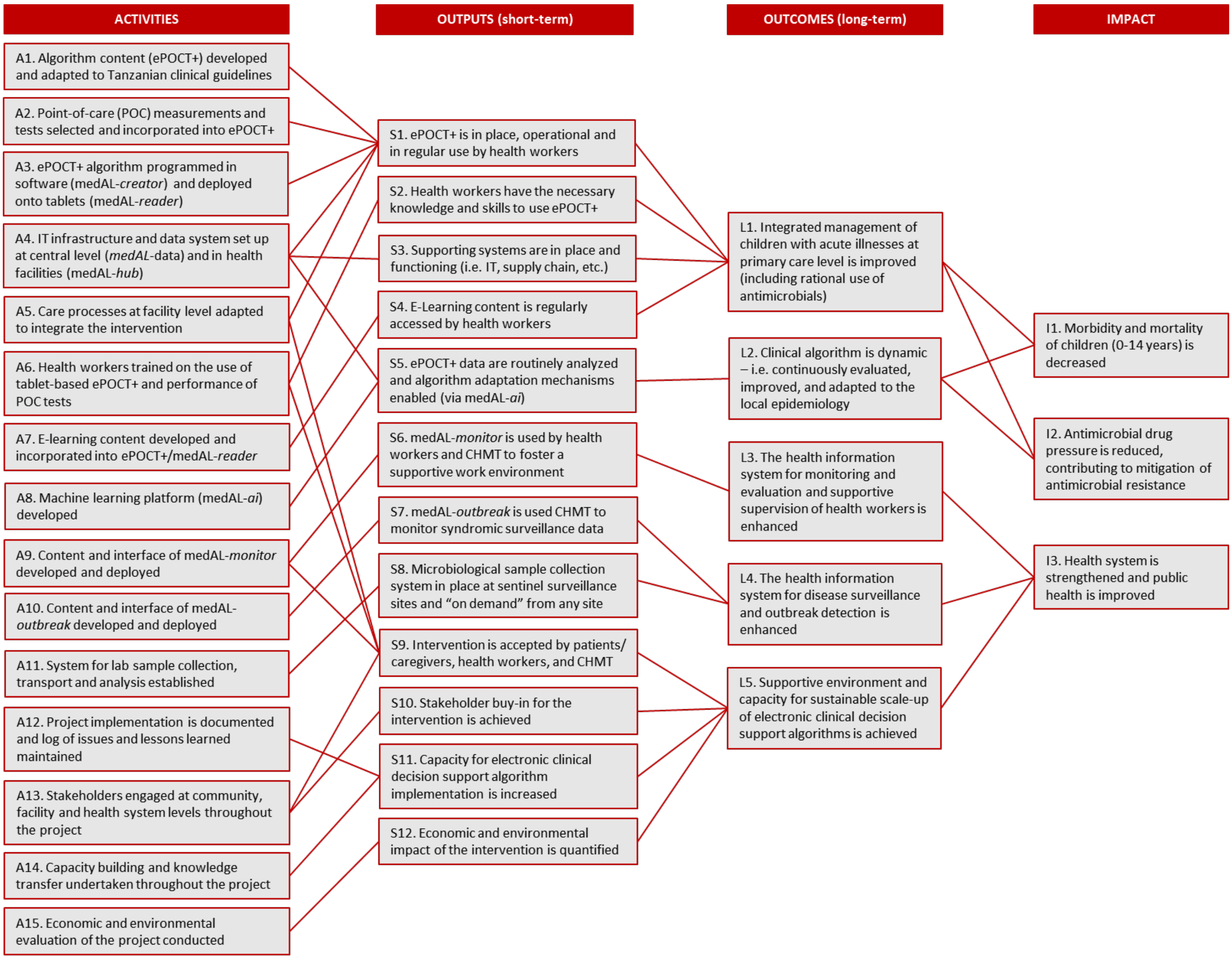

3 STUDY DESIGN

The primary intervention (i.e. ePOCT+ implementation) study will be conducted in two phases, with the study design detailed below and summarized in **Figure 5**.

3.1 Phase 1: cluster randomized controlled study in 40 health facilities

The ePOCT+ tool, as well as the complementary training and supervision tools, will be provided to HCWs of 20 of the 40 selected PHC facilities (intervention arm) in Morogoro and Mbeya regions, while the other 20 HFs will serve as controls (control arm), using a cluster randomized controlled study design. Randomization (1:1) of HFs will be done by region and HF type. Sick children aged 1 day to 14 years will be managed in accordance with the study arm to which the HF they attend has been assigned. The comparator for phase 1 will be routine care as currently implemented in Tanzania (see Section 8.1.5 for more detail). To assess the primary and secondary outcomes, final decision on referral, diagnoses and treatments at initial consultation will be recorded by HCWs electronically; children will also be followed-up by a phone call to their caregiver at day 7 to determine their final health status. Due to the pragmatic nature of the study, the design is adaptive in nature, where changes to the implementation may be incorporated throughout the study to account for issues

encountered in monitoring data and feedback from the field. These changes apply to the implementation context and exclude significant adaptations of the algorithm.

The purpose of Phase 1 is to compare the effect of using ePOCT+ with routine case management of sick children on the rational prescription of antibiotics, while ensuring a favorable clinical outcome. To date, a prior version of the ePOCT tool was assessed in conditions closer to that of an explanatory trial (Keitel et al., 2017). The present study is designed as a pragmatic randomized controlled study to assess the safety and impact on antibiotic prescriptions of implementing ePOCT+ (including its associated POC tests), supported by initial and continuous training and targeted supervision of health workers, in children attending Tanzanian primary health care facilities. The study will be implemented in settings of targeted future use and involving routine health care personnel, and include all sick children (except those coming for a follow-up visit in the context of a chronic condition). ePOCT+ provides support to HCWs in managing these sick children. Since the intervention takes place at the health provider level and that their practices are influenced by the context of the HF they are working in, randomization at HF level rather than at HCW level or at individual patient level was chosen.

3.2 Phase 2: scale-up of the intervention to 100 health facilities and transformation into a dynamic algorithm

The ePOCT+ tool will be extended to the 20 HFs serving as controls in Phase 1, as well as up to 60 additional neighboring facilities of our target area, to reach a total of up to 100 facilities (around 60 in Morogoro and 40 in Mbeya). In Phase 2, an adaptive study design will be used measuring the same outcome indicators as in Phase 1. The medical content of the algorithm will no longer be static, but rather modifiable according to the local data captured by ePOCT+ during Phase 1. Each modification will be designed and evaluated by the clinical expert group for its coherence, safety and potential benefit. Modifications in the algorithms designed to perform as well as the original algorithm but using fewer/different resources will be implemented in the facilities they were designed to serve (for example, if certain facilities do not have access to a certain resource). Similarly, modifications designed to personalize the algorithm according to demographic characteristics, geography or season will be implemented on the target population for which they were intended. Modifications of ePOCT+ aimed at improving health outcomes of subgroups of patients (for example those with respiratory infections) will be implemented in a sub-study where patients are randomized at an individual level. Modifications confirmed as safe and useful will then be implemented, while the non-beneficial changes will not be retained.

Examples of algorithm modifications expected to have positive impacts that will be explored for implementation are: 1) adapting the frequency of malaria testing in areas with very low endemicity; 2) improving the accuracy of pneumonia diagnosis, including , including through the use of novel POC diagnostic tools; 3) assessing the utility of using dengue rapid tests if an outbreak occurs; 4) or typhoid rapid tests in areas of high prevalence; 5) improving the prediction of severe disease; 6) improving the algorithm for malnourished or HIV positive children and; 7) testing sick children for sickle cell disease.

Figure 5: Study design overview.

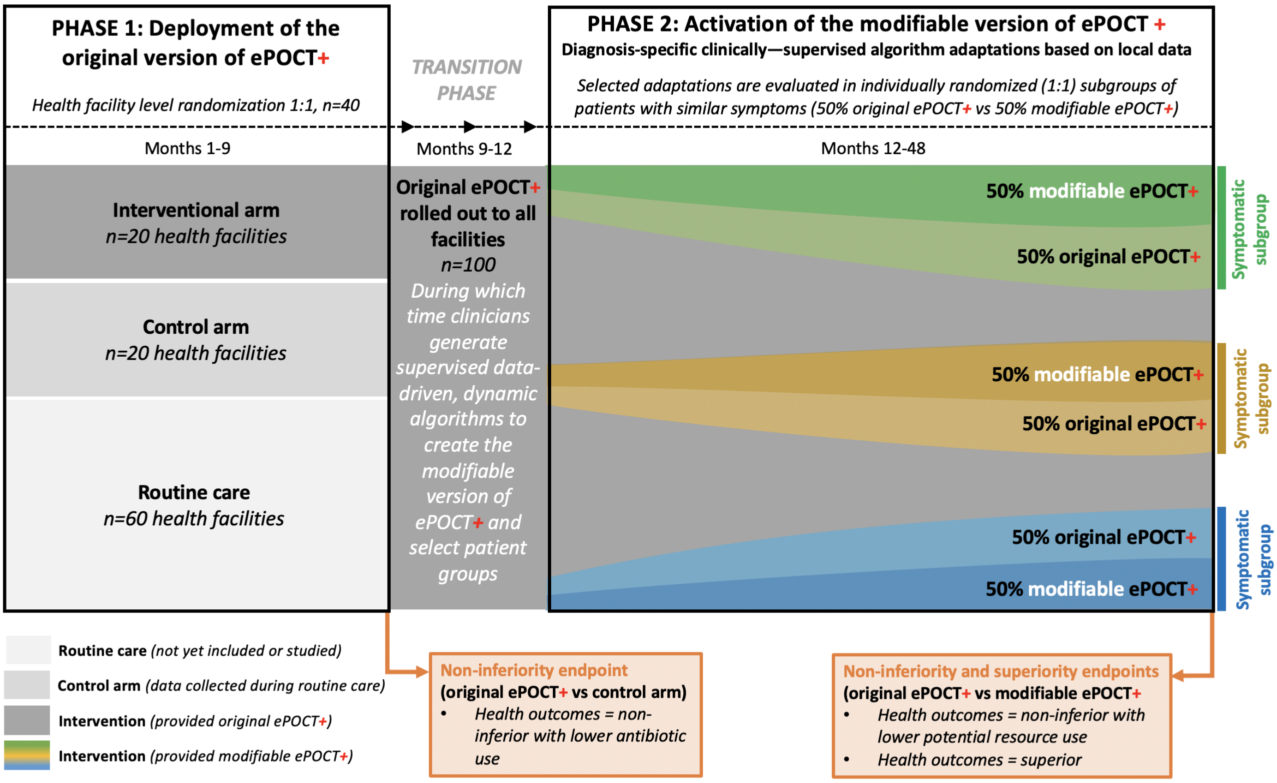

3.3 Syndromic surveillance enhanced by microbiological testing

The syndromic surveillance (i.e. based on the clinical data generated by the use of ePOCT+) will be enhanced by microbiological surveillance in a few sentinel HFs, as well as in any facility according to abnormal data patterns detected by ML (based on predefined clinically supervised thresholds) to identify potential outbreaks or endemic diseases requiring specific attention.

Three to four HFs in each study district (6 to 8 in total) will be selected to play the role of sentinel sites, representative of different epidemiological conditions. In these HFs, blood, stool, urine or nasopharyngeal samples will be regularly collected from targeted sub-groups of children at risk of diseases of specific interest for surveillance (e.g. watery diarrhea to detect cholera or unspecific fever to detect dengue). In these sentinel sites, when attending a child presenting with one of the syndromes of interest (based on the data entered in the ePOCT+ tablet), the HCW will automatically see an additional screen requesting to collect the necessary additional sample (e.g. stool in case of diarrhea or blood in case of unspecific fever), if the patient has provided informed consent for that.

If a child presents a syndrome of specific interest for surveillance (e.g. fever and rash) due to pathogens that cannot be detected based on a day 0 sample only (e.g. *Rickettsiosia* or *Leptospira*), he/she would be asked to return to the facility at day 7 or later and provide an additional sample. In such rare cases, the child would most likely not be cured at day 7 and would need to return to the facility for further care anyway. If the result turns out to be positive for one of these pathogens, the child will be proposed specific treatment if necessary (see chapter 8.1.3).

In addition to sentinel sites, machine learning anomaly detection will be developed to visualize spatiotemporal clusters of events, which aims to help clinicians detect disease outbreaks (e.g. fever combined with abdominal tenderness in a cluster of households may be indicative of an outbreak of typhoid or another intestinal pathogen). The use of additional available spatiotemporal data sources will be explored in further developing the surveillance platform based on well-known risk factors of

diseases in the area that are prone to outbreaks. For example, remotely sensed data from satellite imagery may be incorporated to monitor environmental conditions (e.g. land surface temperature, rainfall, vegetation cover) known to influence seasonal patterns of some diseases. Likewise, information about the locations of water and sanitation features can be incorporated to help identify areas that are at increased risk of waterborne or water-related diseases. In response to the spatiotemporal analyses, at a later stage of the surveillance study, we may expand surveillance sample collection beyond the sentinel sites using a mobile surveillance team.

3.4 Mixed-methods operational research

Throughout Phases 1 and 2, as well as during the last year of the project, mixed-methods studies will be conducted in HFs and communities, as well as at district and national levels, to understand the contextual factors that facilitate or impede the implementation, sustainability and scale-up of digital health interventions such as ePOCT+ in PHC settings. Examples of such factors may include socioeconomic and ecological profiles of communities, working conditions of the clinicians in the HFs, among others.

3.4.1 Cross-sectional quality of care surveys

Before the start of phase 1, during the second half of phase 1, and around 6 months after the beginning of phase 2, three cross-sectional surveys in a subset of randomly selected HFs will be performed to assess the quality of the consultation done by HCWs and their level of compliance to the algorithm (and thus to guidelines). Among the 40 HFs included in Phase 1, 18 HFs (10 in Morogoro and 8 in Mbeya, half of them being intervention HFs and the other half control HFs) will be randomly selected. Observers will be trained to attend and observe (without interfering) 25 consecutive consultations with children aged 1 day to 14 years per HF, thus a total 450 HCW/child pairs. Changes over time will be assessed during the three cross-sectional surveys. Before-and-after intervention analysis will be performed between the baseline analysis and the phase 1 intervention arm HFs, as well as between the control arm HFs in phase 1 and those same HFs that will then use the ePOCT+ tool in Phase 2. For the9 intervention HFs, this will provide another way (besides data recorded through the ePOCT+ tool) to measure potential improvement in the quality of the consultation over time. The tools for the quality of care surveys can be found in **Appendix 1**.

3.4.2 In-depth interviews, focus group discussions and surveys with health care workers

Interviewees (health providers) will be selected through a mix of purposive and random sampling. Based on the data collected through the medAL-monitor tool, we will select health facilities based on relevant criteria such as levels of uptake of and compliance with the algorithm. Within the health facilities, providers will be randomly selected among those available on that day.

Interviews will be conducted using a semi-structured interview guide developed for the study. The questionnaire will cover several themes, such as 1) experience using the ePOCT+ tool, 2) management of tablets in the facility, 3) training on the content and use of the algorithm, 4) experience with the e-learning modules and the supervision and mentorship tool (medAL-monitor).

For each question, probes will be used to generate further explanations from study participants. Each interview will be conducted as an informal discussion with open questions, leaving room for the HCW to express additional elements. In order to reduce study bias, participants will be strongly encouraged to be as open as possible in expressing their opinion and reassured on full confidentiality. The tools for the interviews and focus groups can be found in **Appendix 2**.

To complement the qualitative data, we will quantitatively evaluate the impact of using the ePOCT+ tool and the additional training and mentorship provided through the project on clinicians' competencies and confidence in their skills and decisions in Phase 1. The initial 3-day training will be implemented with a pre- and post- test administered to the participants. The e-learning platform will also come with short tests at the end of each module for the learners to self-assess their

understanding of the content. Lastly, baseline and end-line assessments and surveys will be used to compare intervention and control facilities. Similar assessment tools will be used in Phase 2, except a control group will not be available for comparing the difference in clinical competencies between baseline and end-line.

3.4.3 Caregiver and patient exit interviews

Structured exit interviews will be conducted with caregivers of sick children (as well as potentially older children aged 9-14 years) who attended HFs that are part of the study. A purposive sampling method will be used to select a wide range of children treated for different pathologies at participating HFs. Face-to-face interviews will be conducted using a semi-structure questionnaire. Themes that will be explored include: 1) quality of care received; 2) influence of the tablet on the interaction between clinician and patient/caregiver, 3) Confidence in treatment and management using ePOCT+ and 4) Impact of the use of ePOCT+ on future health seeking behavior. The questionnaires are adapted from the Service Provision Assessment (SPA) sick child client exit interviews (USAID et al. The Demographic and Health Surveys Program), a methodology recommended by the WHO. The tools for the exit interviews can be found in **Appendix 2**.

3.4.4 Focus group discussions with selected community groups

Focus group discussions will be organized with members of the community regardless of their experience with the use of ePOCT+ for clinical consultation. This is meant to gather unbiased opinions, perceptions, and attitudes of community members toward the use of ePOCT+ and digital health tools in general. The tools for the interviews and focus groups can be found in **Appendix 2**.

3.4.5 In-depth interviews with members of the CHMTs

Development and implementation of the medAL-monitor and the medAL-outbreak tools will be done in close collaboration with the district team and the HMIS department of the MoH from the very beginning. Once implemented, members of the CHMT will be interviewed about the impact of medAL-monitor on their ability to supervise and mentor HF staff. Similar interviews will be conducted with members of the HMIS department responsible for surveillance regarding the impact of the medAL-outbreak tool on their ability to detect and manage outbreaks. For example, they may be asked if the respective tool is accessible with internet connectivity available to them, if the indicators displayed in the dashboards are relevant and appropriate, and if the information displayed is clear, easily interpretable, and actionable. The interviews may be conducted several times for the purpose of continuous improvement of the tools and for final evaluation of their effectiveness. The tools for the interviews and focus groups can be found in **Appendix 2**.

3.4.6 Economic and ecological evaluations

Ecological and human impact

An ecological and human impact evaluation of the project will be assessed in collaboration with the “Sustainability Center” and the “Institute of Earth Surface Dynamics” of the University of Lausanne, to ensure that the intervention has limited impact on (or is even beneficial to) the environment and global warming. Indeed, the CO_2 emissions and pollution due to the manufacturing and use of tablets might be lower than that due to the manufacturing and international and national transport of (unnecessary) antibiotics and HF re-attendance visits or hospitalizations. Life Cycle Analysis (LCA) is a systematic study of the environmental impacts of product systems, which analyzes energy and material flows during the whole life cycle: extraction and acquisition of raw material, use, end-of-life treatment and final waste disposal (Frischknecht et al. 2007). Through such a systematic perspective, the displacement of potential environmental loads between the different stages of the life cycle or between

particular processes can be identified and avoided. The International Organization for Standardization published international standards on LCA, which gives a framework for LCA and guarantees the transparency of the analysis (see ISO 14040; 2006A and 2006B). This methodology is fully applicable to a project development.

Cost analysis

Cost analysis will compare the cost of care provision under routine conditions and with the use of ePOCT+. Costs will include costs to patients, providers, health system, and project implementation (Yukich et al. 2010). Costs of resource inputs for providers will be determined on the basis of the Tanzania current pharmaceutical and supply price list and interviews with the appropriate financial managers of the Dar es Salaam City Medical Office of Health. Patient costs will be valued according to self-reported expenditures and lost income due to the travel to and time spent in the HF. Costs of project implementation will include tablets and the associated IT infrastructure, initial and refresher trainings and supervisory visits, additional laboratory tests, but will exclude specific research costs.

4 STUDY AREA

4.1 Study sites

This study will be conducted in the Morogoro region (Mlimba, Ifakara and Ulanga districts) and in the Mbeya Region (Mbeya rural district and Mbeya city council) of Tanzania (**Figure 6**). Key health indicators of the selected regions compared to national average are outlined in **Table 1**. Since the previous randomized efficacy study was performed in urban primary health care centers in Dar es Salaam, we chose rural and semi-urban areas to extend the generalizability of the evaluation of the intervention. To evaluate whether the effect of the intervention varies depending on the level of malaria transmission, we chose areas with low (Mbeya rural district, Mbeya city council) and moderate (Mlimba, Ifakara and Ulanga districts) malaria endemicity. A direct train connection between Ifakara and Mbeya (TAZARA) will facilitate travel of investigators between the two sites.

Both, Ulanga and Mlimba Districts are found in the South-eastern region of Tanzania. Ulanga DC has a population of 265’000 inhabitants while Mlimba DC, which was previously called Kilombero DC has a population of 410,783 with the headquarters in Mngeta. Ifaraka Town Council is part of Mlimba DC with its own administration. Main economic activities in the two districts is subsistence farming mainly rice and smaller proportion of pastoralists who migrated from the north and center of the country. Most economic activities in Ifakara Town Council include agricultural trades, farming and provision of higher education in the biomedical field.

Mbeya DC is among seven districts of Mbeya region in the Southern highlands of Tanzania with a population of 366,986, with the headquarters in Inyala and a total of 74 total health facilities. The main economic activities are subsistent farming (commercial and food crops) and livestock keeping. Mbeya city is estimated to have a population of 471,971 by 2018, and it is the capital, commercial city of Mbeya.

Figure 6: Map of Africa and Tanzania (A) with study regions (B) and districts (C and D) highlighted.

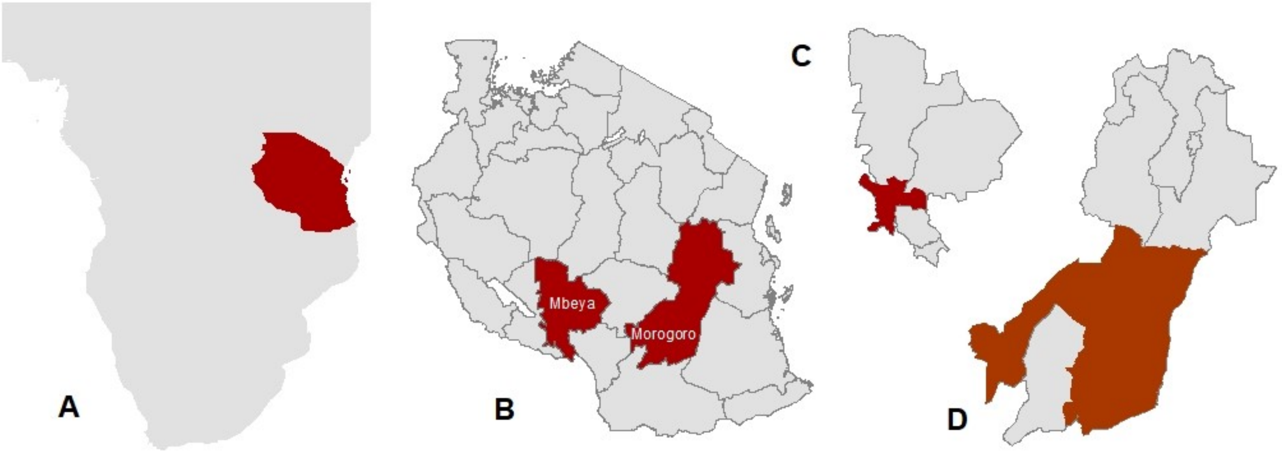

Table 1: Key indicators of child health in Morogoro and Mbeya regions

Indicator	Morogoro region	Mbeya region	National average
Under five mortality rate (per 1000 live births)[1]	163 (2002)	165 (2002)	153 (2002), 81 (2010)
Malaria prevalence in children aged 6-59 months (2017)[2]	9.5%	4.0%	7.3%
HIV prevalence (Age 15+; 2017)[3]	4.2%	9.3%	7.0%
Pneumococcal vaccine coverage (2017)[4]	81%	67%	99%
DTP - Hib - HepB[3] (Diphtheria, tetanus, pertussis, Hemophilus influenzae type B, and hepatitis B) vaccine coverage (2017)[4]	110.4% (2018)	96.3% (2018)	99%
Prevalence of low hemoglobin in children (<8g/dl)[2]	4.7%	1.4%	3.6%
Percentage of children with fever within the past two weeks[2]	18.9%	16.2%	20.4%

[1]National Bureau of Statistics, Tanzania; available at: http://tanzania.opendataforafrica.org/TZSOCECD2016/social-economics-of-tanzania-2016?region=1000130-mbeya&indicator=1007140-under-5-mortality-rate-deaths-per-1-000-live-births

[2]Ministry of Health, Community Development, Gender, Elderly and Children (MoHCDGEC) [Tanzania Mainland], Ministry of Health (MoH) [Zanzibar], National Bureau of Statistics (NBS), Office of the Chief Government Statistician (OCGS), and ICF. 2017. Tanzania Malaria Indicator Survey 2017. Dar es Salaam, Tanzania, and Rockville, Maryland, USA: MoHCDGEC, MoH, NBS, OCGS, and ICF. Available at: https://dhsprogram.com/pubs/pdf/MIS31/MIS31.pdf

[3]Tanzania Commission for AIDS (TACAIDS), Zanzibar AIDS Commission (ZAC). Tanzania HIV Impact Survey (THIS) 2016-2017: Final Report. Dar es Salaam, Tanzania. December 2018. Available at: THIS 2007: https://phia.icap.columbia.edu/wp-content/uploads/2017/11/Tanzania_SummarySheet_A4.English.v19.pdf

[4]USAID. Tanzania Immunization Fact Sheet. July 2018. Accessed from: https://www.usaid.gov/sites/default/files/documents/1860/Immunization_Fact_Sheet_July_2018.pdf

4.2 Selection of health facilities

PHC facilities are defined as the first point of contact to seek curative services. Facilities eligible for randomization in phase 1 will be determined in collaboration with the Regional Health Authorities prior to the start of the study. Permission for facility participation in the study and agreement to implement care practices as per randomization assignment at the level of the facility will be ascertained from HF administrative leadership prior to randomization. In phase 1, we anticipate enrolling 40 HFs (20 per arm), 24 in the Morogoro Region and 16 in the Mbeya Region. In phase 2, we plan to enroll 60 additional HFs, to reach a total of around 100 HFs.

<u>Inclusion Criteria:</u>

- Government and private government-designated Primary care HFs (health posts, dispensaries and health centers).
- Located in the Mlimba, Ifakara and Ulanga districts (Morogoro Region) or in Mbeya Rural district and Mbeya city council (Mbeya Region).
- Seeing at least 20 children aged 2 months to 5 years per week on average over the past 6 months (to facilitate participant recruitment).

<u>Exclusion Criteria:</u>

- Secondary and Tertiary HFs (district, regional, zonal and specialized hospitals).

5 STUDY POPULATION

5.1 Sick children attending PHC facilities

All patients aged 1 day to 14 years that present to the participating PHC facilities for an acute medical or surgical problem will be included into the study.

The ePOCT+ medical algorithm comprises evaluation and management content for acute medical and surgical conditions for children aged 1 day to 14 years. The lower limit of 1 day has been chosen to exclude peripartum conditions and immediate postnatal care; neonates who present to care immediately after birth (within the first 24 hours of life) will hence not be managed using ePOCT+ and will not be included into the study. The upper limit of 14 years has been chosen to be in line with the Tanzanian and WHO upper age range for adolescents.

The algorithm does not include chronic disease management or content to inform the delivery of routine preventive health services independent of evaluation and management of an acute problem. The algorithm also does not cover routine neonatal visits.

No specification of the primary complaint or symptoms at presentation will be required for eligibility and inclusion criteria were kept as broad as possible so as to maximize the generalizability of our findings.

<u>Inclusion criteria for Phases 1 and 2:</u>

- Aged 1 day (24 hours) to 14 years
- Presenting for an acute medical or surgical condition

<u>Exclusion Criteria for Phases 1 and 2:</u>

- Presenting for scheduled consultation for a chronic disease (e.g. HIV, TB, NCD, malnutrition)
- Presenting for routine preventive care (e.g. growth monitoring, vitamin supplementation, deworming, vaccination)
- Caregiver unavailable, unable or unwilling to provide informed consent (except for older children who can provide assent with an adult witness during the consenting process).

* This does not impede the child to be managed using ePOCT+ but will lead to erasing of the electronic data at the end of the consultation and no follow-up via phone call or SMS / home visit will be made at day 7.

5.2 Other stakeholders and/or beneficiaries

Other stakeholders and/or beneficiaries will be included in observational mixed-methods studies. HCWs attending to sick children and caregivers of these children will be involved in observational studies evaluating quality of care and interviews evaluating their experience with the ePOCT+ application. Communities where these children live will be involved in surveys and interviews to evaluate their perception of the intervention and any potential changes of the health system overall as a result of the intervention. Key actors of the health system (CHMTs of Morogoro and Mbeya regions, HMIS department and IDSR staff responsible for surveillance and data reporting, clinical directorate staff responsible for clinical services, computer scientists at the University of Dar es Salaam, research institutions in Tanzania involved in the ML training program, medical experts working at the national level) will also be interviewed about their perception of and experience with any components of the project that are relevant to their functions. Only those who have not had any interaction with the project (i.e. any of the electronic tools being implemented) or are unwilling to participate will be excluded from these additional studies.

6 DESCRIPTION OF THE INTERVENTION

The intervention will include five activities, each supported by a specific electronic tool (**Figure 7**).

Figure 7: Electronic tools on tablets or PCs used in the DYNAMIC project to improve the clinical management of sick children and enhance M&E, disease surveillance and outbreak detection.

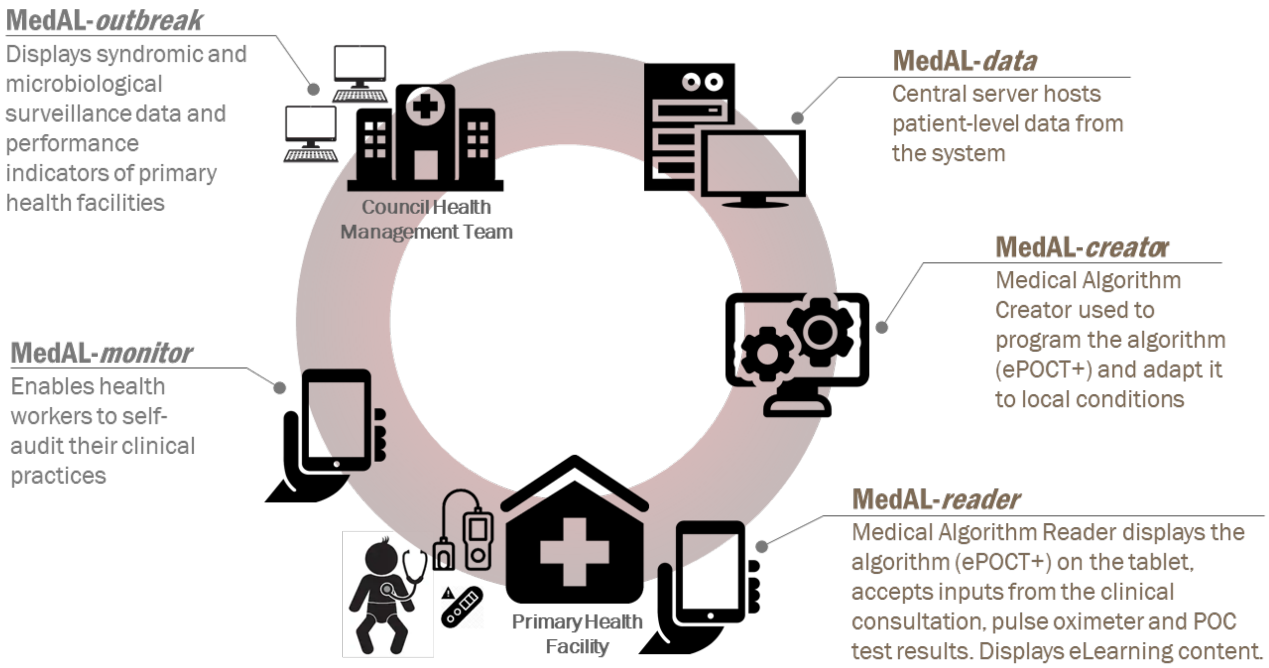

6.1 Management of sick children by health care workers using ePOCT+

Acutely ill children will be assessed, diagnosed and treated by routine clinicians guided by the ePOCT+ application, which will be loaded on a tablet or PC.

The intervention will be undertaken in routine PHC facilities in Tanzania and entails the implementation of the ePOCT+ tool for patient assessment and management, with provision of associated POC tests. Healthcare providers will use ePOCT+ throughout their medical consultations for children aged 0-14 years presenting with an acute medical or surgical problem. In case of a re-attendance visit between day 0 and day 7, ePOCT+ will be used for managing the child in the same way as during the initial visit, as the algorithm takes into account duration of symptoms and previous treatments provided. Equipment, reagents, supplies/consumables for all laboratory and diagnostic tests included in the algorithm that are not currently part of the standard of care in this setting, will be made available to providers throughout the duration of the study in the intervention arm.

The CDSA of ePOCT+ is already based on applicable Tanzanian national and international guidelines for pediatric clinical management (TZ version of IMNCI 2014; WHO Pocket Book of Hospital Care for Children 2013; TZ National Guidelines for the Diagnosis and Treatment of Malaria 2014; TZ Standard Treatment Guidelines and Essential Medicines list for Children and Adolescents 2018; Standard Treatment Guidelines & National Essential Medicines List Tanzania Mainland 2017 (Adults);TZ National Guideline for Neonatal care and Establishment of Neonatal Care Unit 2019; TZ National Guidelines for the Management of Tuberculosis in Children 2016; WHO IMAI guidelines 2009). Before implementation, the medical algorithm will be reviewed by Tanzanian key experts in child health (Pediatric Association of Tanzania; Ministry of Health; Muhimbili National Hospital).

The majority of the algorithm content was previously validated in Tanzania (see background section). The exact medical content of the ePOCT+ algorithm will be published online, on the website that will be dedicated to the DYNAMIC project. Any subsequent modifications made to the original algorithm will be published as soon as they are implemented in HFs.

Specifically, the tool will guide clinicians on:

i) Decision on immediate referral to hospital versus home-based treatment
ii) Medical history (questions to ask caregivers and/or children)
iii) Physical exam (signs to look for)
iv) POC tests (which should be performed in a given clinical context (see **Table 2**)
v) Diagnoses
vi) Treatments (including medicines and supportive treatments)
vii) Counseling (explanation on final diagnoses, treatments, and follow-up)

Biosensors (presently pulse oximeter and hemoglobinometer) and rapid tests (presently malaria, HIV, semi-quantitative CRP, urine dipstick) will have to meet requirements for implementation at PHC level, including adequate measurement accuracy, stability in high-temperature and dusty settings, as well as affordability. Once adequate biosensors and POCTs are identified, manufacturer negotiations are complete, and the regulatory framework is in place, they will be used by HCWs upon recommendation provided by ePOCT+ (**Table 2**).

Table 2: Point-of-care tests included in the ePOCT+ CDSA

POCT	Targeted patient group	Rationale
Pulse oximetry (POX)*	Patients with respiratory complaints and severe disease<br>Young infants with suspected severe disease	POX may improve detection of children with hypoxemia<br>In neonates and young infants, among whom accurate respiratory rate can be difficult to ascertain and short instances of rapid breathing or apnea can be physiologic, POX may improve diagnosis and classification of severe illness
Hemoglobin (Hb)*	Patients ≤ 5 years with fever<br>Patients 6-14 years with malnutrition, palmar pallor or conjunctival pallor	Regular Hb measurements in febrile children will improve detection of severe anemia, which is a marker of severe disease for a variety of disease pathways, including severe malaria, sickle cell disease, and severe bacterial infections
Malaria rapid diagnostic test (mRDT)	Patients with fever	Malaria is a potentially fatal disease that must be excluded in patients presenting with fever, or in a sub-group of febrile patients at high risk in very low endemic areas, as per current WHO guidelines
C-reactive protein (CRP)*	Febrile patients at risk for bacterial pneumonia (based on clinical and demographic features)<br>Febrile patients without focal clinical signs<br>Febrile patients with joint or extremity pain<br>Infants aged 1-2 months with suspected pneumonia or bacterial infections	CRP improves targeting of antibiotic prescription in children with respiratory symptoms, with fever without focal signs, and with joint/extremity pain at risk of septic arthritis or osteomyelitis<br>CRP may improve specificity of diagnoses among young infants with non-specific signs of possible severe illness and inform targeted antibiotic treatment and appropriate supportive care
HIV rapid test	Patients with possible exposure to HIV (mother with HIV or mother with unknown HIV status and unavailable for testing, or the patient is sexually active).<br>Informed consent from the caregiver must be acquired.	Tanzania national AIDS control program promotes provider-initiated HIV testing to all children presenting at HFs. Identifying HIV early to start antiretrovirals in a patient is essential to improve morbidity/mortality and prevent further transmission of HIV.
Urine dipstick / Urinalysis	Febrile patients <3 years without focal clinical signs<br>Febrile patients > 3 years with complaints suggestive of urinary tract infection.	CRP does not reliably rise in children with febrile urinary tract infections; febrile children <2 years should be assessed for febrile urinary tract infections per national guidelines.<br>In older patients, for which urine collection is easier, a urinary tract infection can be excluded using a urine dipstick.

*POX, CRP and Hb cuvettes will be provided by the DYNAMIC project, all other tests are routinely available at PHC

The ePOCT+ CDSA guides the clinician step-by-step through the consultation process. The algorithm is able to integrate all the information from the consultation and to provide diagnostic classifications and related treatment recommendations. The application will be radically different from the existing medical software as it will allow clinicians to freely navigate up and down the algorithm, adding or modifying clinical information of any type (exposures, symptoms, signs, lab test results) at any time, in order to follow the natural consultation process (rather than forcing a clinician to assess the patient condition in a fixed order).The interface will also be much more user friendly, available in all necessary Tanzanian national languages, and allow integration of alerts of ongoing epidemics, definitions or translation of medical terms in local languages, explanations and illustrations (pictures or videos) of symptoms or signs that are difficult to assess and those that are specific to rare diseases, and automated teaching information on why a certain disease is being considered or not considered in a particular patient.

In addition to the patient management algorithm, the overall ePOCT+ ecosystem contains a patient registration module and a triage module that can be used by different providers within the same HF, using tablets that are connected via WIFI. The purpose of the triage module is to prioritize patients that most urgently need medical attention and to allow task-shifting and optimization of care processes. The ePOCT+ triage module is based on the WHO ETAT guidelines and the IMCI-based SCREEN tool and was vetted in terms of reliability and feasibility through a Delphi process involving 30 providers involved in primary care in Tanzania. The triage module can be switched on and off within the application to allow flexibility to the facility staff to decide on how best to organize their workflows and task-shift (e.g. by having a lower level staff member recording all vital signs or performing the necessary rapid tests) so that the clinician can gain time during the consultation for tasks that cannot be delegated. Based on previous experience, the average consultation time while using ePOCT+ is expected to be 15 minutes.

ePOCT+ can be used on a tablet or computer, depending on the information technology platform already in use in the HF and level of comfort of providers with various electronic devices. We will perform pilot testing of the ePOCT+ tool in select HFs with direct supervision from qualified medical personnel before the start of study recruitment. The aim of this pilot phase is to ensure that ePOCT+ is understandable and acceptable to the users and that its flow conforms with the consultation process. The aim is also to optimize the interface and user experience of the application.

No major modifications in the algorithm clinical content are foreseen for the duration of phase 1 but minor changes may be made in case of malfunctioning of the application.

6.2 Regular update of ePOCT+ to improve accuracy and adapt to epidemiological changes

The ePOCT+ algorithm will be programmed into an electronic software support system. An in-depth internal IT validation will be performed through the interface of MedAL-Creator that allows direct visualization of the programmed algorithm.

For this, a specific software solution is currently being developed for the Tanzanian setting which has three main components (see **Figure 8**):

- The clinical algorithm management system that will allow to manage, reference, modify and deploy the different versions of the algorithm into a mobile device (called MedAL-Creator)
- The application (tablet or PC) that will contain the algorithm and be used for data acquisition by the HCW (called ePOCT+), as well as automated capture of results from biosensors and rapid tests (called MedAL-Reader).
- The data management system that will aggregate and manage the data into a central server with a database (called MedAL-Data).

Figure 8: General architecture of the MedAL-Creator software solution

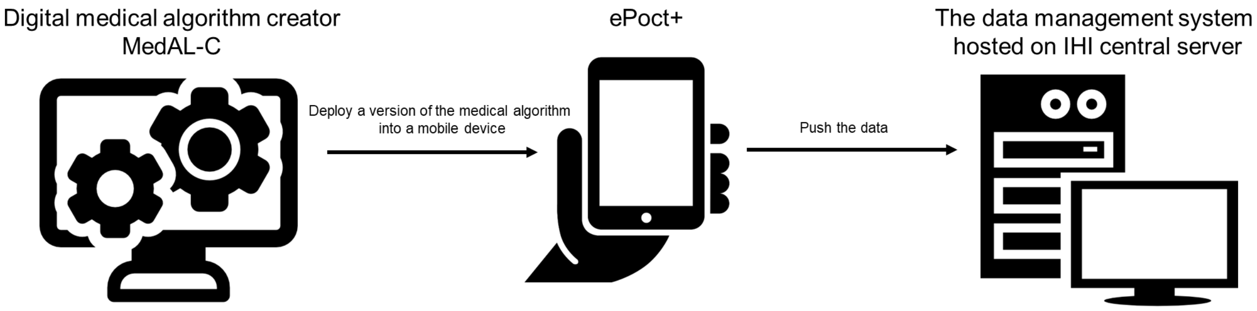

During phase 2, the algorithm will be modified according to trends in the collected data detected using ML or conventional statistical methods. The modifications will be designed by clinical experts based on the clinical and microbiological surveillance data collected during the study. Approved changes will then be integrated into the algorithm using MedAL-Creator that allows physicians without any IT programming skills to modify the decision tree and a new version of the algorithm released. Each version will then be loaded on the tablets or PCs of the relevant HCWs (for example of a certain region where an epidemic is occurring) with a message explaining the rationale and evidence behind the change in the algorithm.

6.3 Initial in-person training and e-leaning modules available on tablets

Initial centralized training on essential clinical skills, the evidence and guidelines behind the content of the algorithm, and the practicalities around the use of the electronic tool and POCTs will be provided to clinicians working in intervention facilities. Re-training of incoming personnel will also be implemented.

To continue improving providers' clinical skills throughout the project, e-learning modules will be developed. Such modules will help clinicians assess important clinical signs, especially pediatric danger signs (e.g. chest indrawing). First, we will perform a needs assessment for additional training among HCWs. Next, we will screen available video tutorials for suitability and adapt and translate them into the Tanzanian national languages. We will also develop additional training modules on clinical topics for which no training materials are available. The e-learning modules will then be pilot-tested among HCWs before routine deployment, and then deployed onto the clinicians' tablets and integrated with the ePOCT+ workflows.

6.4 Supervision of health facilities by the CHMT using medAL-monitor

To enable better supervision of HCWs by the district medical team, we will also develop and deploy an electronic supervision tool called medAL-monitor. We will define clinical performance indicators, based on the data collected through ePOCT+, and integrate them into the e-supervision tool. The latter will contain a dashboard to visualize these indicators per HF and per HCW over time, as well as a system to generate forms for targeted feedback to HCWs that will allow them to perform self-audits. The tool will also enable and encourage more frequent two-way communication between remote HCWs and the district team. The supervision tool will be pilot-tested with the district team and HCWs before routine implementation.

6.5 Disease surveillance and outbreak detection by district health authorities using medAL-outbreak

The medAL-outbreak disease surveillance and outbreak detection system will be made available to the district (and national) health authorities (Phases 1 and 2) for them to monitor certain health

indicators over time, be alerted if indicator values are higher than expected for a given month in a given location, suggesting a potential outbreak, and be able to decide if disease control measures are needed.

Several versions of the dashboard will be developed: for the HFs, the district level and the national level. The dashboards will have several tabs for different types of health statistics: general information, main symptoms or syndromes, acute diseases, chronic conditions, and clinical management including treatment and referral.

Specific indicators for self-audits (HF level), supervision (district level), monitoring and evaluation (district and national level) and disease surveillance (district and national level), will also be available. Important M&E indicators related to the impact of the present intervention that can all be stratified by geographical area, time period and age group and are similar to the Tanzanian HMIS indicators for child health, are given as an example in **Table 3**.

In addition to the M&E indications, indicators for early epidemic warning and response will be selected with and provided to CHMTs through dedicated web-based dashboards (medAL-monitor/medAL-outbreak). These indicators can then be transferred to the DHIS2 platform and to national health authorities. MoH CDEC will thus be able to compare the data provided by their DHIS2 dashboard with that provided by the medAL-outbreak dashboard, for the 3 districts where ePOCT+ will have been implemented. Once the MoH will consider that they are ready to do so, interoperability between the two systems will be enabled.

Table 3: Examples of M&E indicators that will be possible to display in the medAL-monitor/medAL-outbreak dashboards based on ePOCT+ data

**Consultations**	
	• Number of outpatient consultations
**Main indicators**	
	• Number and % of deaths<br>• Number and % of children referred to hospital<br>• Number and % of children with malaria (severe and non-severe)<br>• Number and % of children with bacterial pneumonia (severe and non-severe)<br>• Number and % of children with diarrhea (severe and non-severe)<br>• Number of measles cases<br>• Number and % of children prescribed an antibiotic (by level of disease severity)<br>• Number and % of children prescribed an antimalarial (by mRDT status)<br>• % of final diagnoses made by HCWs for which the appropriate treatment is prescribed
**Danger signs**	
	• % of children with at least one danger sign<br>• % of children with each type of danger sign<br>• % of children with at least one danger sign prescribed an antibiotic<br>• % of children with at least one danger sign prescribed an antimalarial
**Among children with cough/difficulty breathing**	
	• Number and % of children with cough/common cold, viral pneumonia and bacterial pneumonia<br>• Median and distribution of respiratory rate measurements<br>• % of fast breathing among children with cough/difficult breathing<br>• % of chest indrawing among children with cough/difficult breathing<br>• Median and distribution of oxygen saturation measurements<br>• % of hypoxemia among children with fast breathing or chest indrawing<br>• % of children with bacterial pneumonia prescribed an antibiotic<br>• % of children with viral pneumonia prescribed an antibiotic<br>• % of children with cough/common cold prescribed an antibiotic<br>• % of children with bacterial pneumonia prescribed the first line antibiotic
**Among children with diarrhea**	
	• % of children with diarrhea<br>• % of dehydration among children with diarrhea<br>• % of dysentery/persistent diarrhea among children with diarrhea<br>• % of children with diarrhea prescribed ORS/zinc<br>• % of children with diarrhea prescribed an antibiotic
**Among children with fever/elevated temperature**	
	• % of children with confirmed versus clinical malaria<br>• % of children with positive mRDT result<br>• % of mRDT-positive children prescribed an antimalarial<br>• % of mRDT-negative malaria children prescribed an antimalarial<br>• % of children with malaria prescribed the first line antimalarial
**Screening for chronic conditions**	
	• % of sick children tested for anemia<br>• % of sick children assessed for malnutrition<br>• % of sick children tested for HIV<br>• % of children with anemia (severe and non-severe)<br>• % of children with malnutrition (severe and moderate)<br>• % of HIV-positive children

7 OUTCOME MEASURES AND INDICATORS

7.1 Outcome measures and indicators for Objective 1 (Improve the integrated management of sick children)

The primary aim of the cluster-randomized study is to concurrently evaluate whether the use of ePOCT+ for case management of sick children in primary care, compared to routine care:

(i) results in less antibiotic prescriptions at initial consultation;
(ii) is non-inferior in clinical outcomes at day 7.

Rationale for choosing the primary aim and related primary analysis:
The second-generation ePOCT CDSA showed potential as an antimicrobial stewardship intervention for managing febrile children aged 2 months to 5 years, in a controlled trial setting in health facilities conducted in Dar es Salaam in 2015-16. Management with ePOCT resulted in a drastic reduction in (unnecessary) antibiotic prescriptions while maintaining similar, or possibly achieving better clinical outcomes by day 7. The current study seeks to generate evidence that would be more generalizable to a wider Tanzanian population. It aims at assessing the effect of an intervention package including the i) extended CDSA, called ePOCT+, ii) training and iii) supervision, when implemented under conditions closer to those that would be encountered in routine practice. ePOCT+ is an expansion of ePOCT that includes content for managing a wider age group (1 day to 14 years) with more comprehensive medical concerns (any acute medical or surgical problem). To determine whether ePOCT+ is a successful antimicrobial stewardship intervention, a significant reduction (by at least 25%) in antibiotic use without a negative effect on patient outcomes needs to be demonstrated (Gillespie et al., 2018).

Co-primary outcome measures for the cluster randomized controlled study in Phase 1:

(i) % of children cured at day 7 (according to caregivers contacted through a phone call or home visit) in the intervention group (ePOCT+) as compared to control group (routine care)

(ii) % of children prescribed an antibiotic at initial consultation, as reported by HCWs, in the intervention group (ePOCT+) as compared to control group (routine care)

Main outcome indicators (monitored over time and across geographical areas in all HFs in Phase 2)**:**

- % of children cured at day 7
- % of children prescribed an antibiotic at initial consultation

Secondary outcome measures (Phase 1) **or indicators** (Phase 2)**:**

These measures or indicators include the evaluation of the effects of the use of ePOCT+ (versus routine care in Phase 1; over time and across geographical areas in Phase 2) for managing sick children aged under 15 years presenting to PHC facilities on:

Unscheduled secondary consultations:

- % of children with one or more unscheduled re-attendance visits at any HF by day 7

Severe outcome by day 7:

- % of children with non-referred secondary hospitalization by day 7
- % of children who have died by day 7

Primary referrals:

- % of children referred to hospital or admitted to inpatient ward at a health centre at initial consultation
- % of children who were hospitalized among those referred (i.e. referral completed)

Appropriate case management for malaria at initial consultation

- % of febrile children tested for malaria
- % of malaria positive children prescribed an antimalarial
- % of malaria negative children prescribed an antimalarial
- % of untested children prescribed an antimalarial

Appropriate case management for other conditions at initial consultation

- % of key symptoms and signs checked and diagnostic tests performed by HCWs
- Distribution of final diagnoses (including their severity) made by HCWs
- Concordance between treatment prescribed and final diagnoses made by HCWs
- Concordance between final diagnoses as proposed by ePOCT+ and final diagnoses by HCW *(intervention HFs only)*

Definitions used for the outcome measures and indicators of objective 1

- *Clinical cure is a positive answer to the question "Is the child cured?" OR "Has the child improved?" (if not cured) asked to caregiver through a phone call (or home visit) at day 7. Non-referred secondary hospitalizations (see definition below) will however be considered as clinical failures even if the child is already cured at day 7.*
- *Initial consultation is the first visit of a sick child for an acute problem at a HF participating in the study (and thus registered electronically); timeframe from completion of the initial visit up to midnight of the same day.*
- *Re-attendance visit is a consultation (not necessarily at a HF participating in the study) taking place from the day after initial consultation up to day 7 included. A re-attendance visit can be scheduled (proposed by the HCW on a certain day) or unscheduled (upon decision by caregivers).*
- *Primary referral is a decision of referring the child to hospital taken by the HCW at the initial consultation*
- *Secondary referral is a decision of referring the child to hospital taken by the HCW during a re-attendance visit taking place from the day after initial consultation up to day 7 included.*
- *Primary hospitalization is an admission to a hospital ward taking place the same day as the initial consultation*
- *Secondary hospitalization is an admission to a hospital ward taking place from the day after initial consultation up to the phone call at day 7 included. A non-referred secondary hospitalization is a direct visit to hospital without a re-attendance visit the same day at a HF participating in the study.*
- *Antibiotic prescription is any oral, intramuscular or intravenous (but not topical) antibiotic prescribed by a HCW during the initial consultation or a re-attendance visit*
- *Antimalarial prescription is any oral, rectal, intramuscular or intravenous antimalarial prescribed by a HCW during the initial consultation or a re-attendance visit*
- *Febrile child is a child with a history of fever and/or a high temperature*
- *Malaria testing is a malaria RDT or microscopy ordered by a HCW during an outpatient visit*
- *Day 7 is a range of 6-14 days post initial consultation to allow for completion of home visit for children whose caregiver reports having no phone / no phone number.*

Additional longitudinal analyses:

- Change in % of children prescribed an antibiotic over time
- Change in % of children with basic anthropometrics and clinical signs measured over time (intervention arm only)
- Change in % of consultations where ePOCT+ was used over time (intervention arm only)

7.2 Indicators for Objective 2: Improve the algorithm and adapt it to spatiotemporal variations

- Number of positive impact modifications in the algorithm identified by ML-based analyses
- Number of positive impact modifications tested through randomized sub-studies
- % of these positive impact modifications confirmed to be safe and useful by randomized sub-studies
- Number of modifications implemented by type of impact (better clinical cure, less severe clinical outcomes, less medicines or diagnostic tests needed, shorter consultation time…)

Definitions used for the indicators of objective 2

Positive impact modification is defined as a change in the algorithm expected to increase the clinical cure rate and/or decrease resources needed and/or to have another positive clinical or health system related impact.*

** Resources needed include but are not limited to medicines prescribed, diagnostic tests ordered by HCWs, or time spent to perform a consultation.*

7.3 Indicators for Objective 3: Enhance disease surveillance and outbreak detection

- Number of M&E and HMIS indicators based on individual data that can be visualized through the medAL-outbreak dashboard
- Average delay between the date of recording of clinical data and the date they are available on medAL-outbreak
- Number and type of epidemic alerts raised by medAL-outbreak
- % of these epidemic alerts registered by the district authorities for epidemic response

7.4 Indicators for Objective 4: Enhance M&E and supportive supervision and mentorship

- Number of M&E indicators based on individual data that can be visualized through the medAL-monitor
- Number of supervision and mentorship visits to HFs facilitated by medAL-monitor
- Number of HCWs reporting having used medAL-monitor for self-auditing their clinical practices
- Number of HCWs and members of CHMT reporting a positive experience with medAL-monitor

7.5 Indicators for Objective 5: Create a supportive environment for the use of clinical algorithms and disease surveillance tools

- Number of sick children attending primary care facilities managed by HCWs using the electronic ePOCT+ tool
- % of HCWs able to assess key clinical signs
- Frequency of use of medAL-outbreak by district team members
- Frequency of use of the medAL-monitor tool by district team members
- Perception by HCWs of the ePOCT+ tool and intervention and the number of improvements they propose for the tool or intervention
- Perception by caregivers and community members of the intervention and the number of improvements they propose
- Perception by district team members of medAL-monitor/medAL-outbreak and the number of improvements they propose for the tool
- Number of medical experts able to modify a clinical algorithm using MedAL-Creator

- Number of computer scientists able to maintain the software and the IT infrastructure for clinical algorithms
- Number of scientists able to analyze clinical data using ML
- Number of local students trained in applied ML for health care in resource-limited settings
- Perception of the current and future utility of ML to improve health care amongst clinical experts (deciding on algorithm changes) and health authorities
- Patient and provider expenditures related to care of acutely ill children included in the study
- Carbon footprint of the intervention or carbon-saving due to the intervention

8 STUDY PROCEDURES

8.1 Objective 1: Improve the integrated management of sick children

8.1.1 Recruitment, screening and informed consent procedure

As the intervention is implemented at the facility level, approvals from relevant authorities will be sought before engaging with persons in charge of HFs and HCWs. Sensitization meeting will be organized in the villages or suburban wards within the catchment areas of enrolled HFs. District and HF consent will be obtained for the intervention. HFs will be informed if they have been allocated to the intervention or control group and that, if allocated to the control group, they will benefit from the intervention a maximum of one year after the start of implementation, until at least the end of year 2022. Individual consent from caregivers will be sought for electronic recording of data and follow-up by phone call or SMS. Upon the start of the study, information will be provided in the waiting areas of facilities.

All children aged 1 day to 14 years presenting for an acute problem during or after working hours to a HF that is part of the study will be asked if they wish to participate in the study by the person usually doing registration of patients (using the registration module in the tablet) or another appropriate staff member trained in ascertaining consent. The consent process will be integrated into the routine work flow at the health facility. Caregivers will be asked consent for electronic data recording and follow-up by phone or SMS for their child. They will be informed that not providing consent will not affect the medical management of their child and, in intervention facilities, the child can still benefit from being managed by the ePOCT+ tool and that their data will not be used for research purposes. All caregivers will be provided a paper-based participant information sheet and consent form providing sufficient information to make an informed decision about their participation in the study. If the clinical condition of the child requires immediate treatment, this will supersede the written consent process, which will be conducted once the child is stabilized. The consent form will be retained as part of the study records.

After the completion of the Phase 1 study, if the benefits of the intervention are confirmed, in the routine implementation of ePOCT+ the individual consent process will be simplified (consent at district and health facility level will be maintained). District and health facility level consent (obtained in sequence) will be documented on the same consent form and copies will be kept at district level, health facility level, and with the investigators. The registration form will be reduced and integrated into the routine registration process. Consent will be limited to a verbal agreement that data recorded in the tablet during the consultation can be used for research. Ticking “yes” in the application means that consultation data will be sent to the research database. Ticking “no” means that the data will remain on the tablet/local data server and will not be analysed. Otherwise, all children will be treated in the same way (using the tablet). In case of any significant modifications in the algorithm content that require validation using a sub-study, in health facilities participating to the sub-studies, written consent procedure as outlined in Phase 1 will be maintained. Patients participating in the surveillance

sub-study will also undergo the full written consent process.

8.1.2 Management of sick children during the initial consultation

In HFs with dedicated staff for triaging patients, sick children will first be assessed by this person using the triage module of ePOCT+ in the tablet. If the child requires urgent treatment, the triaging person will immediately inform the attending HCW(s), so that she or he can give priority to that child over others waiting to be seen. If not, the child will queue normally and be attended by the next available HCW. As part of triage, the vital signs will be measured (temperature, respiratory rate, weight and MUAC) as well as, if the child fulfils certain clinical criteria, oxygen saturation and cardiac rate using the pulse oximeter. If there are no dedicated triage staff, the triage module will be de-activated and vital signs will be measured during the consultation.

The attending HCW will either manage the child using the ePOCT+ algorithm (in intervention facilities) or as per routine (in control facilities). If the child needs to go to another room for laboratory investigations (rapid tests or other), the HCW will be able to suspend the ePOCT+ algorithm and data recording in the tablet and attend another child in the meantime, opening a parallel session in the tablet. When the child returns with the test results, the HCW will resume the ePOCT+ algorithm and data recording of that child and continue the consultation normally. At the end of the consultation, the HCW, in both intervention and control facilities, will be asked to enter the final decision on referral, tests performed, final diagnoses and treatments prescribed (see chapter 8.1.6 on outcomes measurements). After the consultation, this information will be synced to the server at IHI offices as soon as there is internet connectivity.

Figure 9: Flowchart of health facilities and patients included in Phases 1 and 2

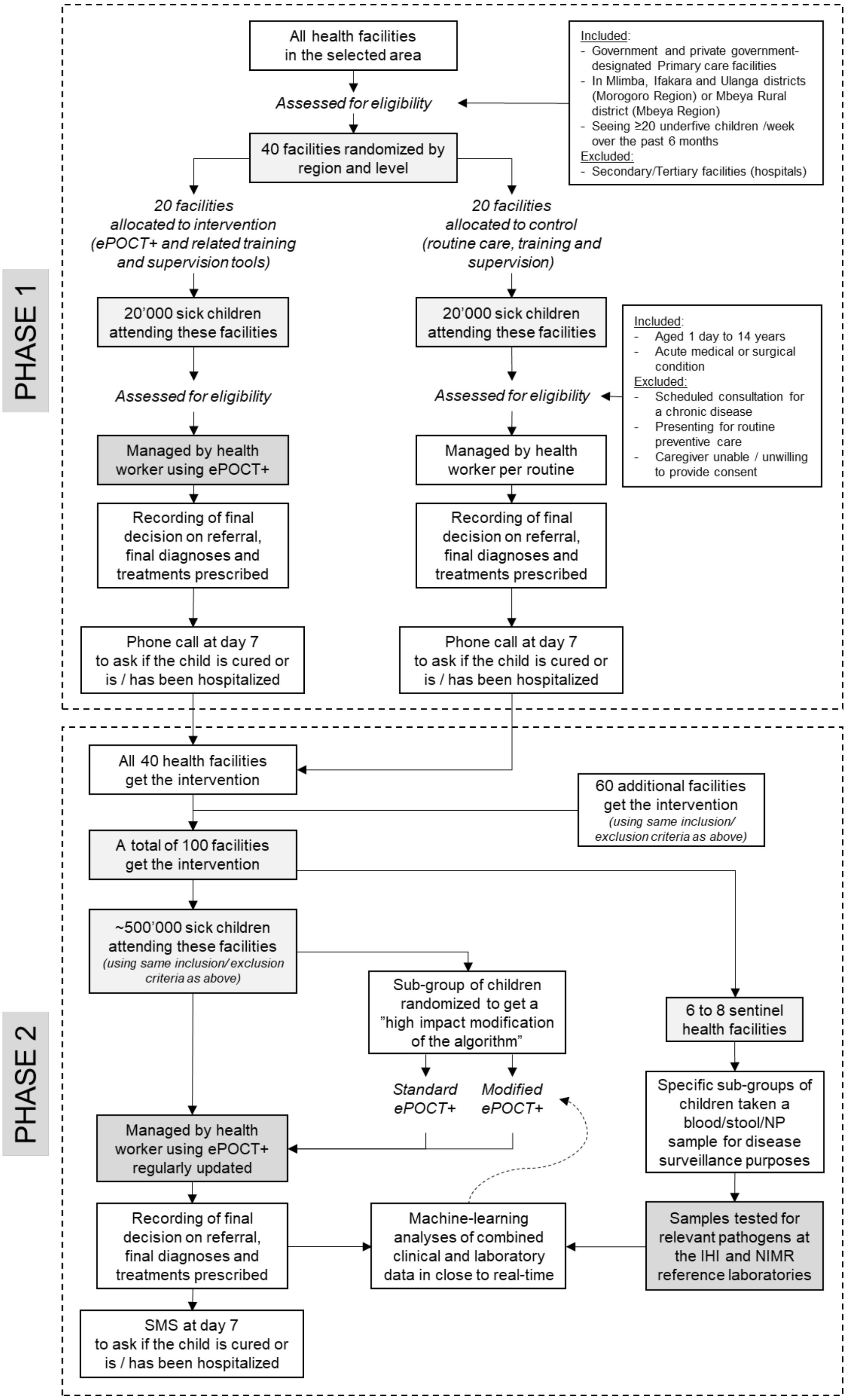

8.1.3 Re-attendance visits in case of persisting or worsening of symptoms

At initial consultation, the advice to caregivers on when to bring the child back to the HF is left at the discretion of the HCW. In intervention facilities, HCWs will be guided by the ePOCT+ tool, which recommends conditional follow-up based on WHO and national guidelines.
At re-attendance visits, whether scheduled or unscheduled, children will be managed according to the study arm to which they were initially assigned (ePOCT+ or routine care) in Phase 1, and with the support of ePOCT+ for all children in Phase 2. In case children still present fever at day 7, the algorithm will recommend in all cases to give an antibiotic to cover the possibility of an undetected bacterial infection. If surveillance laboratory results show that the child was suffering from a rare bacterial infection not covered by the antibiotic provided at day 7 (e.g. rickettsiosis or leptospirosis), the caretakers will be contacted to ensure that the child is indeed cured. If the child is still or again sick, caretakers will be asked to bring the child to the health facility to be assessed clinically and receive the necessary specific antibiotic.
If the child comes back to the same HF as for the initial consultation, the HCW will have access to the electronic data of that initial consultation that is saved in the HF tablets. If the child goes to a different HF that is participating to the study, the HCW will not have access to that data. Investigators will however be able to link data collected at different facilities (as long as they are part of the study) for a same child through the unique identification number provided to caregivers. If the child goes to a HF not participating in the study, no data will be available for that consultation. The ePOCT+ registration module, used at all participating facilities, will inquire about the type of visit (initial / scheduled re-attendance / unscheduled re-attendance) and the main reason for that visit.

8.1.4 Training of health providers on how to use ePOCT+

In intervention facilities, HCWs will participate in centralized in-person 3-day training course. They will receive general training on the epidemiology of diseases in Tanzania, the most important pediatric diseases and their clinical predictors, the content of the ePOCT+ algorithm and the importance of rational antibiotic and antimalarial use. General, key clinical skills will also be reviewed during this training and an introduction to the e-learning tool aimed at supporting these skills once they return to their facilities will be given. In addition, HCWs will receive training on how to use the ePOCT+ application on the tablet, using clinical case studies, as well as job aids for conducting and interpreting the results of POC tests which are not currently part of standard care at PHC level but are included in the algorithm (e.g. pulse oximetry and CRP rapid test).

During the month following this centralized training, HCWs will benefit from one visit for in-service face-to-face training during which trainers will accompany them in some of their consultations with real patients and help them optimize the way they use ePOCT+. Trainers will also support reorganization of workflows in the HF if necessary.

At the end of Phase 1, all HCWs from control HFs as well as those from the new participating facilities will receive the same training as described above. During both Phases 1 and 2, new personnel at study facilities will be trained through a dedicated module on the tablet and by their peers. This module will be available to all HCWs using ePOCT+ so that they can refresh their knowledge any time.

8.1.5 Supervision of health providers by the CHMT

The CHMT will be provided the medAL-monitor tool which is a dashboard available on PCs and tablets allowing them to monitor indicators (see **Table 3**) related to pediatric consultations taking place in the HFs enrolled in the study. Data on the child's or the HCW's identification will not be provided, only the name of the heath facility will be available. Based on these indicators, the supervision team will be able to better organize on-site visits to HFs, prioritizing the lowest performing ones, knowing in advance what areas of improvement to concentrate on. As the persons in charge of HFs, as well as

each HCW, will also have access to these indicators (fully anonymized except for their own data) to allow them to do self-auditing at HF level so that they are aware of their own performance in advance of receiving a CHMT supervision visit. The aim of the medAL-monitor tool is to stimulate HCWs' motivation and self-leadership to improve their skills and competencies, while enabling the supervision team to help them resolve remaining problems (Bessat et al. 2019).

8.1.6 Type of care, training and supervision provided at intervention and control facilities

In intervention HFs, HCWs will be guided by the ePOCT+ recommendations throughout the consultation. However, health care providers are ultimately responsible for the clinical management of all patients and may override recommendations of the algorithm and provide additional or withhold treatment according to their own clinical judgement. All concomitant diagnostic interventions and treatments will thus be left at the discretion of the provider but recorded in the ePOCT+ tool. In control HFs, routine care as currently implemented in Tanzanian primary care settings will be provided.
Before the start of Phase 1, all skilled HCWs who are engaged in triage, assessment, and medical management of sick children in both intervention and control facilities will receive training in study-related procedures, including consent for electronic recording of data, and documentation of the final decision on referral, tests performed, final diagnoses and treatments using the tablet. In intervention HFs, training on the ePOCT+ tool will be provided as described above while in control facilities, standard paper guidelines (IMCI and Tanzanian national treatment guidelines) will be provided and HCWs reminded that these guidelines should be followed during patient management. They will also be informed that, after the end of phase 1, they will receive the intervention exactly as HCWs of initial intervention HFs did.

In intervention HFs, HCWs will receive feedback on the data registered through the ePOCT+ tool, and the CHMT will be using medAL-monitor to better target supervision and support to facilities. In control facilities, supervision by the district team will be done using the available DHIS2 data as usual.

In both intervention and control facilities, it is assumed that usual diagnostic tests and treatments will be provided through the routine health care system. Control HFs will not receive additional POC tests that HFs in Tanzania would not normally have available in similar settings (i.e. CRP test). Care for children under the age of five years is free of charge while caregivers are expected to pay out of pocket for health care expenses of older children (unless expenses are covered under an insurance policy). However, when not available at the HF, patients must procure their medications from nearby pharmacies or other HFs. To allow testing of the intervention under conditions as close as possible to those expected for future implementation, we will not provide routine diagnostics or medications in either intervention or control facilities. However, we will ensure that immediate life-saving treatments are available and will support the facility staff in managing stocks through routine mechanisms.

8.1.7 Measurement of primary and secondary outcomes

Data captured on the tablets used by health care workers during the consultation

During the pilot phase before Phase 1 begins, tablets will be provided to HCWs in both intervention and control HFs. In intervention HFs the tablets will contain the patient registration module followed by the ePOCT+ algorithm that will guide them throughout the consultation process. In control HFs, tablets will only collect the patient demographics (see **Appendix 1**).

To determine the percentage of children prescribed an antibiotic or an antimalarial, as well as to know the final decision on referral and final diagnoses retained by HCWs, four questions will appear on the screen at the end of the consultation process, i.e. once the clinician has gone through the entire algorithm in intervention HFs or just after the patient registration module in control HFs. HCWs will be asked to document the final decision regarding the need for referral, tests performed and the results, decision on the date of follow-up (if needed), final diagnoses and final treatments prescribed. For each

medicine prescribed, the dosage and duration of the treatment will also be recorded (see **Appendix 1**).

Data captured through phone calls or home visits (Phase 1) or automated phone calls or SMS (Phase 2) to caregivers

For Phase 1, research assistants will be trained to administer a simple questionnaire by phone to caregivers of all children at day 7 (range 6-14) after their initial consultation with a HCW of participating facilities, regardless of re-attendance visits. If the phone follow-up fails, up to four additional attempts will be made on the subsequent days, after which they will be deemed lost to follow-up. These research assistants will be placed outside HFs and blinded to which facility the child has attended as much as possible. Data recorded during these phone interviews will be collected electronically and sent at least once a day to the central server of the study at IHI. For the question related to the clinical primary outcome measure (percentage of children cured at day 7), field workers will be particularly trained not to influence caregivers when they answer the questions "Is the child cured?" and insist that they simply answer by "Yes" or "No". If the answer is "No", the caregiver will be strongly advised to re-attend a HF, if possible, the one they went to for the initial consultation. Same procedure will be implemented if home visits are required using a REDCap mobile application. The questions included in the phone questionnaire are listed in **Appendix 1**.

During Phase 2, instead of a phone call, only caregivers of children participating to the sub-studies will receive a phone call or an automated SMS or automated phone call on day 7 with the same questions as in the questionnaire used in Phase 1. The automated follow-up system will be tested during Phase 1 in parallel with phone calls in a subset of patients, to check for congruency between the answers obtained by a caller and by automated voice message or SMS.

In Phases 1 and 2, when the participant reports that the family has no phone number, or no response is received from the phone call or SMS, the field assistant may make a physical follow up to ascertain the responses of several unanswered contact attempts with support of the local village leaders as key informants.

Data captured during the three cross-sectional quality of care surveys

Research clinical observers who have been trained on the study procedures will visit randomly selected HFs. They will ask HCWs present on that day for permission to observe a series of consecutive consultations. They will then ask the HCW to request oral permission from their patient and to sign a consent form indicating their own and the patient's consent to have an observer in the room. Observers will then record, using an electronic questionnaire, key symptoms (danger signs, main exposures and main symptoms) and signs (vital signs, main signs according to the child's main symptoms) assessed by HCWs, diagnostic tests requested, final decision on referral, final diagnoses retained, treatments prescribed and main information and advice given to caregivers. Observers will be trained not to interfere at all with the consultation process; only values of vital signs can be asked to the HCWs if necessary, to be recorded precisely by the observer.

8.1.8 Unique study identification number for each participant

Patients will be given a unique study identification number through a barcode or a QR code that will be placed into the patient paper medical record booklet. The ID identification labels will be pre-printed in bulk (specific to the district and HF) and delivered to the facilities. The patient will also be given a card with information on the study and additional barcode stickers. The sticker will be scanned using a barcode/QR code reader in the tablet each time the child presents at a HF. In the registration module on the tablet, an advanced search system will be created that uses the study identification number, the name of the child, the name of the mother or father or caregiver and the date of birth, so that the child can be identified with certainty and former consultations, if any, can be retrieved, and linked to the new consultation. The same code will also be used in order to link the laboratory results from surveillance

activities back to the child's consultation information.

8.2 Objective 2: Adapt the algorithm to epidemiological and demographic conditions

The algorithm will be improved in terms of diagnostic and prognostic accuracy and adapted to local fluctuations in epidemiology, demography, geography and resource availability. Each potential modification will first be evaluated by the Tanzanian clinical expert group for its clinical coherence, safety and potential benefit and then applied to the retrospective data. If these analyses confirm both a clinically relevant positive impact and estimate that there will be sufficient future cases during the study period to detect this improvement, the change in the algorithm will tested in a randomized sub-study using the same study design as in Phase 1, except that randomization will take place at patient level rather than HF level. If the positive impact is confirmed in the sub-study, the modification will be implemented in all relevant locations/patient sub-groups. If the retrospective analyses confirm a positive and potentially clinically relevant impact of a modification where resource savings is also expected but there will not be sufficient power to formally test the change during the study period, the modification will be implemented and monitored based on the related outcome indicators. If the impact of the modification achieves the expected results given the accumulated data at the end of the study period, it will be retained.

During the sub-studies, HCWs attending a child who could potentially benefit from this modification in the algorithm will be informed about the potential change in the algorithm. Either the modified branch or the original branch of the algorithm will then randomly appear on the screen to guide the HCW's decisions. Antimicrobial prescriptions and clinical cure, as well as all the other available and outcome measures relevant for the algorithm change, will be assessed the same way as in Phase 1 and compared between children managed with the modified and the original version of ePOCT+. If the benefit is confirmed by the sub-study, the new algorithm branch will be implemented for all relevant children in the relevant HFs. If the benefit is not confirmed, the original version of the algorithm will be used in all involved children.

If shown to be promising according to the analyses of the retrospective data and considered feasible to implement, we will be testing modifications in the algorithm with a focus on the aims described below. Other types of modifications might also be tested if considered important by the clinical experts.

Adapting the frequency of mRDT testing to malaria prevalence:

As recommended by WHO (WHO 2011), in geographical areas where the positivity rate of malaria RDT is normally or seasonally falls below 1% (based on the data gathered through the use of ePOCT+), we will modify the "fever" branch of the algorithm to target malaria testing on the patient population at risk of having malaria. To do so, we will use the combination of demographic (e.g. village where the child lives), exposure (e.g. recent travel to an endemic area) and clinical (e.g. no other documented infection) criteria shown in the data analyses to best predict the presence of malaria. In this specific geographical area, we will thus randomize (at patient level) febrile children to be managed either using the original algorithm "fever" branch (that recommends all children to be tested for malaria), or using the modified version of the "fever" branch (that targets testing to the children at risk for malaria). If we measure that the clinical cure rate is equivalent to that of children managed with the original "fever" branch, the later will be implemented for all children in that geographical area. In case the positivity rate would later increase in that area due to resurgence of malaria, the application would revert to the original "fever" branch until malaria prevalence goes down again.

This dynamic malaria testing strategy would reduce the number of malaria tests used (that would likely have been negative) and also increase the percentage of mRDTs that are positive, which will improve the motivation and trust of HCWs in malaria testing. This strategy has already been implemented in some areas or countries in Africa using malaria predictors described in the literature, but not yet based on individual patient local data as it will be done in the present study.

Improving the accuracy of pneumonia diagnosis:

The same study design as for malaria testing will be used to improve pneumonia diagnosis accuracy using a combination of clinical predictors or new diagnostic tools, and/or to save CRP rapid tests in children with cough or difficult breathing. The new “cough” branch of the algorithm will be implemented in all children either if the clinical cure rate with the new branch is expected to be better than that of children managed with the original “cough” branch OR if the number of CRP tests used is expected to decrease.

Assessing the utility of dengue rapid tests in outbreak conditions:

If the microbiological surveillance in a few sentinel sites (see chapter 8.3) identifies a dengue outbreak, a dengue rapid test will be added to the “unspecific fever” branch of the algorithm and tested using the same study design as above. The modified “unspecific fever” branch including dengue testing will be implemented in all children if the clinical cure rate with this new branch is better (i.e. >30% decrease in the clinical failure rate) than that of children managed with the original “unspecific fever” branch. As soon as the dengue outbreak is over, the “unspecific fever” branch will revert to the original version and the use of dengue rapid tests will be stopped.

Assessing the utility of typhoid rapid tests in high prevalence conditions:

If the prevalence of typhoid in febrile children is shown to be >10% in a certain area or time period (based on the microbiological surveillance data), using rapid tests, even with sub-optimal sensitivity and specificity as it is the case for now, could be beneficial from a clinical point of view. Indeed, with a pre-test probability of 10% and a positive likelihood ratio of 4 for the typhoid RDT, the post-test probability is 30%. The performance of the test can be enhanced by adding clinical predictors identified in previous studies in Tanzania (De Santis et al. 2017).

In this situation, a typhoid rapid test will be added to the “unspecific fever” branch of the algorithm and its clinical utility assessed using the same design as above. The new “unspecific fever” branch including the typhoid rapid test would be implemented in all children of the high prevalence area, if the clinical cure rate with the new branch is better (i.e. >30% decrease in the clinical failure rate) than that of the original branch. When typhoid prevalence decreases, the “unspecific fever” branch will revert to its original version.

Improving prediction of a severe clinical outcome:

If a combination of demographic, clinical and/or laboratory predictors, or a new POC test, seems to allow for better identification of children who will develop severe disease and thus be hospitalized secondarily or die, the benefit of adding an alert to HCWs in the algorithm, recommending them to refer the child to hospital, will be evaluated. If adding this alert allows to decrease mortality by at least 10%, it will be implemented for all children.

Adapting the algorithm to improve cure rates of children with chronic disease risk factors:

If specific predictors of clinical failure can be identified in children with chronic disease risk factors (e.g. malnourished, HIV positive children, children with sickle cell disease), the relevant branches of the algorithm will be modified for these children (e.g. an antibiotic treatment may be recommended in a child with a certain clinical constellation). This personalized algorithm will be implemented in all children with the chronic disease risk factor if there is a clinical benefit as described above.

8.3 Objective 3: Enhance disease surveillance and outbreak detection

All data generated by ePOCT+ will be sent to a central sever based at IHI. Data will be visualized through the medAL-outbreak dashboard made available to district health authorities using the key M&E and HMIS indicators they will have chosen. To enhance disease surveillance, targeted microbiological testing will complement the syndromic surveillance data generated automatically by ePOCT+ in HFs. This microbiological testing may take place during Phases 1 and 2 in 6 to 8 sentinel HFs (3 to 4 per site) on children presenting with at least one of three syndromes (significant respiratory disease, gastro-intestinal disease or non-specific fever). In response to the spatiotemporal analyses,

at a later stage of the surveillance study, we may expand surveillance sample collection beyond the sentinel sites using a mobile surveillance team. Rapid tests will be used on site and appropriate microbiological tests (PCR and serologies) will be performed at the research laboratories of NIMR-Mbeya and IHI-Ifakara/Bagamoyo once every other week.

To be able to perform these specific investigations for surveillance purposes, from which sick children will not benefit directly for the present acute episode (only indirectly when they attend for another episode and the algorithm has been improved in the meantime), caregivers of targeted children will be asked by the HCW to sign a separate written consent form allowing biological specimens to be collected. Caregivers and children will be asked if they agree to provide an additional blood, stool, urine or nasopharyngeal sample for research and surveillance purposes, and be provided a paper-based participant information and sample collection form providing sufficient information for caregivers to make an informed decision about their participation in this sub-study. All children participating in the surveillance sample collection will undergo the full written consent process (same as children participating in other sub-studies).

Thresholds for the detection of abnormalities in the data and for alerts for potential epidemics will be integrated in the medAL-outbreak system. Results will be discussed with the Epidemiology section of the MoH under the Directorate of Preventive Services (DPS), including the necessity for additional confirmatory tests at the Geneva Centre for Emerging Viral Diseases. This will allow health authorities to investigate if the cause of abnormal trends in the data is due to a real local epidemic or another reason. For example, a sudden increase in malaria cases could be due to a problem with the quality of the batch of malaria RDTs used. If the cause of the abnormality is not obvious, more in-depth investigations will be undertaken, such as performing clinical evaluations by an experienced physician or using pathogen rapid test (such as a Dengue or typhoid RDT) on site or other targeted lab tests at the research laboratory of IHI in Bagamoyo or NIMR in Mbeya (such as multiplex PCR with a tropical fever panel). These more in-depth data will also feed into the ML analyses and improve prediction capacity of children' clinical outcome. The clinical management algorithm will be modified temporarily, if necessary, according to the clinical experts.

8.3.1 Training and feedback of the CHMT on medAL-outbreak

After finalizing the list of health indicators with the person in charge of M&E and HMIS at the district health office, the medAL-outbreak dashboard will be installed on her or his PC. She or he will be trained on how to use the tool and interpret trends in the indicators for decision-making. Her or his feedback on the interface will be solicited and the dashboard will be modified, if necessary, to ensure optimal user experience. Regular discussion between the site investigator and the district team will take place throughout the study to modify or further improve the medAL-outbreak tool.

8.4 Objective 4: Enhance M&E and supportive supervision and mentorship

A similar process will be undertaken to ensure optimal use of the medAL-monitor tool. Individuals who normally conduct supervision visits to HFs will be trained on how to use and interpret the information displayed on the dashboards. HCWs and facility in-charges will also receive training on how to monitor their clinical practices at the individual and facility levels, perform self-audits, and build internal capacity and culture of quality improvement. User feedback will be solicited throughout the project and the interface improved.

8.5 Objective 5: Create a supportive environment for the use of clinical decision support algorithms and disease surveillance tools

8.5.1 Implementation research

During Phases 1 and 2, qualitative studies will be undertaken to assess facilitating factors and barriers to ePOCT+ deployment. HCWs, caregivers, community members and district health managers will be interviewed to assess the perception and acceptability of ePOCT+ and related tools.

Sustainability of the intervention will be evaluated by assessing the level of local human capacities that will have been reached at the end of the study in terms of: 1) clinical competencies by HCWs; 2) clinical algorithm creation by medical experts; 3) software and IT infrastructure maintenance by computer scientists; 4) disease surveillance and outbreak detection by health authorities and; 5) ML analysis by data scientists. These capacities will be enhanced by specific professional training programs and exchanges organized by the different institutions involved in the DYNAMIC project, as well as the University of Dar es Salaam for ML.

Finally, costing analyses will be performed using ML on the data collected throughout the study, assigning a cost to each procedure performed during consultations, as well as to transportation to the HF or to the hospitalization of the child. An ecological assessment of the intervention will also be performed to calculate the carbon footprint or carbon-saving of the intervention.

8.5.2 Stakeholder involvement and community sensitization

The objectives of the study and the way in which it will be conducted have already been discussed with the ICT and M&E Technical Working Group of the Ministry of Health in Tanzania and Child Health unit in the Reproductive and Child Health section of the Department of Preventive Services at the Ministry of Health. The ICT and M&E section of the President's Office Regional Administration and Local Government (PO-RALG) have been informed of the study and their support for the study obtained in principle.

In addition, the District Medical Officers and the CHMT members of the study districts in Ifakara TC, Kilombero DC and Mbeya DC have been contacted, informed of the study and provided support in the initial selection of study facilities by providing some information relevant for the study inclusion and exclusion process. Both have indicated willingness to support the study and work together once all approvals are obtained from respective authorities.

A Tanzanian stakeholder group will be established. It will contribute to the content of key documents and give feedback on the planned activities. If needed, international experts can be invited at certain time points. The stakeholders group might evolve along time but the initial composition will include Child health experts, Member/ leaders of the Pediatric association in Tanzania, Influential people (elders or religious), ICT/ ML experts in Tanzania, political leader at local communities, and CHMT or RHMT representative.

Members of the community will also be involved throughout the study. Meetings with community leaders will be held in convenient locations to encourage participation. In initial study introduction meetings, community leaders will be informed of the study and asked to disseminate the information to their communities. Subsequently, wider community meetings will be held to solicit feedback about the intervention throughout the study period. Lastly, focus group discussions will allow community members to formally participate in the study.

8.5.3 Development of frameworks for the future scale-up of the e-POCT+ intervention

During the last year of the DYNAMIC project, we will develop for the Tanzanian national and district health authorities, as the ultimate owners and users of ePOCT+ and its data, the necessary tools for further national scale-up. These tools will include but are not limited to:

- An optimized version of the software allowing health authorities to modify the e-algorithms without the help of software programmers whenever they need (MedAL-Creator). This software application will allow clinical experts to program, maintain and update any type of algorithm aimed at guiding HCWs during a patient consultation, whatever the age or medical condition. It will also include a feature to deploy or export a new version of the algorithm into all tablets located at the HFs. Through an intuitive interface, the user will be able to draw the medical algorithm by dragging and dropping some predefined elements such as exposures, signs, symptoms and diagnostic tests.
- A manual for local experts on the evidence-based path that needs to be followed to adapt and update the algorithm's medical content.
- A document on the best practices for the application of ML to clinical big data.
- To be able to ensure maintenance of the MedAL-Creator open-source software in the long term, Unisanté is presently considering creating a non-profit foundation linked to Unisanté that would provide services at a very low price for low-income countries or public entities, with the aim of reaching the largest possible number of patients with this public good.
- To the extent possible, interoperability guidelines will be developed for the prevailing eHealth tools with which ePOCT+ could be integrated. Examples include, but are not limited to, aggregation of patient level data from ePOCT+ for automated reporting into the DHIS2 platform, or linkage of ePOCT+ to the registration module of an electronic medical records system so that information from patient encounters managed using ePOCT+ can be stored in the patients' medical record.
- Support to health authorities willing to develop implementation guidelines that take into account the facilitators and barriers discovered during the project will be provided upon request.

9 LABORATORY PROCEDURES

9.1 Specimen collection and management

Trained HF routine staff will collect venous blood samples by standard venipuncture from the right or left antebrachium into pre-labeled vacutainer tubes containing EDTA and serum separator tubes. The total volume (for clinical management and surveillance) will not exceed 2.4 ml/kg per patient weight (maximum 15 ml), as recommended by KEMRI and WHO guidelines, of which a maximum of 1 ml/kg (maximum 6 ml) will be used for surveillance purposes (WHO 2013). Two nasopharyngeal samples will be collected by gently rubbing a sterile, synthetic swab on the patient's nasal mucosa for 2-5 seconds. For stool samples, the subject will be provided a pre-labeled container in which to collect the sample and instructions on how to collect uncontaminated samples.

All collected samples will be stored temporarily at the HF in a fridge and then transferred in batches in a cooler box with ice packs to the reference research laboratories for storage in an ultralow (-20ºC or -80ºC) freezer until analysis. In the two reference research laboratories in Mbeya and Bagamoyo, a testing strategy combining several levels of basic and advanced diagnostic tests, specific to the syndrome presented by the children, will be applied on the samples (that will be pooled whenever possible to save testing resources).

All Tanzanian and Swiss-based implementing partners/personnel handling specimens and involved in their transport will be trained prior to conducting the procedures. Training will be provided at each site by the laboratory coordinator and supported by site investigators under the supervision of the country coordinator. Only trained HF staff and project personnel will have access to the specimens. To address participant privacy and confidentiality, all biological samples will be labeled with a unique identification code assigned to each enrolled sampled child, and then logged in a register (sample log/map or database). Results of the surveillance laboratory investigations, which will be available 2

to 3 weeks after the initial consultation, will be communicated back to health workers by the research team from IHI or NIMR. Health workers will forward the results to the caretakers.

The majority of the laboratory analyses will be conducted at research laboratories in Tanzania at reference research laboratories in Mbeya or Ifakara in accordance with the procedures described below. In rare cases, Material Transfer Agreements (MTA) will be filled by all partners prior to any sample transfer from Tanzania to the Centre for Viral Emerging Diseases in Geneva, Switzerland. When shipping samples between Tanzania and the Centre for Emerging Viral Diseases in Geneva, the shipper will obtain all necessary documentation and permits from the appropriate regulatory authorities and follow all safety and International Air Transport Association (IATA) guidelines. The shipper will notify the approved couriers for transport in approved secured shippers and provide the recipient with the air bill/tracking number(s) and itemized shipping log.

Biobanks: All specimens will be securely stored in locked and temperature monitored ultralow freezers in Mbeya and Ifakara research laboratories for up to 15 years after project completion. The specimen database will be password-protected, and personal identifying information kept in a secure manner and maintained by the surveillance and information management team, and not share to a third part. Access will be restricted to project personnel only for surveillance data analysis.

9.2 Serology to characterize exposure to viral and/or bacterial pathogens

Commercial and established standardized serological assays for known agents, both bacterial and viral will be used to screen children with syndromes of interest. When appropriate, serological assays will be performed on-site, and for advanced characterization the samples will be analyzed at the reference research laboratories in Mbeya or Ifakara and at Geneva Centre for Emerging Viral Diseases.

9.3 Viral detection and discovery

Virus detection in collected samples will be performed using a combination of multiplex PCR such as BioFilm Array and advanced analyses such as high throughput sequencing (HTS) and full genome sequencing to detect known and discover novel viruses from different sample types. All personnel handling these samples will follow biosafety guidelines and perform viral nucleic acid extraction and molecular detection protocols in a required biosafety level facility at Mbeya or Ifakara. Virus targeted are of potential pandemic or epidemic significance and those for which public and preventive measures (i.e. vaccination, education) exist.

9.4 Molecular detection of bacterial pathogens

Appropriate microbiological tests and multiplex PCR targeting conserved genes will be used to identify the full range of bacterial pathogens and to identify new bacterial agents in samples from children with diseases of unknown origin where the viral infections have been ruled out.

10 STATISTICAL METHODS

10.1 Sample size calculations

Throughout the study, non-inferiority in clinical cure will be defined as a risk difference of <30% in the proportion of clinical failures while superiority in clinical cure will be defined as a decrease of ≥30% in the proportion of clinical failures. Superiority regarding resources (whenever medicines prescribed or diagnostic tests ordered by HCWs or time spent to perform a consultation) will be defined as a decreased need of ≥25%.

10.1.1 Cluster-randomized controlled study (Phase 1)

For the cluster-randomized study in Phase 1, the primary analysis will evaluate whether the use of ePOCT+ for case management of sick children in PHC facilities 1) results in similar clinical cure at day 7 (non-inferiority analysis), as compared to routine care and 2) decreased antibiotic prescriptions (superiority analysis) at the initial consultation. The sample size was calculated for testing the non-inferiority of the clinical cure outcome, which will require a much larger sample size due to the rareness of the event. In addition, the testing of differences in antibiotic prescription is conditional on satisfying non-inferiority in the clinical outcome. Assuming a failure rate of 3% in the control arm (corresponding to a 97% clinical success rate), based on previous studies, the acceptable non-inferiority margin will be a risk ratio of 1.3. This corresponds to the upper bound of the difference in failure rate not exceeding 0.9% if the control group failure rate is 3%. We utilized a fixed risk ratio rather than an absolute risk difference to protect against potential unexpected event rates (Head et al. 2012).

We assumed a cluster size of 900 patients and an intraclass correlation coefficient of 0.002 in the clinical outcome based on similar studies. To have 80% power to detect a risk ratio 1.3, for a one-sided hypothesis test at alpha of 0.025, we would require 19 clusters and 17,100 patients per arm (total patients n=37,620 assuming 10% loss to follow-up), rounded to a total of 40 HFs and 40'000 patients. However, due to uncertainty regarding the assumptions of the sample size calculation and to capture a wider more representative range of conditions, we will continue recruitment for Phase 1 for a minimum of 6 months. Due to lower enrollment than initially anticipated, an independent statistician will use the clinical failure outcome data from the control arm, and projected number of patients enrolled after all health facilities have enrolled patients for a minimum of 6 months to re-calculate the sample size, If the sample size recalculation does not have sufficient power to meet the study objectives, the study may be extended, or the number of health facilities increased. To avoid implementation bias, the clinical failure proportion in the control arm will not be shared to the study team.

10.1.2 Randomized sub-studies to test algorithm modifications (Phase 2)

Sample sizes for the randomized sub-studies aimed at evaluating the benefit of a modification in an algorithm branch will be calculated following the same general principles as for Phase 1. Consideration of the desired or acceptable difference in clinical cure rate will be determined according to the type of intervention applied. This acceptable difference cannot be decided prior to knowing what intervention will be implemented, and on which population or subpopulation this intervention will be applied. For example, a small improvement in the cure rate from 90% to 93% in a certain sub-population of patients will be considered beneficial if not too demanding in terms of resources to be added. The same considerations will be applied for interventions aiming at improving the rational use of medicines (e.g. antibiotics or antimalarials). The highest sample size between that required for assessing the clinical outcome and that for the resource saving outcome will be used.

10.2 Estimated number of beneficiaries

In Phase 1, the number of direct beneficiaries include children in the intervention arm of the study (n=17,100) and HCWs using ePOCT+ (n=75) for a total of 17,175 people. Indirect beneficiaries may include caregivers and immediate family members who are less exposed to infectious diseases as a result of better cure rates of their child/sibling. Using an average rural household size of 5 people according to the 2004-2005 Demographic and Health Survey, the number of expected indirect beneficiaries is approximately 68,400 (i.e. 4*17,100, excluding the sick child who is a direct beneficiary).

In Phase 2, we expect that an average of 200 children per month per HF will be managed using ePOCT+, which corresponds to around 500,000 consultations over 2 years in the 100 HFs. The number of direct beneficiaries will be less than this, because children may come to the facility more than once, either for the same illness or a different illness over the 2-year study period. In Phase 2,

approximately 300 HCWs are expected to directly benefit from using ePOCT+ and their clinical decision support and e-learning functions. Additionally, approximately 20 users of medAL-monitor and 30 users of medAL-outbreak at the district and national levels will also benefit directly. Lastly, all individuals who are capacitated IT systems management, ML, adaptation of the algorithm through MedAL-Creator, etc. will be included as direct beneficiaries (n=50). Because of high coverage of the intervention in phase 2, indirect beneficiaries will be the entire population of the intervention districts (n=1,053,000) as a result of 1) less exposure to infectious diseases due to better cure rates of sick children; 2) reduced drug pressure and likelihood of antibiotic resistance due to reduced antibiotic prescriptions; and 3) reduced risk of illness due to more effective outbreak detection and response.

10.3 Statistical analyses for the primary outcome measures in the cluster randomized controlled study of Phase 1

Analyses will follow CONSORT guidelines (Schulz et al. 2010, Piaggio et al. 2012) and intention-to-treat (ITT) principles, that is including all participants as randomized. A flowchart will describe the inclusion and follow-up of participants by study arm. Baseline characteristics will be described by study arm with summary statistics such as median and interquartile range or number and percentage; no formal testing between arms will be performed (Pocock et al. 2002). Outcomes will be described by arm using summary statistics. For the non-inferiority comparison between ePOCT+ and the control arm for clinical cure, a CI approach will be used. A figure illustrating the CIs and the non-inferiority margin will be presented. Primary analyses for the non-inferiority comparison will be performed on both the per protocol and ITT sets (Committee for Proprietary Medicinal P 2001). If ePOCT+ is found to be non-inferior to the control in terms of clinical cure rate, then we will assess for superiority using the ITT set. If non-inferiority is established, we will test for significant differences in the co-primary outcome of antibiotic prescription rate. For this outcome, the percentage of patients receiving antibiotics at day 0 will be assessed using multi-level logistic regression model regression with the cluster included as a random effect. Binary secondary outcomes will be evaluated in the same way. All estimates will be reported with 95% confidence intervals (CI).

Effect modification of the primary outcome of antibiotic prescription of the primary endpoints will be performed in groups of patients where the highest reduction in antibiotic prescription is expected, and areas of extension of the content of the algorithm compared to the version tested in the previous ePOCT study. Effect modification by sex, age groups (young infants ages 1 to 59 days, children aged 2 months to 5 years, and children aged 5 to 14 years), respiratory symptoms, fever without clinical source, gastrointestinal complaints (vomiting and diarrhea), skin problems, ear and throat problems, anemia, and malnutrition will be assessed by incorporating an interaction between arm and the respective variable, acknowledging that power will be low. All models will be adjusted for clustering and the stratification factors (Kahan et al. 2012). Primary analyses will be complete case; we may adjust for further baseline variables which are associated with missing outcome data (which is analogous to performing multiple imputation in the case of a single endpoint). Further details will be provided in the statistical analysis plan.

10.4 Machine learning analyses

Machine learning (ML) is a specialized branch of statistics that creates updatable algorithms, which learn from evolving data in real time. Phase 2 of this project will make use of a specially designed algorithm-building platform, that will help clinicians explore and visualize patterns in the data collected by ePOCT+ during phase 1. Two main types of ML methods will be used: 1) Supervised classification and correlation and 2) anomaly detection. The former aims to create algorithms that predict the value of a labeled feature (e.g. the result of a malaria rapid test) using alternative data. Anomaly detection, on the other hand aims to find unusual clusters of values for a certain place, time or person, and are the basis of outbreak detection and discovering erroneous data inputs that could be used to train field workers.

10.4.1 Supervised classification and correlation

Using the insights gleaned from visualizing the data, the expert clinician group will lead the generation of clinically supervised modifications to the static algorithms in ePOCT+ that aim to 1) reduce resource consumption (e.g. predicting the results of expensive or unavailable diagnostic tests), 2) improve or personalize diagnostic accuracy (e.g. creating algorithms adapted to local epidemiology, or better identifying patients who had severe outcomes in phase 1) or 3) improve the completeness of data collected by asking questions that would better predict the values that are missing. For this, the algorithm building platform will allow clinicians to explore various predictive methods, ranging from logistic regression, neural networks and random forests. They will control the inputs required to make the prediction and decide at what threshold the predicted result will impact care decisions. The algorithm will then be updated with accumulating data to best reflect its dynamic environment.

10.4.2 Anomaly detection

Various methods allow us to identify and visualize clusters of abnormal data, by highlighting these instances, clinicians and epidemiologists can train the algorithms by labeling which are of interest and what, if any, actions should be taken when they are detected. Various methods will be explored for this aim and adapted to the type of anomaly under investigation (for example unsupervised density-based approaches such as isolation forests, and k-nearest neighbors [KNN] or multidimensional non-linear kernel-based techniques such as self-organizing maps [SOM] and support vector clustering [SVC]). The clusters deemed interesting will then be investigated for gold-standard labeling by clinicians and the surveillance team and visualizations may be exported to users of the tool for training purposes (medAL-monitor).

10.5 Analyses of data from operational research studies

Quantitative data from cross-sectional quality of care surveys, provider clinical competency or knowledge assessments, and caregiver and patient interviews will be analyzed using conventional statistical methods to compare means (t-tests or paired t-tests) or frequencies (chi-square tests) across intervention groups or time periods. Regression analysis may also be used to explore associations of multiple predictors with outcomes of interest.

All interviews and focus-group discussions will be audio-recorded with the participant’s informed consent. Detailed notes will be taken during the interviews. Records will be manually transcribed in its original language (Kiswahili) and translated into English. A qualitative data analysis software (e.g. Nvivo) will be used to code the English interview transcripts in order to identify emerging themes. A predefined list of codes based on the interview guide and the recurrent themes highlighted in the detailed notes will be used. New codes will be created when new themes are identified during more detailed analysis of the transcripts. Deductive coding will be used to extract the themes based on the interview guide, but inductive coding will also be used to extract any unexpected themes that arise during the interviews.

11 INFORMATION TECHNOLOGY, DATA MANAGEMENT AND MONITORING

11.1 Overall IT solution

The overall IT solution is provided by several components; some of them have been already discussed or presented previously, the diagram (**Figure 10**) below is the complete overview of the solution. From left to right we have the following components:

MedAL-Creator: The clinical algorithm management system that will allow to manage, reference, modify and deploy the different versions of the algorithm into a mobile device. MedAL-Creator is hosted in Switzerland and managed by the IT Unisanté team.

ePOCT+: The application (tablet or PC) that will contain the algorithm and be used for data acquisition by the HCW. The ePOCT+ could be deployed in a standalone or multi-device mode. In a standalone mode the application will receive updates of the algorithm directly from MedAL-Creator.

Local Data: The application that enables ePOCT+ to work in a multi-device environment. It plays a role of central database at the HF level. This software will receive the updates of the algorithm from MedAL-Creator.

Data Management System: this component will be considered as the data hub of our solution. Hosted on a server at the Dar es Salaam head office of IHI:

- MedAL-Data: This is the data collector for the country, it plays several roles:
    - Data collector
    - Data manager (Clean, Quality Check, Audit trail, Anonymization)
    - Data publisher
    - Data monitoring
- medAL-outbreak: Indicators for disease surveillance and early epidemic warning and response will be decided with and provided to CHMTs through a dedicated web-based application
- medAL-monitor: Targeted supervision to HFs by the CHMT will be organized based on the quality of care indicators calculated in real time from the uploaded data and made available through a web-based dashboard
- Research Database: This is a database only with anonymized data where the machine learning team will do their analyses.

Figure 10: IT architecture.

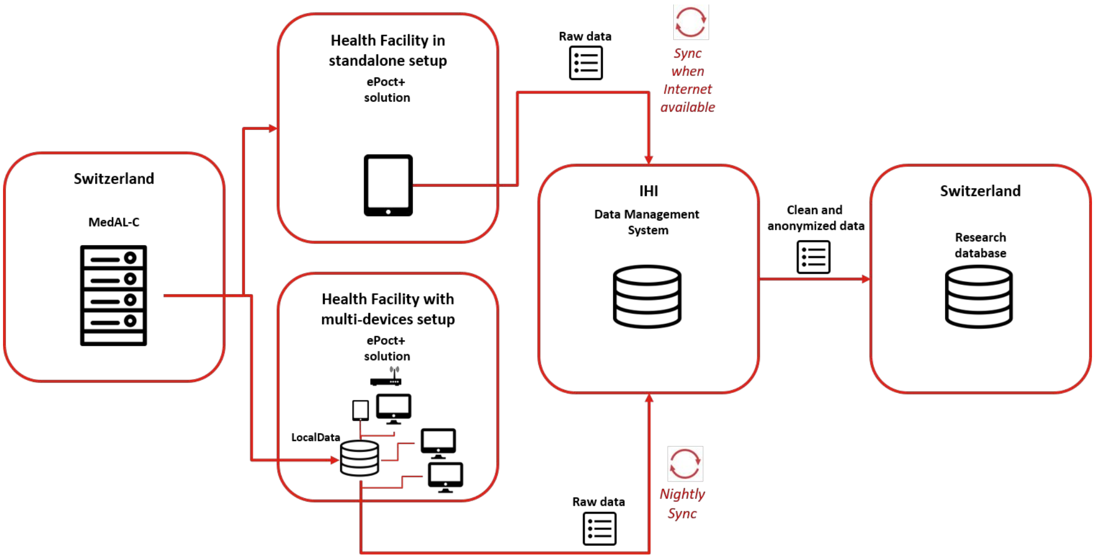

11.2 Data flow

The data flow is shown in **Figure 11**. Data collection starts at the HF where the study clinicians capture the data using ePOCT+. When an Internet connection is available ePOCT+ (or Local Data in multi-devices set-up) will sync the data with the data management system hosted at IHI. The data management team at IHI will monitor, clean and anonymize the data in this system and make the

anonymized data available for the Unisanté IT team. The Unisanté IT team will monitor the data received, communicate the possible errors or anomalies before sending them to the EPFL ML team for ML-based statistics.

Figure 11: Data flow.

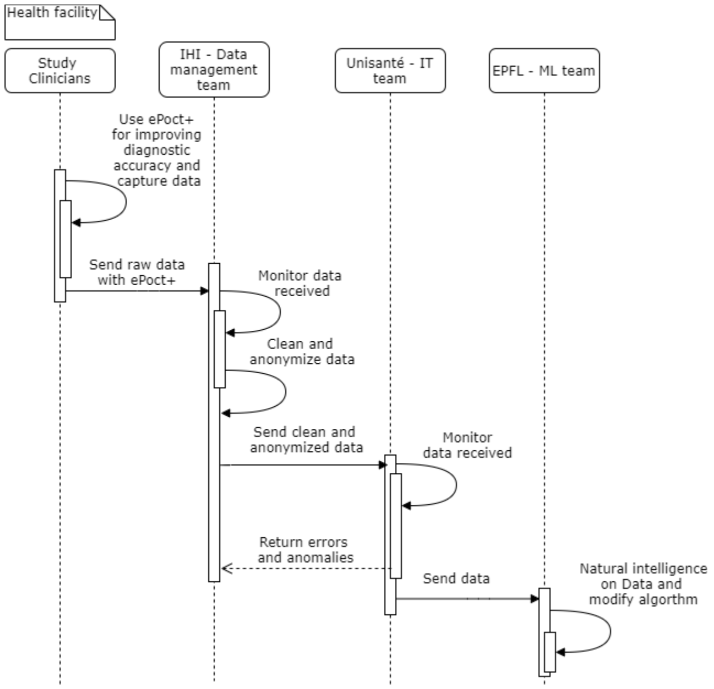

11.3 Data security and privacy

IHI (where all the data will be stored) working with Unisanté, in collaboration with the local health management teams, will be responsible for ensuring the privacy and security of all data collected during the project, especially with respect to individual patient data. Our methods are designed to comply with international and Tanzanian human rights standards that apply to regulating patient data protection, ownership, storage and processing.

Primary data collection devices:

Tablets used for primary data collection will be password-protected and assigned to individual HFs and/or HCWs. A secure PIN will be necessary on each request to view an existing patient file. When not in use, the tablets will be locked and stored in physically secure location.

Transfer of data to secure local servers:

All data uploaded from the primary data collection devices to the local server at IHI in Tanzania will be encrypted using an SSL connection. All raw data will remain on the secure local servers in Tanzania and will not be exported as such.

Anonymization and machine learning IPD privacy:

Before being transmitted to Switzerland for machine learning analysis or the creation of reporting metrics, automated anonymization algorithms will remove identifying features of the raw data. Features removed will include names, dates of birth, addresses and identification numbers. At no point will these confidential data attributes be exported outside of the secure local server.

Transfer of data from local to Swiss servers:

When transferred from Tanzania to Unisanté in Switzerland, the data will travel through a secured VPN connection. Data Transfer Agreements (DTA) will be filled by all partners prior to any data transfer.

Additionally, machine learning analyses will explore new techniques of identifying additional feature combinations that put patient privacy at risk. We will also investigate means to export elements of the data in a manner that renders it impervious to the latest re-identification attacks, for instance, by adding carefully calibrated noise (differential privacy) or by advanced encryption. Finally, we will also explore the possibility of decentralized learning that will allow analyses to take place without data transmission.

11.4 Data quality monitoring

In order to minimize data recording errors by HCWs on the tablet, ePOCT+ data fields will include validations that limit the values to plausible ranges. Cleaning and quality assessment of the anonymized raw data arriving on the server at IHI will first be performed by the data management team using predefined algorithms developed in collaboration with the Swiss data management team. Queries related to data captured through day 7 phone calls will be brought to and solved directly with the research assistants performing the calls. Systematic missing or inconsistent data coming from HFs will be discussed first with the CHMT of the corresponding district and then with the person in-charge of the HF, if necessary. A quarterly data review meeting will be organized by the study team along with a representative of CHMTs of each district to discuss any outstanding data quality issues.

Regular quality checks will also be performed on the cleaned anonymized data sent to the Unisanté server by the Swiss team and queries sent to and solved with the IHI data management team if necessary. The database will be locked after all data has been verified and all raised queries have been resolved. Conventional and ML analyses will then start to be performed on this locked database.

11.5 Monitoring of study procedures and major health system barriers

All data related to child health or quality of the consultation, such as compliance with the algorithm by HCWs, are outcomes and indicators that will be monitored by HF teams and the CHMT using the medAL-monitor tool, and thus not directly by the study team. Indeed, it is essential that the later does not interfere with decisions taken by HCWs, persons in charge of HFs or district teams.

Regarding study procedures, monitoring will be delegated to the monitoring units of IHI and NIMR, which will function as independent monitors. In addition, the study team will perform routine on-site and off-site internal data quality and monitoring activities. The main goal of monitoring will be to assure

data quality, and the wellbeing of study participants. The study monitoring team will visit research assistants performing the day 7 phone calls once a month during Phase 1. The aim is to ensure that they attempt to reach caregivers who did not respond to the first call at least twice more, and that they do not influence caregivers in their responses regarding the child clinical outcome. The study monitoring team will visit each HF once shortly after the on-site face-to-face training to check if the intervention tools are in place and used for the purpose for which they have been designed (e.g. that informed consent is obtained correctly, or that ePOCT+ is used during the consultation and that data are not entered retrospectively after the consultation is finished) and that there are no major issues impeding basic clinical procedures (e.g. stock out of antimalarials or antibiotics for weeks). The study team will also have regular meetings with the district teams to discuss major problems related to the study, the intervention, or the tools (that may jeopardize the study) and propose solutions if any. The study team will accompany the CHMT during some of their supervision visits to monitor how they use the medAL-monitor tool and how HCW are able to perform self-auditing. A detailed monitoring plan will be developed before the start of the study.

12 ETHICAL CONSIDERATIONS

12.1 Participant information and consent

Inclusion of patients in the intervention study, as well as caregivers, HCWs, community members or CHMT members in the mixed-methods studies, will occur only if the participant has provided written informed consent. Written informed consent will be obtained prior to data collection, all non-routine clinical assessments, and any other study-related activities (except in the routine implementation of ePOCT+ in Phase 2 where the consent process will be simplified, see **Section 8.1.1**).

District and HF consent will also be obtained for the intervention. Information and consenting tasks for sick children and their caregivers may be performed by any HF staff (for more details on the informed consent procedure, see **Section 8.1.1**). Information and consenting tasks related to mixed-methods studies will be performed by the research scientists and their study team members. All these individuals will undergo adequate training with a special emphasis on the process of unbiased information of study participants ensuring that non-participation in the study does not result in a child not being adequately managed. During this training, theoretical and practical sessions will be devoted to research ethics and informed consent procedures.

The participant information sheet/informed consent documents will be available in Kiswahili. These documents, as well as their translated versions in English, are attached to the present protocol (see **Appendix 3**).

12.2 Risk-benefit assessment

Patients and caregivers:

The proposed intervention is based on international and national treatment guidelines, supplemented by POC tests that are routinely used for managing sick children at hospitals. As such, we do not anticipate that the implementation of this intervention will pose a risk to patients. We rather anticipate that the implementation of ePOCT+ will provide a direct benefit to patients by improving case management of sick children at PHC level. With reduced antibiotic prescriptions, we anticipate children to develop healthier immune systems, making them less likely to be sick in the future. Additional benefit to patients and their caregivers is the reduced cost of healthcare due to fewer reattendance visits and secondary admissions.

Community:

Communities in the intervention area are also expected to benefit from the project in several ways.

Reduced antibiotic prescriptions will reduce antimicrobial drug pressure in the community, and hence the risk of drug resistance. Better cure rates are expected to lessen the exposure to infectious diseases in the community. Enhanced disease surveillance and outbreak detection is also expected to decrease infectious disease transmission.

Health care workers:

HCWs are expected to develop better clinical skills and practices through the use of ePOCT+ and the associated training and mentorship provided through the project. The data generated with ePOCT+ will produce an opportunity for their work to also be audited by their supervisors and the CHMT. In some circumstances, this could pose some perceived or actual risk to their job security. All data available to higher managers or authorities will be de-identified to mitigate these risks as much as possible.

District health authorities:

The CHMT will be able to monitor the quality of the consultations performed by HCWs and plan better for their supervision and mentorship visits to HFs. To ensure that this insight into their daily work is not perceived by HCWs as external control but rather a support mechanism for them to improve their competencies and skills, they will have access to their own data and will be encouraged to perform self-auditing before the supervision visit by the CHMT, so that a fruitful and respectful discussion can take place during the visit. This strategy has already been shown to be very well accepted by HCWs in Burkina Faso during the implementation of a CDSA in routine conditions at primary care level (Bessat et al. 2019).

Central health system:

The cluster randomized controlled study will generate evidence about the impact of ePOCT+ under routine (versus experimental) conditions. This will support the development of guidelines for potential national scale-up of the intervention, and similar digital health tools, in the future. The economic evaluation will provide additional information on the overall costs of healthcare provision with and without the use of ePOCT+. Lower costs of medicines are anticipated, which equates to lower costs of healthcare provision at the central level because under-5 children are provided health services free of charge in Tanzania.

The project overall:

The project provides many benefits, as described above, but also comes with some risks. For example, clinicians may be hesitant to use the tool, because it is difficult to use or they feel it is too prescriptive, etc. As CDSAs cannot cover all possible clinical situations, even in a well-defined patient target group, HCWs are ultimately responsible for the clinical management of all patients and may override the recommendations of the algorithm and provide additional or withhold treatment according to their own clinical judgement. Additionally, qualitative research will be conducted to solicit user feedback and facilitate improvements to ensure that the ePOCT+ application meets user needs. Furthermore, the application may be perceived by some stakeholders as a “black box” and be wary of the use of machine learning for algorithm adaptation, which may reduce their trust in the system. To ensure full transparency, as requested more and more by stakeholders (Ansermino et al. 2019), the medical content of the algorithm used during Phase 1, as well as any modification introduced during Phase 2, even if applied only in some geographical areas or for a short period of time, will be published in real-time on the website dedicated to the DYNAMIC study. Health authorities and HCWs will thus always have access to this content and know the exact combinations of clinical predictors used to suspect or diagnose each disease. No change in the algorithm will take place automatically and all modifications will be introduced manually. ML will be used only in the background, to complement conventional statistical methods to analyze data and guide decisions of the clinical expert group. For safety reasons, ML will never be used within the algorithm. Lastly, in Phase 2 of the study, there will be no control group. There is a possibility that due to circumstances that are external to the

project (e.g. severe weather events, food insecurity, disease outbreaks) childhood morbidity and mortality could increase. In order not to incorrectly attribute poor outcomes to the study, we will need to monitor these events as closely as possible.

12.3 Premature termination of study

The Principal Investigators may decide to terminate the study prematurely according to certain circumstances, such as ethical concerns that cannot be resolved, in case of safety of the participants being compromized (e.g. if the benefits no longer outweigh the risks), alterations in accepted clinical practice that make the continuation of the study unwise, or evidence of harm of the intervention. In case of premature study termination, the National Health Research Ethics Committee at NIMR will be notified as per local guidelines.

12.4 Local regulations, declaration of Helsinki and protocol amendments

The research project will be carried out in accordance with the research plan outlined in this protocol, principles enunciated in the current version of the Declaration of Helsinki, Essentials of Good Epidemiological Practice issued by Public Health Switzerland (EGEP), and all national, legal, and regulatory requirements as applicable. The DYNAMIC study will be registered on ClinicalTrials.gov.

All protocol modifications will be documented. A protocol amendment can be initiated by either the Sponsor or any Investigator. The Investigator will provide the reasons for the proposed amendment in writing and will discuss with the Sponsor and the Swiss and Tanzanian Principal Investigators. Any protocol amendment must be approved and signed by the Sponsor and the Principal Investigator and will be submitted to the National Health Research Ethics Committee at NIMR for information and approval. Approval will be obtained before any changes can be implemented, except for changes necessary to eliminate an immediate hazard to study participants, or when the change involves only logistical or administrative aspects of the study. An annual report by the National investigators will be submitted to the National Health Research Ethics Committee at NIMR per national guidelines.

12.5 Alignment of the project with regional and/or governmental strategies

Our project is perfectly aligned with the Tanzania Digital Health Investment Roadmap 2017-23, proposing provision of innovative software, IT infrastructure and skills to enable client tracking and monitoring to support quality health care. Investments are required in data systems to strengthen the ability of health authorities to make evidence-based decisions. The project is also in line with the National Action Plan on Antimicrobial Resistance 2017-22 that advocates for strengthening stewardship for antimicrobial use in HFs by providing evidence-based prescribing and dispensing guidelines, as well as monitoring and evaluating the use and consumption of antibiotics at all levels. Our project also supports the National Malaria Strategic Plan 2014-20, which calls for introducing evidence-based, innovative diagnostic tools for malaria detection and differential diagnosis of other pathogens causing febrile illnesses. The disease surveillance component also supports the National Action Plan for Health Security 2017-2021 that aims at strengthening and sustaining the capacity of Tanzania to promptly detect and confirm outbreaks, the frequency and intensity of which may increase in the coming years due to climate change.

13 ORGANIZATION AND TIMELINES

13.1 Organizational structure

Unisanté is the leading institution in close collaboration with the IHI, the Mbeya branch of NIMR-MMRC, the Swiss TPH, Basel Switzerland and the Machine learning and optimization laboratory of EPFL, Lausanne Switzerland.

The Project Technical Committee (PTC) will ensure an appropriate framework to achieve project objectives, take necessary decisions to effectively implement the workplan and achieve its deliverables, by allocating funds and resources according to activities and milestones. The implementation team, lead by two site coordinators, will ensure implementation of all activities in the field in the Mbeya and Morogoro regions, with the support of the different technical teams lead by one team member from and based in Tanzania, seconded by one team member from Switzerland.

The Project Management team will coordinate and monitor activities against the implementation plan; negotiate and administer funds; organize meetings and teleconferences; coordinate report writing; ensure effective communications between partners; monitor milestones and deliverables; and coordinate the dissemination of results obtained by the project to the scientific community (see

Figure 12).

The National Stakeholders Group will review and discuss progress regarding of ePOCT+ implementation, discuss, review and give feedback on the annual project reports, provide guidance on aligning activities with national priorities, call upon harmonized specialised technical assistance in key areas when necessary, identify, discuss and propose solutions for possible problems related to the intervention and/or the health system.

Figure 12: Organizational structure for the project

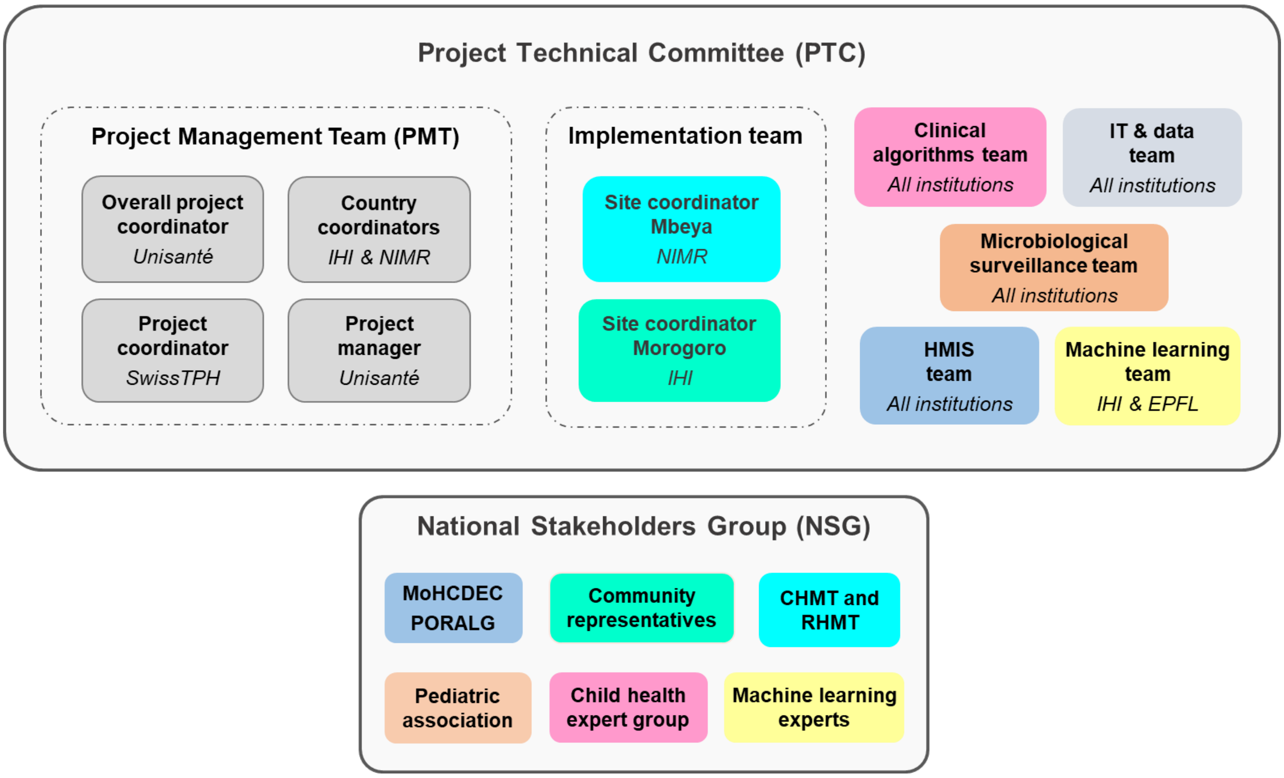

13.2 Roles of investigators and collaborators

Unisanté will act as the main sponsor for the study. The principal investigators and co-investigators at IHI, NIMR, Unisanté, Swiss TPH and EPFL, are responsible for the overall design and conduct of the study. The Tanzanian principal and co-principal investigators at IHI and NIMR will oversee the study conduct in Tanzania. This includes assuring that the study is conducted in accordance with the

protocol, the rights, safety and welfare of the study participants are protected, local requirements of the ethical committee are adhered to, adequate training of site personnel and integrity of study data are maintained. The team from Switzerland (Unisanté and Swiss TPH) will provide epidemiological, statistical, IT, administrative and financial management support. The Geneva Centre for Emerging Viral Diseases in Switzerland will provide technical support to the research laboratories in Mbeya and Ifakara for the microbiological surveillance.

Prof. Valérie D'Acremont will serve as the overall scientific lead and the key contact for study design and writing of the protocol. She will lead the process of documenting findings and lessons learnt through the project. She will oversee the development and writing of scientific manuscripts, as well as technical and financial reports. She will lead writing of progress reports and dissemination of results to partners and stakeholders.

Dr. Honorati Masanja and Dr. Nyanda Ntinginya will be responsible for conduct of research activities according to ethical guidelines. They will contribute to the development of the research protocol and serve as main contacts for ethics committees and the MoHCDEC. They will contribute to the dissemination of results to partners and stakeholders.

Dr. Kristina Keitel will provide key technical input on the study design of the ePOCT+ cluster-randomized study. She will contribute to the analysis of the results of the study, technical and scientific manuscripts, and dissemination to partners and stakeholders. Dr. Keitel will provide pediatric expertise to the clinical team responsible for approving the initial content of the clinical algorithm, as well as the major modifications taking place during the study. She will also support knowledge transfer of the development and adaptation of clinical decision support algorithms to Tanzanian stakeholders.

Dr. Mary-Anne Hartley will lead the machine learning analyses, with the support of Prof. Martin Jaggi, as well as the related preparation of progress reports, technical and scientific manuscripts, and dissemination to partners and stakeholders. They will also organize knowledge transfer to professionals in Tanzania willing and able to learn the use of applied machine learning for clinical interventions in resource-limited settings.

Dr. Chacha Mangu will oversee all the research field activities in Mbeya. He will also participate in the coordination of activities related to disease surveillance and outbreak detection. He will contribute to the writing of progress reports, scientific manuscripts and dissemination of results to partners.

Dr. Alexandra Kulinkina will be the overall project coordinator who will coordinate activities between the different groups involved in the study in Switzerland, as well as with the Tanzania research team. She will support the PI in all her activities and coordinate the development and writing of scientific and technical manuscripts, as well as the writing of progress and financial reports and dissemination of results to partners and stakeholders.

Dr. Lameck Luwanda will lead the implementation of the ePOCT+ tool at HF level and the medAL-outbreak tool at district level in the Morogoro Region. He will contribute to the development and expert approval of the ePOCT+ algorithms, writing of progress reports, scientific manuscripts and dissemination of results to partners.

Dr. Godfrey Kavishe will lead the implementation of the ePOCT+ tool at HF level and the medAL-outbreak tool at district level in the Mbeya Region. He will contribute to the writing of progress reports, scientific manuscripts and dissemination of results to partners.

Dr. Rainer Tan will be responsible for providing technical support to all activities required to implement the ePOCT+ tool at HF level and the medAL-outbreak tool at district level in the Mbeya and Morogoro Regions. He will lead the development and approval of the ePOCT+ algorithms by the medical experts and coordinate the monitoring the implementation of ePOCT+ in HFs. He will contribute to the writing of progress reports, scientific manuscripts and dissemination of results to partners.

Charles Festo will coordinate the National IT teams. He will make sure that the infrastructure for the data collection is operational, secure, maintained and backed up (at 2 sites). He will also coordinate the daily operations (data management incl. data cleaning, back-up, etc.)

Vincent Faivre will lead the development and maintenance of the software and assess the IT infrastructure needed with the local IT team. His team will also develop the necessary tools for the data collection and management. During the data collection, he will also coordinate data verification, in close collaboration with the IT and data management teams of IHI and NIMR. The IHI team will in particular be responsible for initial data cleaning and anonymization of the raw data stored on the server based at IHI.

Peter Agrea (NIMR) and Ibrahim Evans Mtebene (IHI) will help develop and test the data collectors ("Local Data" at the facility level, "Main Data" at the National level), in close collaboration with the Swiss and Mbeya IT teams. He will also provide support and training to the users and to daily operations (data management incl. data cleaning, backup, etc.) He will also be involved in the medAL-outbreak and medAL-monitor dashboards and assess the feasibility of the integration with the Government of Tanzania's Hospital Management Information System (GoT-Homis).

Geofrey Isdory Ashery will participate to the qualitative studies and contribute to the writing of progress reports, scientific manuscripts and dissemination of results to partners.

Happy Mkali will lead the biological and laboratory aspects of disease surveillance and outbreak detection in the field and contribute to the writing of progress reports, scientific manuscripts and dissemination of results to partners.

Prof. Isabella Eckerle will coordinate the disease surveillance and outbreak detection component, in collaboration with the Tanzanian partners, as well as the related preparation of progress reports, technical and scientific manuscripts, and dissemination to partners and stakeholders. She will in particular be responsible for the laboratory analyses and interpretation of results on emerging viral diseases at the Geneva Centre for Viral Emerging Diseases.

Dr. Lisa Crump will give technical inputs and field support to the disease surveillance and outbreak detection component, in particular related to zoonotic and bacterial diseases, in collaboration with the Tanzanian partners. She will participate to the related preparation of progress reports, technical and scientific manuscripts, and dissemination to partners and stakeholders.

Alan Vonlanthen will ensure project coordination and communication between the different teams (clinical, data & IT, ML and project management) involved in the project.

Dr. Tracy Glass will serve as the lead statistician of the ePOCT+ cluster-randomized controlled study of Phase 1.

Dr. Gillian Levine will support the clinical team with developing and validating the clinical content of the young infant algorithms. She will assist with analyzing the data and contribute to scientific manuscripts.

Dr. Sabine Renggli will support the Morogoro site team with the implementation of the project activities. She will assist with writing of progress reports, scientific manuscripts and dissemination of results to partners and stakeholders.

Martin Norris will support the project management and IT teams with coordination of the technical activities related to the electronic platform. He will also assist the clinical team with trouble-shooting and verifying changes in clinical content and its translation in the electronic tool.

Humphrey Mhagama will support the study implementation in the Mbeya site as a study nurse.

Dr. Alix Miauton will will be responsible for providing technical support to all activities required to implement the ePOCT+ tool and associated algorithm adaptations in Phase 2 of the study. She will contribute to the writing of progress reports, scientific manuscripts and dissemination of results to partners.

The Sponsors, Investigators, and key study members as outlined above have all contributed to the writing of the present protocol. They may delegate tasks and responsibilities as appropriate but will provide sufficient oversight over all tasks delegated and will ensure that each individual, to whom a

task is delegated, is qualified by virtue of education, training, and experience to perform each of their delegated tasks.

13.3 Timelines

The DYNAMIC project started on the 1st April 2019 and will cover a five-year period up to 31st March 2024. Phase 1 (including the pilot phase) will last from January 2021 to March 2021, phase 2 from April 2022 to October 2023, implementation research from January 2021 to March 2023, assessment of sustainability from January 2023 to March 2024 and diseases surveillance from October 2021 to March 2023. A project timeline is shown in **Figure 13**.

Figure 13: Project timeline

	2020			2021				2022				2023				2024
	Q2	Q3	Q4	Q1	Q2	Q3	Q4	Q1	Q2	Q3	Q4	Q1	Q2	Q3	Q4	Q1
**Project phase**				Pilot		Phase 1			Phase 2							
**Tools implementation**																
Sensitisation meetings			X			X										
HCWs in person training			X			X										
Onsite face-to-face training			X			X										
e-learning platform								X	X	X	X	X	X	X		
ePOCT+ provided to intervention HFs						X	X	X	X	X	X	X	X	X		
ePOCT+ provided to former control and additional HFs									X	X	X	X	X	X		
Modifications of ePOCT+									X	X	X	X	X	X		
medAL-monitor							X	X	X	X	X	X	X	X		
medAL-outbreak							X	X	X	X	X	X	X	X		
**Data collection**																
Consultation data				X	X	X	X	X	X	X	X	X	X	X		
Follow-up data				X	X	X	X	X	X	X	X	X	X	X		
Cross-sectional surveys				X				X				X				
Samples analysed at IHI & NIMR laboratories							X	X	X	X	X	X				
Qualitative studies							X	X	X	X	X					
**Data analysis**																
Clinical data					X	X	X	X	X	X	X	X	X	X	X	
Laboratory data							X	X	X	X	X	X				
Qualitative data							X	X	X	X	X					
**Dissemination**																
Publications and reports						X		X	X					X	X	X
Dissemination of findings								X	X			X	X	X	X	X
Framework for scale-up												X	X	X	X	X

14 CAPACITY BUILDING, KNOWLEDGE TRANSFER AND DISSEMINATION PLANS

The proposed project offers many opportunities for capacity building and transfer of technology, which are summarized in **Table 4**.

A website dedicated to the DYNAMIC project will be set up to publish the medical algorithm and its modifications in real time. Regular news on the status of the project, teaching material and tutorials developed for the project and preliminary results of studies will also be made available on this website.

As soon as they are available, the final results of the cluster randomized controlled study of Phase 1 will be presented to and discussed with the CHMT as well as with the staff of all HFs participating in the study. They will also be presented and discussed with the communities of the catchment areas of these HFs. The same will be done again with results of studies in Phase 2. National dissemination meetings will be held to share the gained knowledge among all stakeholders in Tanzania. Reports for stakeholders and funders will be submitted annually and/or upon reaching key milestones in the project.

Results from this project will be published in open-source peer reviewed scientific journals and presented at relevant conferences and during symposia at national and international level, thus contributing to the dissemination of knowledge gained for the benefit of stakeholders worldwide involved in child health, digital health, disease surveillance and the use of ML in the health sector.

Table 4: Areas of anticipated capacity building and technology transfer by health system level.

**Area of capacity building/knowledge transfer**	**Health system level**		
	**National**	**District**	**Health Facility**
Clinical knowledge	Evidence-based clinical guideline development and continuous update of clinical content through the involvement of key pediatric stakeholders in the development of the ePOCT+ content<br>Increased capacity of Tanzanian MDs in clinical algorithm validation and translation into health policy through PhD program	Increase in medical knowledge through support in supervision visits by study personnel<br>Supervision at HF level through the development and implementation of medAL-monitor	Improved clinical skills of HCWs through the use of ePOCT+ and e-learning modules
Information technology	Set-up and maintenance of IT infrastructure through continuous technical support from study team	Set-up and maintenance of IT infrastructure through initial capacity building and continuous technical support from study team	
Software development and maintenance	Development and maintenance of ePOCT+ software through involvement of Tanzanian software engineers	Maintenance of software at the district level through continuous capacity building of the district IT team throughout the project	Knowledge in the use of electronic tools for patient management
Database set-up and management	Set-up and maintenance of the database		

Data interpretation and use for decision-making	Definition of relevant indicators through ownership of database by MoH and technical support	Management and interpretation of health surveillance data, interpretation of dashboards through technical support by study team	Improved ability of HCWs to interpret data due to information provided through medAL-monitor
Machine learning	Use of machine learning to analyze health data through involvement of Tanzanian partners in machine learning activities and a training program		

15 BUDGET

The budget for the DYNAMIC project administered from Tanzania is 3,011,800 Swiss francs (CHF), which is equivalent to approximately 7,150,000 Tanzanian Shillings. **Table** breaks down the budget by project year (1st April 2019 through 31st March 2024) and project activity. The collaborating institutions each have their own budget, with a total of 1,600,000 CHF allocated to IHI and 1,400,000 CHF allocated to NIMR, inclusive of 10% institutional overhead charges. The budget includes the planning year (Year 1), which ends on 31-Mar-2020. It is displayed in gray color because no study activities were undertaken.

Table 5: Total budget in Swiss francs.

In CHF (Swiss francs)	**Year 1**	**Year 2**	**Year 3**	**Year 4**	**Year 5**	**Total**
Staffing	180,000	280,000	280,000	280,000	180,000	1,200,000
Training and workshops	8,000	25,000	25,000	15,000	15,000	88,000
IT infrastructure and equipment	15,000	70,000	70,000	15,000	10,000	180,000
ePOCT+ implementation (Phases 1 and 2)	15,000	70,000	60,000	50,000	15,000	210,000
medAL-monitor/outbreak implementation	-	10,000	20,000	30,000	10,000	70,000
Disease surveillance	-	100,000	300,000	300,000	100,000	800,000
Mixed methods operational research	5,000	20,000	25,000	15,000	5,000	75,000
Stakeholder meetings and dissemination	5,000	10,000	10,000	5,000	10,000	40,000
Administrative costs	10,000	25,000	25,000	10,000	10,000	82,000
Overhead (10%)	23,800	61,000	81,500	72,000	35,500	273,800
**TOTAL**						**3,011,800**

Budget justification

Staffing costs include salaries of all permanent IHI and NIMR employees who are fully or partially allocated to the DYNAMIC project, as well as any temporary staff hired specifically for the project.

Training and workshop costs include rental fees for meeting venues, development and printing of all training materials, and per diems/allowances for meeting participants.

IT infrastructure and equipment costs include the procurement of all tablets, computers, servers, routers, and other items required to establish connectivity between tablets in study facilities and with

the main server located at IHI. This category also incudes the set up and maintenance of the call center in Phase 1 and SMS system in Phase 2.

Transportation costs include hiring or purchase of vehicles, motorbikes, and other means of transportation between the study sites and/or to all the HFs for data collection or monitoring associated with all study activities. Driver salaries are also included in this category.

ePOCT+ implementation costs include the procurement of POC lab tests and medicines (if needed), printing of labels for identifying patients, salary top-ups for HF staff that perform additional duties for the study, internet bundles (if needed), and other miscellaneous expenses.

medAL-monitor/outbreak implementation costs include meetings and consultations with the users and relevant stakeholders regarding the content and visualization of the data, regular visits to district health officials to build capacity in interpreting the dashboards, and facilitating regular district visits to HFs for mentorship and supervision of the HCWs.

Disease surveillance costs include all specimen collection, storage, transport, and laboratory analysis costs. Also included in this category is potential hiring of laboratory staff and/or training existing HCWs on the procedures specific to the surveillance activities.

Mixed methods operational research costs include the development of finalization of study tools, the costs of field data collection, software for data analysis, and the hiring of temporary field enumerators.

Stakeholder meetings and dissemination costs include rental fees for meeting venues, refreshments, development and printing of materials, reimbursement of transportation costs or per diems/allowances for meeting participants (if needed). Also included in this category are publication fees and development of dissemination materials at the end of the study (e.g. pamphlets, booklets, etc.).

Administrative costs include office space rental fees, office supplies, fees for various approvals, and other miscellaneous expenses.

16 REFERENCES

- Asermino JM Wiens M, Kissoon N. Evidence and Transparency are Needed to Develop a Frontline Health Worker mHealth Assessment Platform. Am. J. Trop. Med. Hyg., 101(4), 2019, p. 948.
- Benoun, J. M., J. C. Labuda, and S. J. McSorley. 2016. 'Collateral Damage: Detrimental Effect of Antibiotics on the Development of Protective Immune Memory', MBio, 2016: 7.
- Bessat C, Zonon NA, D'Acremont V. Large-scale implementation of electronic Integrated Management of Childhood Illness (eIMCI) at the primary care level in Burkina Faso: a qualitative study on health worker perception of its medical content, usability and impact on antibiotic prescription and resistance. BMC Public Health. 2019 Apr 29;19(1):449.
- Committee for Proprietary Medicinal P. Points to consider on switching between superiority and noninferiority. Br J Clin Pharmacol. 2001;52(3):223-8.
- Cordey S, Laubscher F, Hartley M-A, Junier T, Pérez-Rodriguez FJ, Keitel K, Vieille G, Samaka J, Mlaganile T, Kagoro F, Boillat-Blanco N, Mbarack Z, Docquier M, Tapparel C, Kaiser K , D'Acremont V. Detection of Dicistroviruses RNA in Blood of Febrile Tanzanian Children. *Emerg Microbes Infect*. 2019, 8(1):613-623.
- Cordey S, Hartley MA, Keitel K, Laubscher F, Brito F, Junier T, Kagoro F, Samaka J, Masimba J, Said Z, Temba H, Mlaganile T, Docquier M, Fellay J, Kaiser L, D'Acremont V. Detection of novel astroviruses MLB1 and MLB2 in the sera of febrile Tanzanian children. *Emerg Microbes Infect*. 2018; 7(1):27.
- D'Acremont V, Kilowoko M, Kyungu E, Philipina S, Sangu W, Kahama-Maro J, Lengeler C, Cherpillod P, Kaiser L, and Genton B.'Beyond Malaria — Causes of Fever in Outpatient Tanzanian Children', *NEJM* 2014; 370: 809-17.
- De Santis O, Kilowoko M, Kyungu E, Sangu W, Cherpillod P, Kaiser L, Genton B, D'Acremont V. Predictive value of clinical and laboratory features for the main febrile diseases in children living in Tanzania: A prospective observational study. *PLoS One* 2017;12(5):e0173314.
- Erdman LK, D'Acremont V, Hayford K, Rajwans N, Kilowoko M, Kyungu E, Hongoa P, Alamo L, Streiner DL, Genton B, Kain KC. Biomarkers of Host Response Predict Primary End-Point Radiological Pneumonia in Tanzanian Children with Clinical Pneumonia: A Prospective Cohort Study. *PLoS One* 2015; 10(9): e0137592.
- Farnham A, Utzinger J, Kulinkina AV, Winkler MS. Using district health information to monitor sustainable development. Bull World Health Organ. 2020 Jan 1;98(1):69-71. https://www.who.int/bulletin/volumes/98/1/19-239970/en/
- Fink G, D'Acremont V, Leslie HH, Cohen J. Antibiotic exposure among children younger than 5 years in low-income and middle-income countries: a cross-sectional study of nationally representative facility-based and household-based surveys. *Lancet Infect Dis.* 2019 Dec 13.
- Frischknecht R., Jungbluth N., Althaus H.-J., Doka G., Heck T., Hellweg S., Hischier R., Nemecek T., Rebitzer G., Spielmann M., Wernet G. Overview and Methodology. ecoinvent report No. 1. Swiss Centre for Life Cycle Inventories, Dübendorf, 2007.
- Hansoti B, Jenson A, Keefe D, De Ramirez SS, Anest T, Twomey M, Lobner K, Kelen G, Wallis L. Reliability and validity of pediatric triage tools evaluated in Low resource settings: a systematic review. BMC Pediatr. 2017 Jan 26;17(1):37.
- Hansoti B, Jenson A, Kironji AG, Katz J, Levin S, Rothman R, Kelen GD, Wallis LA. SCREEN: A simple layperson administered screening algorithm in low resource international settings significantly reduces waiting time for critically ill children in primary healthcare clinics. PLoS One. 2017 Aug 29;12(8):e0183520.
- Head SJ, Kaul S, Bogers AJ, Kappetein AP. Non-inferiority study design: lessons to be learned from cardiovascular trials. Eur Heart J. 2012 Jun;33(11):1318-24.
- Holmes AH, Moore LS, Sundsfjord A, Steinbakk M, Regmi S, Karkey A, Guerin PJ, Piddock LJ. Understanding the mechanisms and drivers of antimicrobial resistance. Lancet. 2016 Jan 9;387(10014):176-87.
- Hopkins H, Cairns ME, Chandler CIR, Leurent B, Ansah EK, Baiden F, Baltzell KA, Björkman A, Burchett HED, Clarke SE, DiLiberto D, Elfving K, Goodman C, Hansen KS, Kachur SP, Lalloo DG, Leslie T,

Magnussen P, Jefferies M, Mårtensson A, Mayan I, Mbonye AK, Msellem MI, OE, Owusu-Agyei S, Reyburn H, Rowland MW, Shakely D, Vestergaard LS, Webster J, Wiseman VL, Yeung S, Schellenberg D, Staedke SG, Whitty CJM. Impact of introduction of rapid diagnostic tests for malaria on antibiotic prescribing: analysis of observational and randomised studies in public and private healthcare settings. BMJ 2017;356:j1054.

- International Organization for Standardization (ISO). Environmental management - Life cycle assessment – Principles and framework. ISO 14040: 2006A; Second Edition 2006-06, Geneva.
- International Organization for Standardization (ISO). Environmental management - Life cycle assessment – Requirements and guidelines. ISO 14044: 2006B; First edition 2006-07-01, Geneva.
- Jolliet O, Müller-Wenk R, Bare J, Brent A, Goedkoop M, Heijungs R, Itsubo N, Peña C, Pennington D, Potting J, Rebitzer G, Stewart M, Udo de Haes H and Weidema B. The LCIA Midpoint-damage Framework of the UNEP/SETAC Life Cycle Initiative, Int J LCA 2004: 9 (6) 394 – 404.
- Kahan BC, Morris TP. Reporting and analysis of trials using stratified randomisation in leading medical journals: review and reanalysis. BMJ. 2012;345:e5840.
- Keitel K, Kilowoko M, Kyungu E, Genton B, D'Acremont V. Performance of prediction rules and guidelines in detecting serious bacterial infections among Tanzanian febrile children. *BMC Infect Dis* 2019; 19:769.
- Keitel K, Samaka J, Masimba J, Temba H, Said Z, Kagoro F, Mlaganile T, Sangu W, Genton B, D'Acremont V. Safety and efficacy of C-reactive protein-guided antibiotic use to treat acute respiratory infections in Tanzanian children: a planned subgroup analysis of a randomized, controlled non-inferiority trial evaluating a novel electronic clinical decision algorithm (ePOCT). *Clin Infect Dis.* 2019; 69(11):1926-1934.
- Keitel K, D'Acremont V. Electronic Clinical Decision Algorithms for the integrated primary care management of febrile children in low resource settings - Review of existing tools. *Clin Microbiol Infect*. 2018, 24(8):845-855.
- Keitel K, Kagoro F, Samaka J, Masimba J, Said Z, Temba H, Mlaganile T, Sangu W, Rambaud-Althaus C, Gervaix A, Genton B, D'Acremont V. A novel electronic algorithm using host biomarker point-of-care-tests for the management of febrile illnesses in Tanzanian children (e-POCT): A randomized, controlled non-inferiority trial. *Plos Medicine* 2017;14(10):e1002411.
- Lange S, Mwisongo A, Mæstad O. Why don't clinicians adhere more consistently to guidelines for the Integrated Management of Childhood Illness (IMCI)? Soc Sci Med. 2014;104:56-63.
- Mghamba, J., Mboera, L., Krekamoo, W., Senkoro, K., Rumisha, S., Shayo, E., & Mmbuji,. Challenges of implementing an integrated disease surveillance and response strategy using the current health management information system in Tanzania. P. J. T. J. o. H. R. 2004; 6(2), 57-63
- Nantanda, Rebecca, James K. Tumwine, Grace Ndeezi, and Marianne S. Ostergaard. 'Asthma and pneumonia among children less than five years with acute respiratory symptoms in Mulago Hospital, Uganda: Evidence of under-diagnosis of asthma', PLoS ONE 2013, 8: 1-9.
- Piaggio G, Elbourne DR, Pocock SJ, Evans SJ, Altman DG, Group C. Reporting of noninferiority and equivalence randomized trials: extension of the CONSORT 2010 statement. JAMA. 2012;308(24):2594-604.
- Pocock SJ, Assmann SE, Enos LE, Kasten LE. Subgroup analysis, covariate adjustment and baseline comparisons in clinical trial reporting: current practice and problems. Stat Med. 2002;21(19):2917-30.
- Rambaud-Althaus, Clotilde, Fabrice Althaus, Blaise Genton, and Valérie D'Acremont. 'Clinical features for diagnosis of pneumonia in children younger than 5 years: a systematic review and meta-analysis.', The Lancet. Infectious diseases, 2015; 15: 439-50.
- Rambaud-Althaus C, Shao S, Samaka S, Swai N, Perri S, Kahama-Maro J, Mitchell M, D'Acremont V, and Genton B. 'Performance of Health Workers Using an Electronic Algorithm for the Management of Childhood Illness in Tanzania: A Pilot Implementation Study.' *Am J Trop Med Hyg* 2017; 96: 249-57.
- Ryan, S. J., McNally, A., Johnson, L. R., Mordecai, E. A., Ben-Horin, T., Paaijmans, K., Lafferty, K. Mapping Physiological Suitability Limits for Malaria in Africa Under Climate Change. Vector-Borne And Zoonotic Diseases. 2015; 15 (12): 718-725
- Schulz KF, Altman DG, Moher D, Group C. CONSORT. Statement: updated guidelines for reporting parallel group randomised trials. Trials. 2010;11:32.

- Shao AF, Rambaud-Althaus C, Samaka J, Faustine AF, Perri-Moore S, Swai N, Kahama-Maro J, Mitchell M, Genton B, D'Acremont V. New Algorithm for Managing Childhood Illness Using Mobile Technology (ALMANACH): A Controlled Non-Inferiority Study on Clinical Outcome and Antibiotic Use in Tanzania. *PLoS One* 2015A; 10(7): e0132316.
- Shao A, Rambaud-Althaus C, Swai N, Kahama-Maro J, Genton B, D'Acremont V, and Pfeiffer C. 'Can smartphones and tablets improve the management of childhood illness in Tanzania? A qualitative study from a primary health care worker's perspective.', BMC Health Services Research 2015B; 15: 135.
- USAID. The Demographic and Health Surveys Program. https://dhsprogram.com/What-We-Do/Survey-Types/SPA-Questionnaires.cfm
- Walker, P. G., M. T. White, J. T. Griffin, A. Reynolds, N. M. Ferguson, and A. C. Ghani. 'Malaria morbidity and mortality in Ebola-affected countries caused by decreased health-care capacity, and the potential effect of mitigation strategies: a modelling analysis', Lancet Infect Dis 2015; 15: 825-32.
- World Health Organization 2006. 'Improving health worker performance:in search of promising practice'. https://www.who.int/hrh/resources/improving_hw_performance.pdf
- World Health Organization 2011. Interagency 'Universal access to malaria diagnostic testing: an operational manual'. WHO, Geneva, page 24. http://www.who.int/malaria/publications/atoz/9789241502092/en/index.html
- World Health Organization 2013. Howie S. Blood sample volumes in child health research: review of safe limits. http://www.who.int/bulletin/volumes/89/1/10-080010/en/#
- World Health Organization 2014A. 'IMCI Chart Booklet'. https://www.who.int/maternal_child_adolescent/documents/IMCI_chartbooklet/en/
- World Health Organization 2014B. "Ground zero in Guinea: the Ebola outbreak smoulders – undetected – for more than 3 months". http://www.who.int/csr/disease/ebola/ebola-6-months/guinea/en/
- World Health Organization 2018. Climate change and health: Key facts. Geneva, Feb 1. https://www.who.int/news-room/fact-sheets/detail/climate-change-and-health
- Yukich J, D'Acremont V, Kahama J, Swai N, Lengeler C. Cost savings with rapid diagnostic tests for malaria in low-transmission areas: evidence from Dar es Salaam, Tanzania*. Am J Trop Med Hyg.* 2010; 83(1): 61-8.

17 APPENDICES

Appendix 1: Case Report Forms

Appendix 2: Mixed-methods operational research tools

Appendix 3: Informed Consents
