# Supplementary material for: A cluster randomized trial assessing the effect of a digital health algorithm on quality of care in Tanzania (DYNAMIC study)
Source: PLOS Digit Health. 2024 Dec 23;3(12):e0000694. doi: 10.1371/journal.pdig.0000694 (PMC11666054; doi:10.1371/journal.pdig.0000694)
Supplement: S2 File — (PDF) [file pdig.0000694.s003.pdf]

*In accordance with PLOS's copyright policy, the following logos were taken out: DYNAMIC study, Ifakara Health Institute, Unisanté, Swiss Tropical and Public Health Institute, Mbeya Medical Research Center - National Institute of Medical Research

Statistical Analysis Plan for the Dynamic Study Ancillary Cross-Sectional Cluster RCT analysis:

Impact on antibiotic stewardship and quality of care for the management of sick children at primary health care facilities in Tanzania when using ePOCT+, a clinical decision support algorithm: insights from a cross-sectional cluster RCT ancillary study.

**Trial registration number**	NCT05144763		
**SAP version number**	1.1	Date	03 May 2023
**Protocol version**	This document has been written based on information contained in the study protocol version 5.0, dated 7 Sep 2022		

Authors	Position	Signature	Date
Godfrey Kavishe	Research physician	[signature]	21 August 2023
Marie-Annick Le Pogam	Senior research scientist / Methodologist	[signature]	21 Sept 2023
Lameck Luwanda	Morogoro site investigator	[signature]	21 August 2023
Alexandra Kulinkina	Project coordinator	[signature]	20 Sept 2023
Valérie D'Acremont	Principal investigator	[signature]	
Rainer Tan	Clinical coordinator	[signature]	22 August 2023

Contributing collaborators / authors

Authors	Position	Authors	Position
Chacha Mangu	Mbeya site investigator	Nyanda N. Elias	National Co-principal Investigator
Sabine Renggli	Senior research scientist	Honorati Masanja	National Co-principal investigator

Revision history

Protocol version	Updated SAP version no.	Section number changed	Description of and reason for change	Date changed
5.0	1.1	1.2	Only one primary outcome (assessment of principal IMCI symptoms and signs), instead of two co-primary outcomes in order for the message of the study to be clearer	03 May 2023
5.0	1.1	4.1.2	Removed secondary outcome 2: Mean score of all other symptoms and signs as observed by the clinical observer as they will be assessed as individual symptoms and signs	03 May 2023
5.0	1.1	4.1.2	Combined secondary outcomes 7 and 8 to formulate outcome 2.3; To determine the concordance between the diagnoses and antimicrobrials prescribed as they are both under secondary objective 2.3	03 May 2023
5.0	1.1	4.1.2	Removed secondary outcome 11: To evaluate the proportion of cases managed using ePOCT+ in intervention health facilities as it will be assessed in the descriptive analysis	03 May 2023
5.0	1.1	4.2	Cluster-level analysis instead of individual-level analysis as recommended when including less than 15 clusters per arm	03 May 2023

Table of Contents

1. Introduction: 3
1.1 Background and rationale 3
1.2 Objectives 3
2.0 Methods: 4
2.1 Study design 4
2.2 Intervention: 5
2.3 Randomization and sampling of health facilities 5
2.4 Sampling of consultations 5
2.5 Sample size 5
3.0 Study population 6
3.1 Eligibility 6
3.1.1 Health facility (Cluster) eligibility 6
3.1.2 Study Population Eligibility 6
3.2 Baseline characteristics 7
4.0 Main analyses 7
4.1 Outcome definitions 7
4.2 Analysis methods 10
References: 13

1. Introduction:

1.1 Background and rationale

The Integrated Management of Childhood Illness (IMCI) booklet for managing sick children under five years of age has been found to improve the quality of care and reduce childhood mortality(1,2) However, challenges with IMCI remain notably poor adherence to guidelines and excessive prescription of antibiotics(3–6). Furthermore, the IMCI guidelines are not comprehensive, causing uncertainty in managing illnesses not covered by IMCI and exacerbating the over-prescription of antibiotics even more (7).
Clinical decision support algorithms (CDSAs) have been found to improve adherence to IMCI and reduce antibiotic prescription without resulting in inferior clinical outcomes (8–10) However, most healthcare quality assessments were conducted in the context of efficacy studies, not effectiveness studies, so these interventions' effect is unclear in more real-life settings.

In this ancillary study of a cluster randomized controlled trial, we will use the methodology adapted from the Service Provision Assessment (SPA) to evaluate the impact of ePOCT+ (an electronic CDSA) on antibiotic prescription and quality of care in primary health care facilities in Tanzania as compared to routine practice. We will do this by conducting clinical observations of consultations with children aged two months to five years.

Within ePOCT+ a clinician can accept a recommendation for diagnosis, treatment, and management but may decide not to follow this recommendation in reality. The observation by external clinical observers of what clinicians do in reality will give us a better insight into the actual impact on the quality of care

1.2 Objectives

Primary objective:
The primary study objective is:

Obj 1 : To evaluate whether the use of ePOCT+ by clinicians in the management of sick children results in an increase (compared to routine care) in the proportion of the IMCI symptoms and signs assessed, and the IMCI counselling provided

Secondary objectives:
The secondary objectives are to compare health facilities using ePOCT+ [intervention], with health facilities not using ePOCT+ [control]) in terms of:

Obj 2.1 : Diagnoses made by clinicians
Obj 2.2 : Type, dosage, and mode of administration of antibiotics and antimalarials
Obj 2.3: Concordance between the diagnoses and antimicrobials prescribed (or not)

Obj 2.4: Children for whom screening for priority conditions was performed (e.g., TB, HIV, malnutrition, vaccination status) and who were subsequently referred to a specialized program

Obj 2.5: Concordance between the proposed antibiotic treatments by ePOCT+, the accepted antibiotic treatment by the clinician, and the actual treatment prescribed

Obj 2.6 : Overall antibiotic prescription

2.0 Methods:

2.1 Study design

The ePOCT+ intervention will first be trialed in 40 health facilities split equally between intervention and control arms as part of a cluster randomized controlled study.

A cross-sectional survey will be performed in a random subset of 18 of the 40 health facilities: nine randomly selected facilities from the intervention arm and nine from the control arm of the parent study (figure 1).This survey will be conducted two to four months after the start of the parent trial.

This survey will consist of observing consultations in the consultation room for children aged 2-59 months, and noting whether the selected symptoms, signs, and questions were requested by the healthcare staff or described spontaneously by the patient or caregiver.

Figure 1: Study design

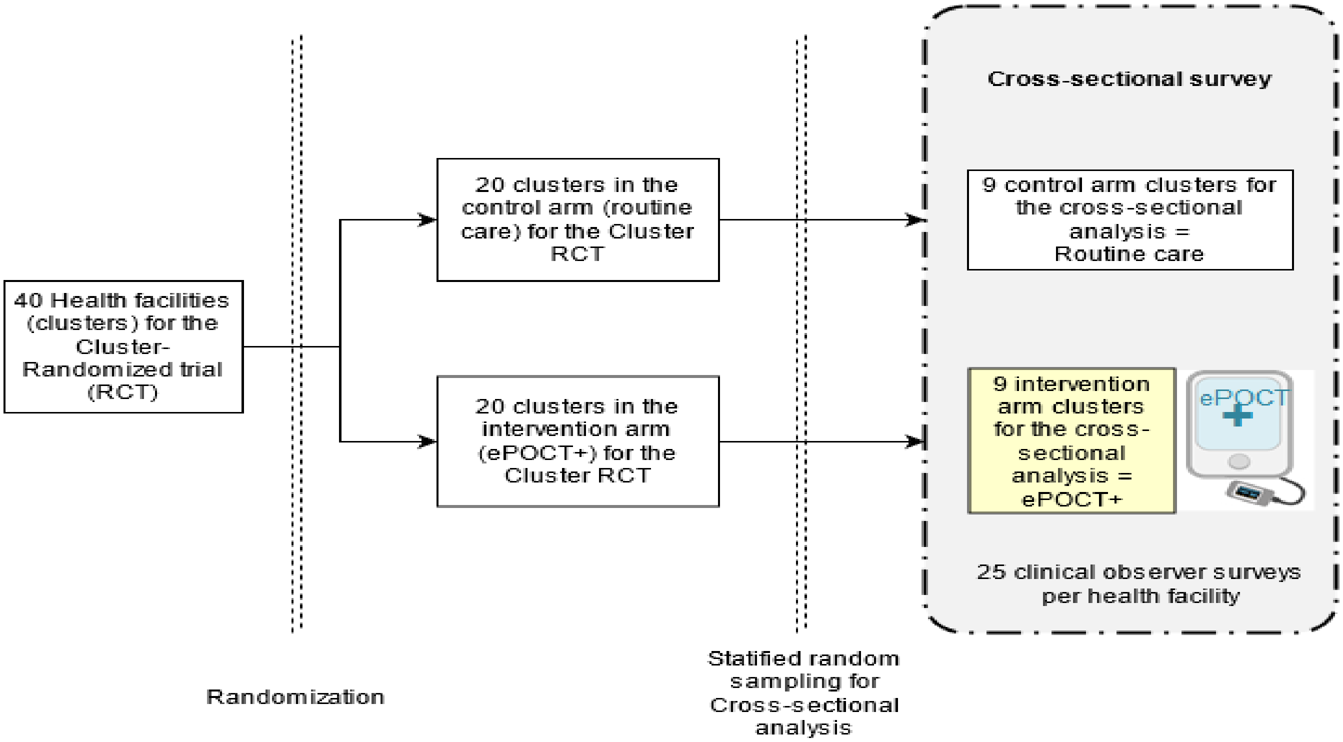

2.2 Intervention:
Provision of ePOCT+, an electronic clinical decision support algorithm on an android tablet, along with associated point of care tests (CRP, Hemoglobin, pulse oximetry), training, and mentorship. ePOCT+ prompts the health care worker to answer questions about demographics, symptoms, signs, and tests. Based on the answers, ePOCT+ will propose one or several diagnoses, treatments, and management plans, including the need for referral.

2.3 Control:

Half the facilities will have been randomized into control facilities for which health care workers will perform their tasks as per routine care.

The healthcare staff from the control health facilities will receive equivalent clinical training as the intervention health facilities, without any specific training on the intervention tool (ePOCT+).

2.4 Randomization and sampling of health facilities
Randomization of health facilities is described in the main trial statistical analysis plan. In summary, eligible health facilities were randomized on a 1:1 ratio using the following stratification factors: monthly attendance, type of health facility (dispensary or health centre), region and district.

An independent statistician will randomly select the 18 health facilities for the cross-sectional analyses among the 40 included in the main trial. All 8 health centers will be included in order to have sufficient information on both types of health facilities. Among the remaining 32 dispensaries, the 10 dispensaries will be randomly sampled and stratified by intervention arm (5 intervention, 5 control), and by region following the same 3:2 ratio as sampling for the main study (6 from Morogoro, 4 from Mbeya).

2.5 Sampling of consultations
A convenience sample will be used, following all eligible consultations when the clinical observer is present at the health facility during normal working days (Monday to Friday).

2.6 Sample size
The sample size calculation is based on the co-primary outcome of antibiotic prescription. A sample size of 25 patients in 9 clusters (i.e. health facilities) per arm will have 80% power to detect a 25% absolute decrease in mean antibiotic prescription from a baseline of 50%, using an intraclass correlation coefficient (ICC) of 0.10 and an alpha of 0.05. The ICC was based on studies evaluating prescription variations among different health care facilities/practices, ranging from 0.07 to 0.10 (11–14).

We expect a high variability in baseline values of symptoms and signs assessed by a clinician and between clinicians(4,8,15,16) Based on the above sample size (9 clusters per arm, 25 patients per cluster), we would have 67-93% power to detect a 30% absolute increase in the assessment of primary IMCI symptoms and signs, considering a baseline value of 40-60%, an ICC of 0.15 – 0.25, and an alpha of 0.05.

The sample size was calculated using the “clustersampsi” command in Stata 16.0.

3.0 Study population

3.1 Eligibility

3.1.1 Health facility (Cluster) eligibility

Health facility eligibility for the cluster RCT sub-study is the same as the parent Dynamic study. It is based on the health facility meeting all the inclusion criteria and none of the exclusion.

Inclusion criteria:

- Government and private government-designated primary care HFs (dispensaries and health centers).
- Located in the Mlimba, Ifakara and Ulanga DC (Morogoro Region) or in Mbeya DC and CC (Mbeya Region).
- Seeing at least 20 children aged 2 months to 5 years per week on average over the past 6 months (to facilitate participant recruitment).

Exclusion Criteria:

- Secondary and Tertiary HFs (district, regional, zonal and specialized hospitals).

3.1.2 Study Participant Eligibility

Patient eligibility is the same as the main study; however, the included age group will be restricted to 2-59 months and only primary consultations (not follow-up consultations). A patient is eligible for the study if he/she meets all the inclusion and none of the exclusion criteria.

Inclusion criteria:

- Age 2 months to 59 months
- Presenting for the first time for an acute medical or surgical condition at this health facility
- Agreed to participate in the main study

Exclusion Criteria:

- Presenting for a follow-up consultation for an acute illness that was already previously assessed in the past 14 days at this health facility
- Presenting for scheduled consultation for a chronic disease (e.g. HIV, TB, NCD, malnutrition)
- Presenting for routine preventive care (e.g. growth monitoring, vitamin supplementation, deworming, and vaccination).
- Caregiver unavailable, unable or unwilling to provide informed consent

Exclusion criteria specific to the intervention arm:

- Health care worker seeing the patient was not trained to use the ePOCT+ tool in the intervention arm.

- Participants seeing a health care worker on a day where the ePOCT+ tool is not functioning the day of observation due to IT issues (updates, crash) which would not allow the health care worker the option to use ePOCT+
- In case the healthcare worker was trained on ePOCT+ and that the tool is functioning, but he/she decides not to use the tool, the observation will still be included.

3.2 Baseline characteristics

The baseline cluster (health facility) characteristics that will be summarized by study arm include:

- Number of health facilities by type (dispensary or health center) and region
- Average number of patients seen per month by health facility
- Availability and readiness of basic health care services score based on the Tanzanian Service Provision Assessment (median and IQR)
- Demographics of healthcare workers including IMCI training

The baseline patient and caregiver characteristics that will be summarized by study arm, and for the overall study include:

- Patient demographics: sex (number and percentage); age (median and IQR)

4.0 Main analyses

4.1 Outcome definitions

4.1.1 Primary outcome (Objective 1):

1) Primary outcome measure:

- Outcome measure: Mean score (1 point per symptom or sign, max score is 14) of major IMCI symptoms and signs assessed as observed by the clinical observer (Table 1).
- Comparator, and timing of measurement: Intervention health facilities using ePOCT+ versus control group (routine care)
- Method of measurement: Observation by the clinical observer using the adapted SPA observation of sick child survey
- Analysis type: Cluster level analysis, unpaired t-test for a difference in means

Table 1: Principal IMCI Symptoms and signs

Symptoms	Signs
Fever	Measured temperature
Cough or difficult breathing	Measured respiratory rate (in a child with cough or difficult breathing)
Child has had convulsions with this illness	Checked for pallor
Diarrhea	Weighed the child

Ear pain or discharge	Measured MUAC
Child is unable to drink or breastfeed	Height measured
Child vomits everything	Checked skin turgor for dehydration (e.g. pinch abdominal skin) – among children with diarrhea

Selection of principal IMCI symptoms and signs were based on what symptoms and signs can be feasibly assessed by the clinical observers, and guided by similar studies. Principal IMCI symptoms and signs are categorized as such based on clinical judgment. This composite group was defined a priori to data analysis. Omitted IMCI signs and symptoms include:

- "Lethargic and unconscious" was kept in the survey but not included in the principal IMCI signs and symptoms, as it is difficult to assess whether the clinician truly assessed this sign.
- "Convulsing now" as it is rare, is an observed sign not requiring special effort, and thus difficult to determine if it was observed or not by the clinician.
- "Look and listen for stridor or wheezing", similarly it would be difficult to know if the clinician actively listened for these signs.
- "Oedema of both feet", as it is rare, and omitted from ePOCT+ as decided by the Tanzanian expert panel.
- "Runny nose" or "red eyes", as it has no impact in the IMCI clinical algorithms other than for classification of measles that is rare. It can also be visually assessed, so difficult to know if the clinician actively looked for it.
- "Look for pus draining from the eye", "clouding of the cornea" were also excluded as this assessment would only be done if the child had measles within the last 3 months, and again visually assessed.
- Assessment of vaccination status, last dose of vitamin A or deworming medication, and child HIV status, as it is often assessed when reading the child's clinic/vaccination card. As such, only the reading of the child's health card will be assessed (table 2)

2) Secondary outcome measures:

2.1 Assessment of the principal IMCI symptoms and signs

- Outcome measure: Completed mean assessment of each individual principal IMCI symptoms and signs as observed by the clinical observer (table 1).
- Comparator and timing of measurement: Intervention health facilities using ePOCT+ versus control group (routine care)
- Method of measurement: Observation by the clinical observer using the adapted SPA observation of sick child survey
- Analysis type: Cluster level analysis, unpaired t-test

2.2 Assessment of other symptoms and signs

- Outcome measure: Completed assessment of each individual other symptoms and signs as observed by the clinical observer (table 2).
- Comparator, and timing of measurement: Intervention health facilities using ePOCT+ versus control group (routine care)

- Method of measurement: Observation by the clinical observer using the adapted SPA observation of sick child survey

-Analysis type: Cluster level analysis, unpaired t-test

2.3 Assessment of counselling

- Outcome measure: Counseling and explanations performed by the health care worker as observed by the clinical observer (table 3).

- Comparator, and timing of measurement: Intervention health facilities using ePOCT+ versus control group (routine care)

- Method of measurement: Observation by the clinical observer using the adapted SPA observation of sick child survey

- Analysis type: Cluster level analysis, unpaired t-test for the difference in means of the proportion of counselling and explanations performed.

4.1.2 Secondary outcomes (Secondary objectives 2.1 to 2.6):

2.1 To compare diagnosis made by clinicians

- Outcome measure: Distribution of diagnoses made by clinicians

- Comparator, and timing of measurement: Diagnoses made by clinicians in the intervention health facilities using ePOCT+ versus diagnoses made in control facilities during the second survey

-Method of measurement: Observation by the clinical observer using the adapted SPA observation of sick child survey

- Analysis type: Descriptive comparison

2.2 To compare the type, dosage and mode of administration of antibiotics and antimalarials

- Outcome measure: Distribution of medicines (molecules, not brands), dosage and mode of administration of antibiotics and antimalarials among children receiving an iv/oral antimicrobial

- Comparator and timing of measurement: Intervention health facilities versus the control facilities

- Method of measurement: Observation by the clinical observer using the adapted SPA observation of sick child survey

- Analysis type: Descriptive comparison

2.3 To determine the concordance between the diagnoses and antimicrobials prescribed

- Outcome measures:

2.3.1 Proportion of children treated appropriately with or without antibiotic based on the guidance for antibiotic treatment for individual diagnoses by IMCI or Tanzania national treatment guidelines. Antibiotic treatment will be deemed as appropriate in case there is atleast one diagnosis among several diagnoses that requires an antibiotic

- Comparator and timing of measurement: Appropriate antibiotic treatment between the intervention health facilities (Using *ePOCT+*) and control group (routine care)

- Method of measurement: Observation by the clinical observer using the adapted SPA observation of sick child survey

- Analysis type: Cluster level analysis, unpaired t-test

2.3.2 Appropriate antimalaria treatment (with or without antimalaria) based on the guidance for antimalaria treatment for malarial diagnoses by IMCI or Tanzanian national treatment guidelines. Antimalaria treatment will be deemed as appropriate in case there is at least a malaria diagnosis among several other diagnoses.

- Comparator and timing of measurement: Appropriate antimalaria treatment between the intervention health facilities (Using *ePOCT+*) and control group (routine care)
- Method of measurement: Observation by the clinical observer using the adapted SPA observation of sick child survey
- Analysis type: Cluster level analysis, unpaired t-test

2.4 To compare children for whom screening for priority conditions was performed (e.g., TB, HIV, malnutrition, vaccination status) and who were subsequently referred to a specialized program
Outcome measure:

- 2.4.1 Proportion of patients tested for HIV or Tuberculosis or malnutrition;
- 2.4.2 Among patients tested, proportion of Patients referred for TB, HIV, or malnutrition services
- Comparator and timing of measurement: Intervention health facilities using ePOCT+ versus control group (routine care)
- Method of measurement: Observation by the clinical observer using the adapted SPA observation of sick child survey
- Analysis type: Cluster level analysis, unpaired t-test

2.5 To evaluate the concordance between the proposed antibiotic treatments by ePOCT+, the accepted antibiotic treatments by the clinician, and the actual treatment prescribed among patients treated by using ePOCT+ algorithm.

- Outcome measure: Proposed antibiotic treatment by ePOCT+, accepted treatment by the clinician in the ePOCT+ system, and prescribed treatment by the clinician
- Comparator and timing of measurement: No comparator. As measured and observed in the intervention health facilities during the second and third survey.
- Method of measurement: Observation by the clinical observer using the adapted SPA observation of sick child survey. Records stored in the ePOCT+ tool.
- Analysis type: Descriptive analysis

2.6 *To compare antibiotic prescription*

- Outcome measure: Proportion of children prescribed at least one oral or IV/IMantibiotic.
- Comparator, timing: Intervention health facilities using ePOCT+ versus control group (routine care)
- Method of measurement: Observation by the clinical observer using the adapted SPA observation of sick child survey
- Analysis type: Cluster level analysis, unpaired t-test

4.2 Analysis methods

We will analyze all study outcomes using an intention-to-treat approach, i.e., all children with a recorded outcome will be included in the analysis and will be analyzed according to the group to which they were allocated.

Descriptive analysis

We will describe patient, clinician, and observer characteristics and outcomes by study arm and globally, using frequencies and percentages for categorical variables, means and standard deviations for normally distributed continuous variables, and median and range for skewed variables. Descriptive statistics will be provided at the individual and cluster levels.

Analyses of the outcome measures:

We will use a clustered-level analysis, based on cluster summaries, to analyze both primary and secondary outcome measures. This approach was chosen over an individual-level analysis due to the limited number of clusters (17,18). To analyse the primary outcome, a clustered-level analysis will be performed in two stages approach to adjust for both the cluster-level and individual-level covariates. In the first stage, we will fit a logistic regression model for cluster-level proportions and a linear regression for cluster-level means. Cluster level covariates selected a priori are the type of health facility, council, healthcare worker cadre, and healthcare worker years of experience. Individual level covariates selected a priori are patient age and sex. For the second stage, an unpaired t-test will be used to compare the residuals between study arms to estimate risk ratios and/or risk differences. Analysis of secondary outcome measures with a sufficient number of occurrences will follow a similar approach as for the primary outcome stated above.

Levels of confidence and p values:

Statistical tests and confidence intervals will be two-sided. Between-group comparisons will be calculated and presented with 95% confidence intervals wherever possible. The statistical significance level set will be at the 5% level.

Annex:

Table 2: Other Symptoms, signs and assessment

**Symptoms**	**Signs**	**Assessment**
Lethargic or unconscious	Felt behind ears	Looked at the child's health card
Asked duration of fever (if present)	Look in mouth	
Asked duration of cough or difficult breathing (if present)	Observed feeding	
Asked duration of diarrhea (if present)	Checked for neck stiffness (if febrile)	
Assessed HIV status of the mother	Pulse oximetry	

Assessed TB household contact	Undressed child to examine	
	Auscultated child's chest (if cough/difficult breathing)	

Table 3: Explanations and counseling

1	Inform the caregiver of the diagnosis(es)
2	Asked for normal feeding/breastfeeding habits when the child is not ill
3	Provided general information about feeding or breastfeeding the child even when not sick
4	Told the caregiver to give extra fluids to the child during this illness
5	Told caregiver to continue feeding / breastfeeding the child during this illness
6	Informed caregiver of signs / symptoms to bring child back immediately
7	Mentioned the child's weight or growth to the caretaker or discussed growth chart
8	Discussed follow-up visit for the sick child
9	Gave caregiver chance to ask questions during the counselling

References:

1. Arifeen SE, Hoque DME, Akter T, Rahman M, Hoque ME, Begum K, et al. Effect of the Integrated Management of Childhood Illness strategy on childhood mortality and nutrition in a rural area in Bangladesh: a cluster randomised trial. Lancet. 2009;374(9687):393–403.

2. Gera T, Shah D, Garner P, Richardson M, Sachdev HS. Integrated management of childhood illness (IMCI) strategy for children under five. Cochrane Database Syst Rev. 2016;(6).

3. Reñosa MD, Dalglish S, Bärnighausen K, McMahon S. Key challenges of health care workers in implementing the integrated management of childhood illnesses (IMCI) program: a scoping review. Glob Health Action. 2020;13(1):1732669.

4. Krüger C, Heinzel-Gutenbrunner M, Ali M. Adherence to the integrated management of childhood illness guidelines in Namibia, Kenya, Tanzania and Uganda: evidence from the national service provision assessment surveys. BMC Health Serv Res. 2017;17(1):1–12.

5. Johansson EW, Selling KE, Nsona H, Mappin B, Gething PW, Petzold M, et al. Integrated paediatric fever management and antibiotic over-treatment in Malawi health facilities: data mining a national facility census. Malar J. 2016;15:1–12.

6. Senn N, Rarau P, Salib M, Manong D, Siba P, Rogerson S, et al. Use of antibiotics within the IMCI guidelines in outpatient settings in Papua New Guinean children: an observational and effectiveness study. PLoS One. 2014;9(3):e90990.

7. Bessat C, Zonon NA, D'acremont V. Large-scale implementation of electronic Integrated Management of Childhood Illness (eIMCI) at the primary care level in Burkina Faso: a qualitative study on health worker perception of its medical content, usability and impact on antibiotic prescription and resistance. BMC Public Health. 2019;19:1–12.

8. Mitchell M, Hedt-Gauthier BL, Msellemu D, Nkaka M, Lesh N. Using electronic technology to improve clinical care - Results from a before-after cluster trial to evaluate assessment and classification of sick children according to Integrated Management of Childhood Illness (IMCI) protocol in Tanzania. BMC Med Inform Decis Mak. 2013;13(1).

9. Shao AF, Rambaud-Althaus C, Samaka J, Faustine AF, Perri-Moore S, Swai N, et al. New algorithm for managing childhood illness using mobile technology (ALMANACH): a controlled non-inferiority study on clinical outcome and antibiotic use in Tanzania. PLoS One. 2015;10(7):e0132316.

10. Keitel K, Kagoro F, Samaka J, Masimba J, Said Z, Temba H, et al. A novel electronic algorithm using host biomarker point-of-care tests for the management of febrile illnesses in Tanzanian children (e-POCT): a randomized, controlled non-inferiority trial. PLoS Med. 2017;14(10):e1002411.

11. Gilroy K, Winch PJ, Diawara A, Swedberg E, Thiero F, Kane M, et al. Impact of IMCI training and language used by provider on quality of counseling provided to parents of sick children in Bougouni District, Mali. Patient Educ Couns. 2004;54(1):35–44.

12. Rowe AK, Onikpo F, Lama M, Osterholt DM, Rowe SY, Deming MS. A multifaceted intervention to improve health worker adherence to integrated management of childhood illness guidelines in Benin. Am J Public Health. 2009;99(5):837–46.

13. Lemiengre MB, Verbakel JY, Colman R, De Burghgraeve T, Buntinx F, Aertgeerts B, et al. Reducing inappropriate antibiotic prescribing for children in primary care: a cluster randomised controlled trial of two interventions. Br J Gen Pract. 2018;68(668):e204–10.

14. Flottorp S, Oxman AD, Håvelsrud K, Treweek S, Herrin J. Cluster randomised controlled trial of tailored interventions to improve the management of urinary tract infections in women and sore throat. Bmj. 2002;325(7360):367.

15. Bernasconi A, Crabbé F, Adedeji AM, Bello A, Schmitz T, Landi M, et al. Results from one-year use of an electronic Clinical Decision Support System in a post-conflict context: An implementation research. PLoS One. 2019 Dec 1;14(12).

16. Bernasconi A, Crabbé F, Raab M, Rossi R. Can the use of digital algorithms improve quality care? An example from Afghanistan. PLoS One. 2018 Nov 1;13(11).

17. Thompson JA, Leyrat C, Fielding KL, Hayes RJ. Cluster randomised trials with a binary outcome and a small number of clusters: comparison of individual and cluster level analysis method. BMC Med Res Methodol. 2022 Dec 1;22(1).

18. By Richard J. Hayes LHM. Cluster randomised trials. Chapman & Hall; 2017. 199 p.
