# Supplementary material for: A cluster randomized trial assessing the effect of a digital health algorithm on quality of care in Tanzania (DYNAMIC study)
Source: PLOS Digit Health. 2024 Dec 23;3(12):e0000694. doi: 10.1371/journal.pdig.0000694 (PMC11666054; doi:10.1371/journal.pdig.0000694)
Supplement: Attachment — Submitted filename: DYN TZ cRCT quality of care - Response to reviewers.pdf [file pdig.0000694.s004.pdf]

4 October 2024

RE : Response to reviewers

PDIG-D-24-00255: A cluster randomized trial assessing the effect of a digital health algorithm on quality of care in Tanzania (DYNAMIC study)

Dear Shannon Freeman and the PLOS DH editorial team,

We thank you and the reviewers for the thoughtful and highly constructive comments and suggestions. We agreed with all points, and have made a point-by-point response along with the revised manuscript.

We feel that the manuscript is stronger thanks to these modifications, and we thank you for giving us the opportunity to improve our work.

Best regards,

Rainer Tan

unisanté
Centre universitaire de médecine générale et santé publique · Lausanne

NATIONAL INSTITUTE FOR MEDICAL RESEARCH

IFAKARA HEALTH INSTITUTE
research | training | services

Swiss TPH
Swiss Tropical and Public Health Institute
Schweizerisches Tropen- und Public Health-Institut
Institut Tropical et de Santé Publique Suisse
Associated Institute of the University of Basel

Response to editor and journal requirements

1. Please review your reference list to ensure that it is complete and correct. If you have cited papers that have been retracted, please include the rationale for doing so in the manuscript text, or remove these references and replace them with relevant current references. Any changes to the reference list should be mentioned in the rebuttal letter that accompanies your revised manuscript. If you need to cite a retracted article, indicate the article's retracted status in the References list and also include a citation and full reference for the retraction notice.	References were reviewed.<br><br>Two changes were made:<br>1) Horwood C, Haskins L, Mapumulo S, Connolly C, Luthuli S, Jensen C, et al. Electronic Integrated Management of Childhood Illness (eIMCI): a randomized controlled trial to evaluate an electronic clinical decision-making support system for management of sick children in primary health care facilities in South Africa. BMC Health Services Research. 2024;24(1):177. doi: 10.1186/s12913-024-10547-6.<br><br>Was replaced with the published reference:<br>Horwood C, Haskins L, Mapumulo S, Connolly C, Luthuli S, Jensen C, et al. Electronic Integrated Management of Childhood Illness (eIMCI): a randomized controlled trial to evaluate an electronic clinical decision-making support system for management of sick children in primary health care facilities in South Africa. BMC Health Services Research. 2024;24(1):177. doi: 10.1186/s12913-024-10547-6.<br><br>2) The year of publication was updated to 2024 to align with the date of the published paper (not the digital release) for this reference:<br>Tan R, Kavishe G, Luwanda LB, Kulinkina AV, Renggli S, Mangu C, et al. A digital health algorithm to guide antibiotic prescription in pediatric outpatient care: a cluster randomized controlled trial. Nature Medicine. 2024;30(1):76-84. doi: 10.1038/s41591-023-02633-9.
This is a very interesting paper which will be of great interest to a wide readership both within Tanzania and internationally. The reviewers have provided some helpful suggestions for minor edits. Additionally, this paper would be strengthened with some careful proofreading and attention to spelling, grammar, and punctuation.	We thank the editor for the positive response.<br><br>We have made some corrections to spelling, grammar, and punctuation, documented in the "track changes" version of the manuscript.
**Reviewer #1**	
A topic and clinically relevant article concerning a cluster randomized trial assessing the effect of a digital health algorithm on quality of care in Tanzania	We thank the reviewer for seeing the relevance and value of our study and intervention.

(DYNAMIC study) indicating the potential value of such technologies.	
**Reviewer #2**	
1.The study has certain significance and application value for the health management of a certain population with this digital CDSA .	We thank the reviewer for this positive response.
2.Please indicate the detailed source of the statistical software, as well as the scope of application and reference standards of the statistical methods used in this study.	A reference was added for the statistical software.<br><br>We have clarified the scope of application and reference standards of the statistical methods used in this study (the underlined text was added):<br><br>“All analyses were performed using a clustered-level analysis approach instead of an individual-level analysis due to the small number of clusters included [1, 2]. <u>Such an approach has been shown to be robust in cluster randomized trials with less than 30 clusters, with cluster sizes of >10, when assessing outcomes with a prevalence of > 10%, and performs best when cluster sizes are similar, and in case of high ICC [1, 2].”</u>
3. Some language writing (spelling and grammatical) needs to be corrected in the manuscript. Some sentences are too long and difficult to understand.	Spelling and grammar was reviewed and corrected visible in the track version submission.
**Reviewer #3**	
A cluster randomized trial assessing the effect of a digital health algorithm on quality of care in Tanzania (DYNAMIC study)," several areas need attention. First, the introduction should clearly articulate the specific research gap this study addresses and succinctly state the primary objectives and research questions. This will help readers understand the importance and relevance of the study within the broader field of digital health interventions.	We agree with the reviewer that the introduction should clearly articulate the specific research gap, and the implementation gap.<br><br>This research gap is outlined in the second paragraph of the introduction, in which we outline that while digital tools were developed to enhance adherence to clinical guidelines, the studies evaluating these tools were often conducted in controlled study settings and most lacked randomization. To make sure this is more explicit we have modified the word to clearly state that this is the research gap:<br><br>While several studies have found that using these digital CDSAs improve adherence to IMCI, a noteworthy <u>research gap</u> is that many of these investigations were conducted in controlled study settings, and most lacked randomization [3-10].
Additionally, more comprehensive details should be provided on the ePOCT+ digital clinical decision support algorithm (CDSA), including its functionalities and how it was integrated into routine care. This should also cover the training and mentorship components of the intervention, which are critical to understanding the	The intervention is described in the section “Interventions” on page 5-6. Some modifications were made in order to provide more information and clarity:<br><br>“The intervention involved equipping health facilities with ePOCT+, an electronic clinical decision support algorithm on an Android based tablet (Fig 1), along with associated point-of-care

intervention's implementation and effectiveness.	tests (C-Reactive Protein, Hemoglobin, pulse oximetry), training, and mentorship. ePOCT+ prompts the healthcare provider to answer questions about demographics, symptoms, signs, and tests [11]. Based on the answers, ePOCT+ proposes one or more diagnoses, treatments, and management plans, including referral recommendation. Healthcare providers had the possibility to deviate from ePOCT+ recommendations, and had the option to not use the tool. In order to move forward within the different sections of the digital tool, it was mandatory to respond to all IMCI symptoms and signs, except for height and mid-upper arm circumference (MUAC) which was optional. The tool allowed some signs to be estimated (temperature, respiratory rate) or based on recent measurements (weight). Detailed description on the development process and features of ePOCT+ and the medAL-reader application can be found in separate publications [11].<br><br>**Figure 1: Screen-shots of different stages of ePOCT+ running on the medAL-reader application.** Stages are shown in order of appearance, however not all stages are shown in the figure.<br><br>The implementation team provided mentorship to intervention health facilities. This mentoring consisted of regular visits to health facilities every 2-3 months, and frequent communication via phone calls or group messages (3-4 times per month) to address issues, offer guidance, and gather feedback on the new tools. Quality-of-care dashboards were shared through group messages, enabling healthcare providers to compare their antibiotic prescription rates, uptake, and other quality-of-care indicators with other facilities (benchmarking). Control health facilities continued with usual care, did not have access to clinical data dashboards, and only received visits from the implementation team to help resolve issues related to the electronic case report forms (eCRFs).<br><br>The infrastructure provided to all health facilities (control and intervention) included a tablet for each outpatient consultation room, a router, a local server (Raspberry Pi), internet connectivity, and backup power (battery or solar system if needed). If unavailable weighing scales, mid-upper arm circumference (MUAC) bands, and thermometers were provided to all health facilities. Healthcare providers from both intervention and control facilities underwent equivalent clinical refresher training on IMCI and concepts of antibiotic stewardship. Additionally, specific training was provided on the use of the ePOCT+ CDSA in intervention facilities and the use of the eCRF in control facilities.
The statistical methods section would benefit from a more detailed	In line with your comment, and that of reviewer 2, we have modified the analysis section of the methods to provide more

<table>
<tr>
<td>explanation, including how missing data were handled and the rationale for using specific statistical models and covariates. This will enhance the robustness of the methodology and make it easier for readers to assess the validity of the findings.</td>
<td>information on the statistical model, the handling of missing data, and the choice of covariates. Modifications underlined:

All analyses were performed using an intention-to-treat approach, i.e. all children with a recorded outcome were included in the analysis regardless if the intervention, ePOCT+, was used or not. The eCRF was designed to prevent missing data, as such all data was complete. All analyses were performed using a clustered-level analysis approach instead of an individual-level analysis due to the small number of clusters included [1, 2]. Such an approach has been shown to be robust in cluster randomized trials with less than 30 clusters, with cluster sizes of >10, when assessing outcomes with a prevalence of > 10%, and performs best when cluster sizes are similar, and in case of high ICC[1, 2]. The analysis was performed using a two-stage approach as outlined by Hayes et al [2, 12], to adjust for both the cluster-level and individual-level covariates. In the first stage, we used a logistic regression model for binary outcomes and linear regression model for continuous outcomes adjusting for covariates and ignoring clustering and trial arm. Cluster-level residuals were then calculated for each cluster. In the second stage the residuals were compared to estimate risk ratios for the binary outcomes and mean risk difference for the continuous outcome (including the primary outcome) between study arms. Pre-specified cluster level covariates were type of health facility, council, healthcare worker cadre, and healthcare worker years of experience. Pre-specified individual level covariates of patients were age and sex. Covariates were pre-specified based on their potential influence on the study outcomes. All analyses were performed using Stata v16, v17, and v18 [13].</td>
</tr>
<tr>
<td>Furthermore, the presentation of results could be improved by using more visual aids, such as tables and figures, to summarize key outcomes and differences between the intervention and control groups. This approach would make the results more accessible and engaging.</td>
<td>We agree with the reviewer as such we have added figure 4 which graphically presents data from table 2:
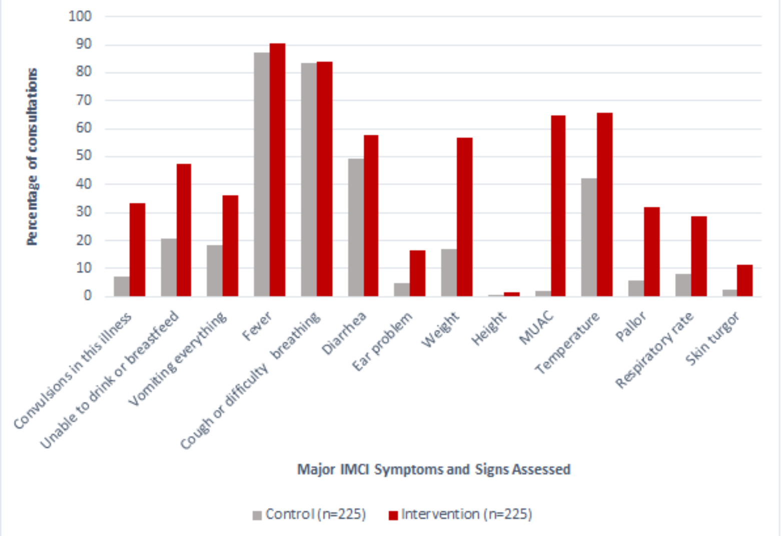
</td>
</tr>
</table>

	And have added figure 5, which graphically presents data from table 5: 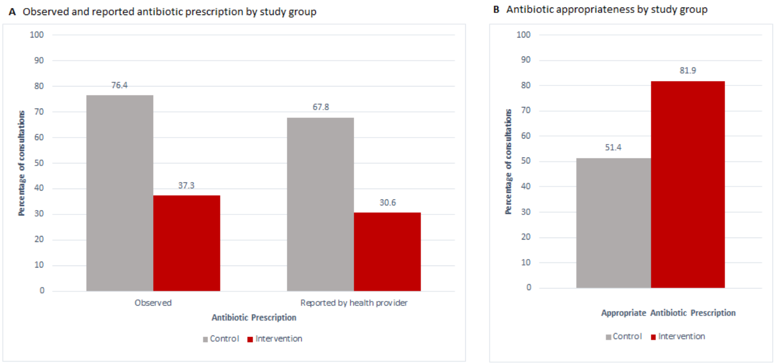 This is in addition to Table 2, table 3, table 4, table 5 and figure 3 that illustrate the results.
In the discussion section, a deeper exploration of the study's findings and comparisons with similar studies would be valuable. The authors should provide potential explanations for the observed outcomes and discuss the implications for future digital health implementations.	In the discussion we compare the results of our study in comparison to 5 other studies, and provide explanations for the observed outcomes and implications for future digital health implementations. This is presented in paragraph 2-6 in the discussion.
Moreover, a thorough discussion of the study's limitations, including potential biases and their impact on the findings, is necessary for transparency and to guide future research.	4 major limitations are discussed in the discussion including potential biases and their impact on the findings. This is presented in paragraph 7 in the discussion.
Consistency in terminology and formatting throughout the manuscript will also enhance clarity and readability.	Corrections were provided in the updated manuscript.
Finally, a detailed data availability statement should be included to specify where and how the study data can be accessed, promoting transparency and reproducibility. Addressing these areas will significantly strengthen the manuscript, making it a more compelling and impactful contribution to the field of digital health.	A detailed data availability statement is provided along with a link that is available to access the data: “De-identified data can be found at 10.5281/zenodo.10849644”

References

1. Thompson JA, Leyrat C, Fielding KL, Hayes RJ. Cluster randomised trials with a binary outcome and a small number of clusters: comparison of individual and cluster level analysis method. BMC Medical Research Methodology. 2022;22(1):1-15.
2. Hayes RJ, Moulton LH. Cluster randomised trials: CRC press; 2017.
3. Mitchell M, Hedt-Gauthier BL, Msellemu D, Nkaka M, Lesh N. Using electronic technology to improve clinical care - results from a before-after cluster trial to evaluate assessment and classification of sick children according to Integrated Management of Childhood Illness (IMCI) protocol in Tanzania. BMC medical informatics and decision making. 2013;13:95. Epub 2013/08/29. doi: 10.1186/1472-6947-13-95. PubMed PMID: 23981292; PubMed Central PMCID: PMCPMC3766002.
4. Rambaud-Althaus C, Shao A, Samaka J, Swai N, Perri S, Kahama-Maro J, et al. Performance of Health Workers Using an Electronic Algorithm for the Management of Childhood Illness in Tanzania: A Pilot Implementation Study. The American journal of tropical medicine and hygiene. 2017;96(1):249-57. Epub 2017/01/13. doi: 10.4269/ajtmh.15-0395. PubMed PMID: 28077751; PubMed Central PMCID: PMCPMC5239703.
5. Horwood C, Haskins L, Mapumulo S, Connolly C, Luthuli S, Jensen C, et al. Electronic Integrated Management of Childhood Illness (eIMCI): a randomized controlled trial to evaluate an electronic clinical decision-making support system for management of sick children in primary health care facilities in South Africa. BMC Health Services Research. 2024;24(1):177. doi: 10.1186/s12913-024-10547-6.
6. Sarrassat S, Lewis JJ, Some AS, Somda S, Cousens S, Blanchet K. An Integrated eDiagnosis Approach (IeDA) versus standard IMCI for assessing and managing childhood illness in Burkina Faso: a stepped-wedge cluster randomised trial. BMC health services research. 2021;21(1):354-. doi: 10.1186/s12913-021-06317-3. PubMed PMID: 33863326.
7. Bernasconi A, Crabbé F, Adedeji AM, Bello A, Schmitz T, Landi M, et al. Results from one-year use of an electronic Clinical Decision Support System in a post-conflict context: An implementation research. PLoS One. 2019;14(12):e0225634. Epub 2019/12/04. doi: 10.1371/journal.pone.0225634. PubMed PMID: 31790448; PubMed Central PMCID: PMCPMC6886837.
8. Bernasconi A, Crabbé F, Rossi R, Qani I, Vanobberghen A, Raab M, et al. The ALMANACH Project: Preliminary results and potentiality from Afghanistan. International journal of medical informatics. 2018;114:130-5.
9. Beynon F, Salzmann T, Faye PM, Thiongane A, Ndiaye O, Luwanda LB, et al. Paediatric digital clinical decision support for global health. Revue Medicale Suisse. 2023;19(836):1398-403.
10. Agarwal S, Glenton C, Tamrat T, Henschke N, Maayan N, Fønhus MS, et al. Decision-support tools via mobile devices to improve quality of care in primary healthcare settings. Cochrane Database Syst Rev. 2021;7(7):Cd012944. Epub 2021/07/28. doi: 10.1002/14651858.CD012944.pub2. PubMed PMID: 34314020; PubMed Central PMCID: PMCPMC8406991.
11. Tan R, Cobuccio L, Beynon F, Levine GA, Vaezipour N, Luwanda LB, et al. ePOCT+ and the medAL-suite: Development of an electronic clinical decision support algorithm and digital platform for pediatric outpatients in low- and middle-income countries. PLOS Digital Health. 2023;2(1):e0000170. doi: 10.1371/journal.pdig.0000170.
12. Thompson JA, Leurent B, Nash S, Moulton LH, Hayes RJ. Cluster randomized controlled trial analysis at the cluster level: The clan command. The Stata Journal. 2023;23(3):754-73.
13. StataCorp. Stata Statistical Software: Release 16. College Station, TX: StataCorp LLC.; 2019.
